# Sulfur as a proxy for identifying coast-inland human mobility in Northern Iberia during Late Prehistory

**DOI:** 10.1371/journal.pone.0330249

**Published:** 2025-08-28

**Authors:** Borja González-Rabanal, Marco Vidal-Cordasco, Jennifer R. Jones, Lucía Agudo Pérez, Eduardo Carmona-Ballestero, Belén López, Miguel Ángel Martín Merino, Ana Isabel Ortega, Lawrence G. Straus, Rhiannon E. Stevens, Cristina Vega-Maeso, Manuel R. González Morales, Ana B. Marín-Arroyo

**Affiliations:** 1 Grupo I+D+i EvoAdapta, (Evolución Humana y Adaptaciones durante la Prehistoria), Departamento de Ciencias Históricas, Universidad de Cantabria, Santander, Spain; 2 Departamento de Prehistoria, Arqueología, Antropología Social y Ciencias y Técnicas Historiográficas, Universidad de Valladolid, Valladolid, Spain; 3 Research Centre For Field Archaeology and Forensic Taphonomy, School of Law and Policing, University of Lancashire, Lancashire, Preston, United Kingdom; 4 Servicio Territorial de Cultura, Turismo y Deporte de Valladolid, Junta de Castilla y León, Valladolid, Spain; 5 Universidad de Burgos, Burgos, Spain; 6 Departamento de Biología de Organismos y Sistemas, Universidad de Oviedo, Oviedo, Spain; 7 Sociedad Española de Espeleología y Ciencias del Karst, Fundación Gómez Pardo, Madrid, Spain; 8 Real Academia Burgense de Historia y Bellas Artes, Institución Fernán González, Burgos, Spain; 9 Department of Anthropology, University of New Mexico, Albuquerque, New Mexico, United States of America; 10 UCL Institute of Archaeology, University College London, London, United Kingdom; 11 Servicio Territorial de Cultura, Turismo y Deporte de Segovia, Junta de Castilla y León, Valladolid, Spain; 12 Instituto Internacional de Investigaciones Prehistóricas de Cantabria, Universidad de Cantabria, Banco Santander, Gobierno de Cantabria, Santander, Spain; University of Padova: Universita degli Studi di Padova, ITALY

## Abstract

Population movements constitute a significant driver of cultural change in prehistoric societies. In recent years, sulfur isotopes have emerged as a valuable approach for distinguishing human/animal provenance. However, the scarcity of sulfur isotope studies and the lack of baseline maps predicting their variations in the landscape limit our current knowledge about mobility behaviours. Here, we first present the δ^34^S isotope values of 142 human and animal bone collagen samples from coastal and inland funerary sites located in northern Iberia. Second, to apply a multivariate machine-learning regression and a random forest model to predict sulfur isotope variations across Iberia, we compiled the sulfur isotope data from 554 specimens of 41 archaeological locations from Holocene contexts. Our research demonstrated that population movement between coastal and inland locations is observable through differences in the δ^34^S isotope values of individuals linked to their respective environments, suggesting migrations on both sides of the Cantabrian mountain range. The resulting isoscape model demonstrates that sulfur isotope patterns are highly predictable, with 82% of the sulfur isotope variation explained by only four variables: elevation, Bouguer anomaly, distance from the coast, and strontium isotope values. While the model is highly accurate for regions with large amounts of data, such as northern Iberia, Central and Eastern Iberia still require more sulfur isotope data to predict isoscapes.

## 1. Introduction

Sulfur-stable isotopes measured on bone collagen have been proven a helpful tool for reconstructing past dietary patterns, trophic relationships, environmental conditions and geographical origins [1]. Integrating sulfur isotope data with other stable isotopes and archaeological evidence allows a more comprehensive understanding of past ecosystems and human-animal interactions through time [2]. However, sulfur isotope analyses from archaeological contexts are still relatively scarce. Initially, sulfur isotopes were used for dietary reconstruction of prehistoric human groups due to variations in the sulfur isotopic composition of plants, marine organisms, and terrestrial animals [3–6]. This expanded on δ^13^C and δ^15^N isotope evidence as the δ^34^S isotope values can help identify aquatic resource consumption and can distinguish between freshwater or marine food webs [7,8]. However, the isotopic fractionation is low in sulfur (0–1‰) [9]. Later, sulfur isotopes were implemented to identify environmental and climatic changes [10–13], by analysing their variations over time and their potential impact on ancient ecosystems. Nevertheless, perhaps the most promising application in the archaeological discipline is its use as a mobility indicator to identify migration or territoriality phenomena of humans [14–16] and animals [17–20] with values directly linked to its local geology, soil type, proximity to the sea and rainfall at locations near the coast [21]. Particularly, sulfur has been used in multi-isotope investigations, especially along with strontium, to assess human and animal provenance [22–25]

Like δ^13^C and δ^15^N isotope values, the δ^34^S stable isotope composition of bone collagen reflects long-term dietary average over the last years of life [26]. Sulfur in bone collagen originates from the foods consumed by an individual [27], which is ultimately derived from the sulfur present in the landscape in which it lived [28]. Thus, sulfur isotopes can be used to understand past human and animal behaviours. Factors including geographical location, local geology, climate, soil hydrology, and dietary choices can influence observed δ^34^S isotope values, providing valuable insights into the lives of past organisms. Environmental sulfur in the atmosphere and biosphere originates from marine and terrestrial sulfur moving through the water cycle, erosion, and tectonic cycles [1]. Bioavailable sulfur is taken up by plants through water in the soil or sulfur in the atmosphere [29]. Once incorporated into proteins, sulfur passes through the food chain to animals and, finally, to humans [30]. The complexity of the sulfur cycle, with isotope values ranging approximately between −20‰ (even lower) and +30‰ [31], offers opportunities for using sulfur to explore archaeological issues. In marine locations, oceanic sulfur is re-deposited as rain over coastal platforms, reaching around 30 km to the coast due to the sea spray effect [32]. For all of these reasons, sulfur is capable of differentiating isozones (defined as areas with distinct natural or anthropogenic isotope baselines [33]) and, therefore, can provide clues into an organism’s geographic origin, being particularly useful in archaeological studies for tracing human and animal movements across landscapes [21].

The use of sulfur isotopes to differentiate between terrestrial ecosystems and coastal-inland locations in the Cantabrian Region (northern Atlantic Iberia) has yielded promising insights concerning animal/human mobility, already proposed through pilot studies within Palaeolithic fauna [34,35] and Late Neolithic and Chalcolithic humans [16,36]. These findings revealed remarkable differences in the human and faunal stable isotope values, likely associated with the movement of people. In this study, bone collagen sulfur isotope analysis (δ^34^S) was conducted on 74 human and 68 animal specimens from 17 late prehistoric funerary sites, dated between the Late Neolithic and Late Bronze Age (3800−800 cal. BC), to explore the mobility patterns of farming communities in northern Iberia. In order to determine whether the observed changes were due to dietary behaviour (i.e., consumption of marine resources) or environmental factors (i.e., proximity to the sea), published and unpublished δ^13^C and δ^15^N values from the same individuals were considered along with δ^34^S isotope values. Moreover, we compiled sulfur isotope data from previously published works and applied a multivariate machine-learning regression to predict sulfur isotope variability, ultimately generating a sulfur isoscape of Iberia.

## 2. Materials

**Fig 1:**
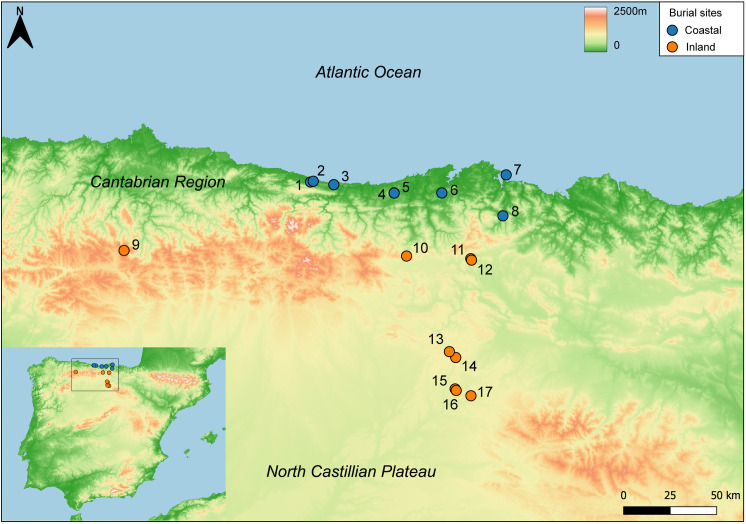
Location of the burial sites studied in this work: 1. El Hondón; 2. La Llana; 3. El Espinoso; 4. Los Avellanos I; 5. Los Avellanos II; 6. El Abrigo de la Castañera; 7. La Fragua; 8. El Mirón; 9. Los Cinchos; 10. El Agua; 11. Kaite; 12. Palomera; 13. Trulla; 14. La Quebrantada; 15. Arroyal I; 16. Fuente Celada; 17. El Hornazo. The blue and orange colours show the classification of each site according to the geospatial distribution.

The samples included in this study come from burial caves, megalithic monuments, and pit fields located in coastal and inland areas of the Cantabrian Region, the high Ebro valley and the North Castilian Plateau (Fig 1). Coastal/inland sites were defined as locations less/more than 30 km from the current coastline, with no mountains exceeding 500 meters above sea level in between. The Cantabrian Region is a Eurosiberian biogeographical region separated from the Spanish central plateau and the Ebro valley by steep, high mountains, running parallel to the coast and reaching up to 2500m above sea level in their highest sectors. It has an Atlantic climate and lies close to the coast with year-round rainfall and relatively limited seasonal temperature variation [37]. On the other hand, the high Ebro valley and the North Castilian Plateau are part of the Mediterranean biogeographical region with a cool, dry, continental inland climate. This area has long and cold winters, short and warm summers, and a strong contrast between day and night temperatures [38]. The coastal sites studied are El Hondón, La Llana and El Espinoso (Asturias); and Los Avellanos I and II, El Abrigo de la Castañera, La Fragua and El Mirón (Cantabria), and the inland sites are Los Cinchos (Asturias); El Agua (Cantabria); and Kaite, Palomera, La Quebrantada, Trulla, Arroyal I, Fuente Celada and El Hornazo (Burgos). The specific environmental conditions of both regions hold potential for identifying models of mobility behaviour in humans and animals during Late Prehistory. A summary of the samples analysed from each site is presented in Table 1, and detailed information about the sites is included in the S1 Text.

**Table 1:** Summary of the human and animal species sampled for δ^13^C, δ^15^N and δ^34^S analysis from each site and region.

Site	Burial type	Date cal BC 2σ	Culture	Humans	Animals	References
*Coastal sites*						
El Espinoso	Cave	1200−1100	Late Bronze Age	14	2	González-Rabanal et al 2017; 2022
La Llana	Cave	1700−1500	Middle Bronze Age	1		Vega-Maeso 2015; González-Rabanal et al 2022
La Fragua	Cave	2200−2000	Early Bronze Age	1		González-Rabanal 2022; González-Rabanal et al 2022
El Abrigo de la Castañera	Rockshelter	2300−1750	Early Bronze Age	3	4	Vega-Maeso 2015; Jones et al 2019
El Mirón	Cave	1900−1550	Middle Bronze Age	1		González-Rabanal 2022; González Morales et al 2024
		2450−2200	Chalcolithic	1		
Los Avellanos II	Cave	2900−2600	Chalcolithic	4	2	Vega-Maeso 2015; González-Rabanal et al 2020
Los Avellanos I	Cave	2850−2600	Chalcolithic	2	1	
		3500−3350	Late Neolithic	1		
El Hondón	Cave	3350−2900	Late Neolithic	3	4	González-Rabanal 2022
***Total***				**31**	**13**	
*Inland sites*						
Trulla	Cave	1000−800	Late Bronze Age	1		Unpublished
		2900−2700	Chalcolithic	1		
El Agua	Cave	1750−1500	Middle Bronze Age	1		González-Rabanal 2022
Palomera	Cave	2000−1750	Early Bronze Age	2		González-Rabanal et al 2023
Los Cinchos	Cave	2000−1750	Early Bronze Age	1		Alonso-Llamazares and López 2018; García de Castro y Busto Hevia, 2018
Fuente Celada	Pits field	2850−2450	Chalcolithic	3	8	Alameda Cuenca-Romero et al 2011; Jones et al 2019
El Hornazo	Pits field	2850−2450	Chalcolithic	2	15	Carmona-Ballestero et al 2013; Jones et al 2019
Kaite	Cave	2450−2300	Chalcolithic	1		González-Rabanal 2022
		3100−2900	Late Neolithic	3	7	
Arroyal I	Dolmen	2450−2200	Chalcolithic	7	7	Carmona-Ballestero et al 2014; Jones et al 2019
		3300−2900	Late Neolithic	2		
La Quebrantada	Cave	1450−1000	Late Bronze Age	4	5	Unpublished
		1750−1300	Middle Bronze Age	5		
		2000−1550	Early Bronze Age	5		
		2500−2200	Chalcolithic	1		
		3750−2950	Late Neolithic	4		
***Total***				**43**	**42**	

## 3. Methods

### 3.1. Sampling strategy

The sampling strategy was designed based on the MNI of each site, both in humans and animals. The animal taxa sampled include mainly herbivores, with a small portion of omnivores and carnivores used for comparison. Adult individuals have been preferably selected, although in some cases, sub-adult individuals have also been sampled. Contextual and osteological individual information is reported in the S1 Table. Prior to any sampling, ethical approval was obtained from the Museum of Burgos (Burgos) and the Museum of Prehistory and Archaeology of Cantabria (Santander). All necessary permits were obtained for the described study, which complied with all relevant regulations. Sample selection was based on three main criteria: 1) selection of the bone that provided the greater MNI according to side, age and size; 2) preferable choice of long bones with a dense cortical to ensure the high-quality standard of collagen preservation; 3) well-preserved remains not affected by taphonomic processes; and 4) in case of faunal samples, bones with anthropogenic modifications linked with butchering processes. Sampling was based on the extraction of ~1 g of bone using a low-vibration micromotor with a diamond-edge cutting wheel in dedicated clean spaces using gloves, mask, security glasses and lab coat under an extractor hood to avoid contamination.

### 3.2. Bone collagen extraction and isotope analysis

Sample preparation was undertaken at the EvoAdapta Group (University of Cantabria) facilities, where bone collagen extraction was undertaken according to the procedures proposed by Richards and Hedges [39]. This method involves the following steps: 1) cleaning of the bone fragments (0.6–0.8 g) by abrasion to remove any possible contamination; 2) demineralisation of the samples in 0.5 M HCl at 6–8 °C for 3–10 days; 3) washing using de-ionized water; 4) gelatinisation of the samples in pH 3 HCL at 70 °C for 48 h; 5) filtration with 5–8 μm Ezee® filters; 6) freeze-drying the filtrate. Samples were separately analysed for δ^13^C/δ^15^N and δ^34^S using a Europa ScientificTM elemental analyser, coupled to a mass spectrometer (EA-IRMS) at Iso-Analytical laboratory (Crewe, UK). One in every five samples was measured by duplicate to ensure their reliability.

The δ^13^C, δ^15^N and δ^34^S isotope values were reported relative to the V-PDB, AIR and VCDT international standards, respectively. The reference material used for carbon and nitrogen isotope analysis of the collagen samples was IA-R068 (soy protein, δ^13^C= -25.22‰, δ^15^N = 0.99‰). IA-R068, IA-R038 (L-alanine, δ^13^C = −24.99‰, δ^15^N = −0.65‰), IA-R069 (tuna protein, δ^13^C = −18.88‰, δ^15^N = 11.60‰) and a mixture of IAEA-C7 (oxalic acid, δ^13^C = −14.48‰) and IA-R046 (ammonium sulfate, δ^15^N = 22.04‰) were run as quality control check standards. IA-R068, IA-R038 and IA-R069 are calibrated against and traceable to IAEA-CH-6 (sucrose, δ^13^C = −10.45‰) and IAEA-N-1 (ammonium sulfate, δ^15^N = 0.40‰). IA-R046 is calibrated against and traceable to IAEA-N-1. IAEA-C7, IAEA-CH-6 and IAEA-N-1 are interlaboratory comparison standards distributed by the International Atomic Energy Agency, Vienna. The reference material used for sulfur isotope analysis of the collagen samples was IA-R061 (barium sulfate, δ^34^S= 20.33‰). IA-R061, IA-R025 (barium sulfate, δ^34^S= 8.53‰) and IA-R026 (silver sulfide, δ^34^S= 3.96‰) were used for calibration and correction of the ^18^O contribution to the SO+ ion beam. IA-R061, IA-R025 and IA-R026 are in-house standards calibrated against and traceable to NBS-127 (barium sulfate, δ^34^S= 20.3‰) and IAEA-S-1 (silver sulfide, δ^34^S= -0.30‰). IA-R061, IAEA-S-1, IA-R068 (soy protein, δ^34^S= 5.25‰) and IA-R069 (tuna protein, δ^34^S= 18.91‰) were measured as quality control check standards during the batch analysis of the collagen samples. IA-R068 and IA-R069 are in-house standards calibrated against and traceable to NBS-127 and IAEA-SO-5 (barium sulfate, δ^34^S= 0.50‰). NBS-127, IAEA-S-1 and IAEA-SO-5 are inter-laboratory comparison standards distributed by the International Atomic Energy Agency (IAEA) with internationally accepted δ^34^S isotope values. Habitually established quality indicators were used: %Col (>1), %C (30–44%), %N (11–16%), %S (0.15–0.35%), C:N (2.9–3.6), C:S (600 ± 300) and N:S (200 ± 100) [40–43].

All statistical tests were undertaken using the R software [44,45]. First, we compared human and animal δ^13^C, δ^15^N and δ^34^S stable isotope values from coastal and inland funerary sites. Then, we analysed the trends of each isotope over a chronocultural sequence in both biogeographic regions. A Spearman’s correlation test was used to analyse significant relationships between isotopes results [46]. A p-value of <0.05 or less was indicative to be statistically significant. Finally, a Wilcoxon-Mann-Whitney U test [47] with a post-hoc Holm-Bonferroni correction [48] was conducted in these isotope groupings. A p-value of <0.05 or less was deemed to be statistically different. The R scripts and associated data to replicate the stable isotope figures, correlations, and statistical comparisons are available in this link osf.io/zmg9d.

### 3.3. Sulfur isoscape reconstruction

The δ^34^S modelling framework, R code, and raster predictors used in this work are adapted from Bataille et al. (2021) [2] with a region-specific adaptation for the study area. Following the structure of the European sulfur database compiled by the aforementioned work and incorporating new published sites, we gathered all the δ^34^S data published in human (*n* = 248) and animal (*n* = 164) collagen across Iberia during the Holocene [14,16,36,49–56], which is a relatively stable period with minor geological and climatic changes. The region, site name, species, and archaeological period, in addition to the latitude and longitude for each sample, were recorded according to the literature. In total, 412 sulfur isotope data from 24 additional Iberian archaeological sites were available for adding to the database. A total of 21 variables that are expected to affect the δ^34^S isotope values according to previous studies were selected [2,57]. These independent variables encompass geology, elevation, climate, soil properties, aerosol deposition, and proximity to the coast, and they show different resolution scales (Table 2). All are available as raster layers in this link: osf.io/zmg9d. To isolate the local individuals’ signals at each site, we excluded samples defined as non-local individuals based on archaeological evidence reported in publications. In order to assess diagenetic alteration of bone collagen, we also eliminated individuals whose atomic C:S and N:S ratios were outside the accepted margins. Finally, we omitted samples with only sulfur isotope values.

**Table 2:** List of geological, climatic, environmental and anthropogenic variables used in the multivariate regression.

Variable	Description	Resolution	References
r.age	Terrane age attribute (Myrs)	1 km	Mooney et al 1998
r.ai	Global Aridity Index	30-arc sec	Trabucco and Zomer 2019
r.bouger	WGM2012_Bouguer Mean	2 min	Balmino et al 2014
r.bulk	Bulk density of the fine earth fraction (kg/m^3^)	250 m	Hengl et al 2021
r.cec	Cation Exchange Capacity (cmol(+)/kg)	250 m	Hengl et al 2021
r.clay	Clay content (%)	250 m	Hengl et al 2021
r.distance	Distance to the coast (km)	30-arc sec	https://oceancolor.gsfc.nasa.gov/resources/docs/distfromcoast/
r.dust	Multi-models average of dust deposition (g/m^2^/yr)	1º x 1º	Chien et al 2016
r.elevation	Hole-filled Digital Elevation Model	90 m	Jarvis et al 2008
r.fert	Global fertilization	30-arc sec	Potter et al 2010
r.map	Mean annual precipitation (mm/yr)	30-arc sec	Harris et al 2020
r.mat	Mean annual temperature (ºC)	30-arc sec	Harris et al 2020
r.maxage_geol	GLiM age attribute – maximum (Myrs)	1 km	Hartmann and Moosdorf, 2012; Bataille et al 2020
r.meanage_geol	GLiM age attribute – mean (Myrs)	1 km	Hartmann and Moosdorf, 2012; Bataille et al 2020
r.minage_geol	GLiM age attribute – minimum (Myrs)	1 km	Hartmann and Moosdorf, 2012; Bataille et al 2020
r.pet	Global Potential Evapo-Transpiration (mm day^-1^)	30-arc sec	Trabucco and Zomer 2019
r.ph	Soil pH in H^2^O solution (x10)	250 m	Hengl et al 2021
r.salt	Simulation of sea salt aerosol deposition (g/m^2^/year)	1º x 1º	Chien et al 2016
r.sr	Strontium isoscape	1 km	Bataille et al 2020
r.ssa	Multi-models average sea salt wet deposition + dry deposition (kg/ha/yr)	1º x 1º	Vet et al 2014
r.ssaw	Multi-models average sea salt wet deposition (kg/ha/yr)	1º x 1º	Vet et al 2014

The coordinates of each archaeological site were used to extract the local values from each raster and to generate a regression matrix. Random forest (RF) was used to develop a predictive model to generate a δ^34^S isoscape for Iberia. RF is a widely used machine learning algorithm because of its high predictive performance, particularly when handling non-linear and complex relationships between dependent and independent variables. First, RF generates a set of decision trees by subsetting the training data through bootstrapping. A set of predictor variables is considered at each split, allowing the method to create multiple decision trees on different data subsamples. This approach employs sampling with replacement to prevent overfitting. The overall prediction is then aggregated by combining the results of these decision trees to forecast the mean value of the response variable. In this study, we optimised the models using the Root Mean Squared Error (RMSE) as the primary metric. Following previous studies [2], a 10-fold repeated cross-validation approach with five repetitions was implemented, using 80% of the data for training in each iteration. Therefore, the remaining 20% of the sample (i.e., out-of-bag) was used to test the model predictions. To enhance model performance while minimising the number of predictors, we used the Variable Selection Under Random Forest (VSURF) R package [58]. This package aids in eliminating irrelevant and redundant variables through a three-step process. First, uncorrelated variables with δ^34^S are eliminated from the dataset; second, all variables related to the δ^34^S are selected; lastly, the predictive variables selection is refined by eliminating redundancy (i.e., multicollinearity).

We used two metrics to assess the importance of each selected predictive variable. The Mean Decrease Accuracy (%IncMSE) is the mean decrease in accuracy over an out-of-bag sample when a given variable is excluded from the models. Thus, %IncMSE reflects how much the model accuracy decreases when we exclude one of the selected variables. On the other hand, the Mean Decrease Gini (IncNodePurity) uses the Gini Impurity Index to calculate the importance of each independent variable based on the splits in trees. Therefore, the most critical predictors will correspond to those with the highest %IncMSE and IncNodePurity values. We used partial dependence plots to further explore the association between each predictor variable and the predicted δ^34^S. Finally, a δ^34^S isoscape was generated using the Random Forest regression model that demonstrated the best performance. To assess the spatial distribution of prediction uncertainty, we followed the pseudo-standard deviation procedure [59]. Thus, we predicted the 17^th^ and 83^rd^ percentiles of the response distribution. These quantiles represent the lower and upper bounds of the model’s expected uncertainty range in each cell. Then, we calculated the pseudo standard deviation map by taking half the difference between the upper and lower quantiles. Finally, the potential provenance of the individuals identified as migrants was estimated using continuous-surface isotope-based geographic assignment via the *assignR* package [60]. The R scripts and associated data to replicate the isoscape are available in this link: osf.io/zmg9d.

## 4. Results

### 4.1. Stable isotopes

**Fig 2:**
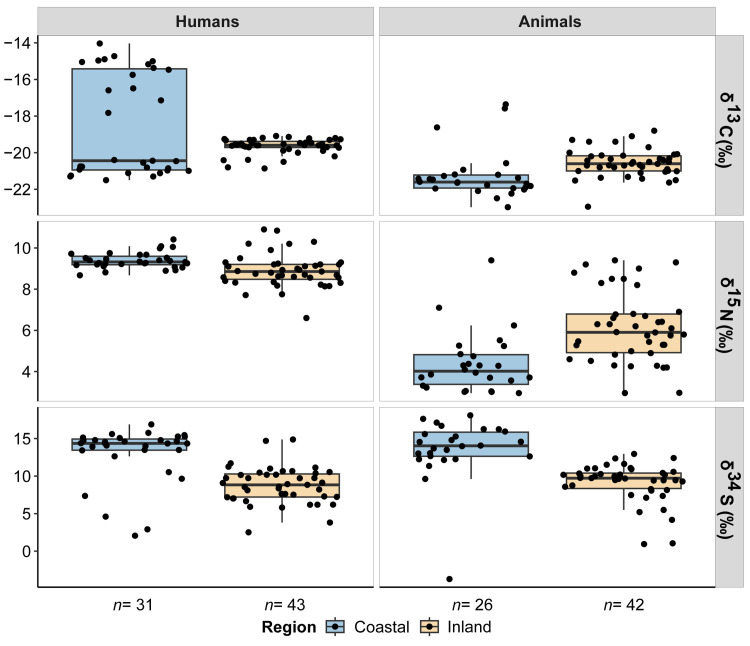
Boxplots of the δ^13^C, δ^15^N and δ^34^S stable isotope values of humans and animals from coastal and inland burial sites. Humans are represented on the left, and animals are represented on the right. Blue boxplots refer to coastal individuals and orange boxplots to inland individuals.

**Fig 3:**
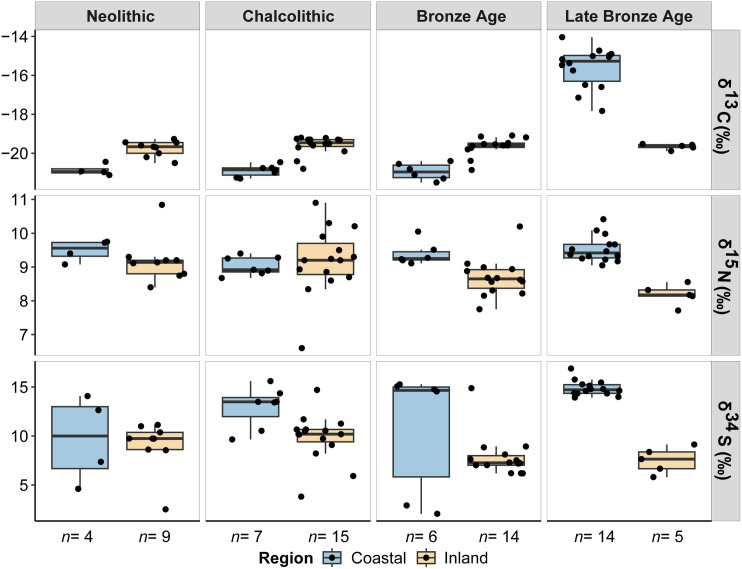
Boxplots of the human δ^13^C, δ^15^N and δ^34^S stable isotope values from coastal and inland burial sites across chronocultural periods. Blue boxplots refer to coastal individuals and orange boxplots to inland individuals.

The stable isotope values for human and animal remains are plotted in Figs 2 and 3 and reported in S1 Table. Mean, median, maximum, minimum and standard deviation values are reported by each isotope in S2-S4 Tables, while statistical correlations and comparisons are found in S5 and S6 Tables. An extended version of the stable isotope results by site at the species level can be seen in the S1 Text. Collagen extraction was successfully undertaken in all the samples with %Col > 1. All quality control standards indicate generally good collagen preservation, except for six samples whose C:S and N:S were slightly below the established margins. For those reasons, these samples were excluded from the isoscape mapping. Replication was typically less than 0.1‰, demonstrating high analytical precision. The δ^13^C and δ^15^N values will be discussed initially as they provide important context from which to interpret the δ^34^S isotope values. We take into account the sample size of coastal and inland regions, which provides unevenness of the sample (Fisher’s exact test *p* = 0.03). Despite the differences in size between regions at certain periods, we observed a significant difference in both humans and animals, which remains homogeneous across all periods.

**Fig 4:**
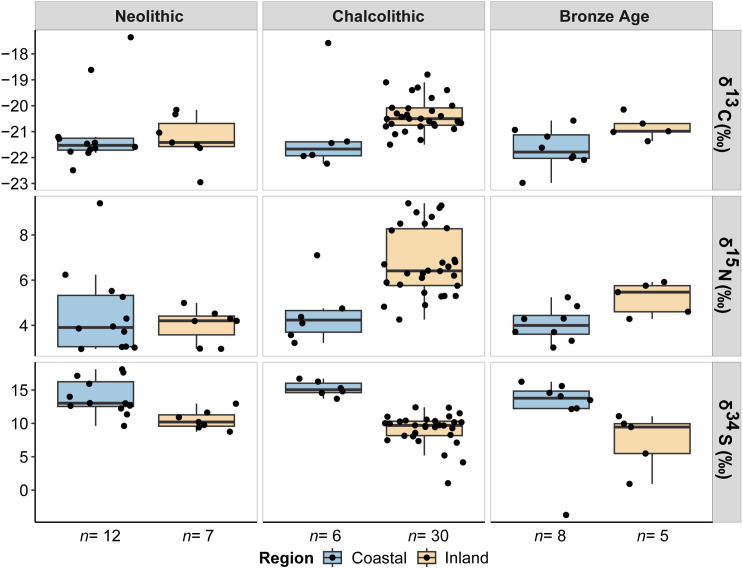
Boxplots of the animal δ^13^C, δ^15^N and δ^34^S stable isotope values from coastal and inland burial sites across chronocultural periods. Blue boxplots refer to coastal individuals and orange boxplots to inland individuals.

Humans from coastal sites (*n* = 31) had δ^13^C values ranging between −21.5‰ and −14.0‰ (*X̅ = *−18.5‰), slightly higher than humans from inland sites (*n* = 43), which showed δ^13^C values ranging between −20.9‰ and −19.1‰ (*X̅ =* −19.7‰) (Fig 2 and S2 Table). However, the cluster of coastal humans shows two groups according to their δ^13^C values, one with higher values and another with lower ones than inland humans, which suggests the existence of different regimes of plant consumption in the sample (C_3_ vs C_4_ plant consumers). If we consider their median value, carbon coastal humans would be lower than carbon inland humans. Animals from coastal sites (*n* = 26) displayed lower δ^13^C values ranging between −23.0‰ and −17.4‰ (*X̅ =* −21.2‰), than animals from inland sites (*n* = 42), which reported δ^13^C values ranging between −23.0‰ and −18.8‰ (*X̅ =* −20.6‰). If filtered by cultural periods (Fig 3 and S3 Table), the δ^13^C values of inland humans are higher by around 1‰ compared to coastal humans. This pattern is replicated through the Neolithic (*X̅ =* −19.8‰ vs −20.9‰), Chalcolithic (*X̅ =* −19.6‰ vs −20.9‰) and Bronze Age (*X̅ =* −19.7‰ vs −20.9‰). During the Late Bronze Age, the trend shifts (*X̅ =* −19.6‰ vs −15.6‰), indicating a significantly different carbon intake in coastal humans, likely due to the inclusion of C_4_ plants in their diet. In the case of animals, this pattern is equally identified in the Chalcolithic (*X̅ =* −20.4‰ vs −21.1‰) and Bronze Age (*X̅ =* −20.8‰ vs −21.7‰) (Fig 4 and S4 Table). However, the coastal animals (*X̅ =* −21.0‰) are slightly elevated in comparison to the inland animals (*X̅ =* −21.3‰) during the Neolithic because of two omnivorous animals with higher carbon values. Therefore, the coastal-inland differences observed in human samples are consistent with differences observed in other mammals.

With regard to nitrogen, the δ^15^N values of coastal humans range between 8.7‰ and 10.4‰ (*X̅ =* 9.4‰), being slightly higher than the inland humans, whose δ^15^N values vary between 6.6‰ and 10.9‰ (*X̅ =* 8.9‰). In contrast, the inter-individual variability in the δ^15^N values of inland animals is greater, ranging between 3.0‰ and 9.4‰ (*X̅ =* 6.1‰), while the δ^15^N values of coastal animals range between 3.0‰ and 9.4‰ (*X̅ =* 4.4‰) (Fig 2 and S2 Table). Although the nitrogen isotope results are very homogenous between regions, higher nitrogen isotope values can be seen across the Neolithic (*X̅ =* 9.5‰ vs 9.2‰), Bronze Age (*X̅ =* 9.4‰ vs 8.7‰) and Late Bronze Age (*X̅ =* 9.5‰ vs 8.2‰) for the coastal humans, excepting during the Chalcolithic, when a slightly higher mean value is reported in inland individuals (*X̅ =* 9.0‰ vs 9.2‰) (Fig 3 and S3 Table). Conversely, the δ^15^N values of inland animals are much higher than the coastal animals during the Chalcolithic (*X̅ =* 6.8‰ vs 4.5‰) and Bronze Age (*X̅ =* 5.2‰ vs 4.1‰), except during the Neolithic (*X̅ =* 4.0‰ vs 4.5‰), probably due to a single canid with an elevated δ^15^N value from consuming meat (Fig 4 and S4 Table). In fact, if we consider the median value, the nitrogen isotope values of inland animals would be equally higher than those of coastal ones. These differences may be due to different economic specialization between regions (i.e., use of manure in agricultural practices).

Coastal humans had slightly higher δ^34^S isotope values, ranging between 2.1‰ and 16.9‰ (*X̅ =* 13.0‰), than inland humans, which reported δ^34^S isotope values between 2.5‰ and 14.9‰ (*X̅ =* 8.8‰). The difference in sulfur isotopic composition between coastal and inland sites would be even greater, removing the six individuals of the coastal human cluster, which have lower δ^34^S isotope values than the main group. Besides, this trend is also corroborated by the faunal specimens of both regions despite the existence of a specimen with a negative sulfur isotope value. The δ^34^S isotope values of coastal animals ranged between −3.7‰ and 18.1‰ (*X̅ =* 13.6‰), while the δ^34^S isotope values of inland animals vary between 0.9‰ and 13‰ (*X̅ =* 9.1‰) (Fig 2 and S2 Table). Thus, both in humans and in animals, the sulfur isotope values of coastal sites are higher than those seen in inland sites. This elevation in δ^34^S isotope values is also noticeable for humans during the Chalcolithic (*X̅ =* 12.9‰ vs 9.8‰), Bronze Age (*X̅ =* 10.8‰ vs 7.9‰) and Late Bronze Age (*X̅ =* 14.9‰ vs 7.5‰), but also for animals during the Neolithic (*X̅ =* 13.9‰ vs 10.5‰), Chalcolithic (*X̅ =* 15.2‰ vs 9.1‰) and Bronze Age (*X̅ =* 11.8‰ vs 7.4‰), with the sulfur isotope values of coastal individuals being consistently higher than that of inland individuals in all periods. However, this pattern is smoothed in coastal humans during the Neolithic (*X̅ =* 9.7‰ vs 9‰), probably due to the small sample size and the existence of two individuals with lower sulfur isotope results (Figs 3 and 4, S3 and S4 Tables).

Statistical tests have not detected numerous correlations between the different isotope systems (S5 Table). There are no significant correlations between δ^13^C and δ^15^N or between δ^13^C and δ^34^S isotope values in either coastal or inland humans, which would be expected if it was a consequence of climatic factors or dietary preferences, respectively. Instead, there is a weak positive correlation between the δ^15^N and δ^34^S isotope values of coastal humans (*rho* = 0.37, *p* = 0.04), which is not seen in inland humans. This correlation persists when the data are divided into cultural periods, being significant during the Chalcolithic (*rho* = 0.89, *p* = 0.01) and Late Bronze Age phases (*rho* = 0.67, *p* = 0.01). This correlation would be expected if the sulfur and the nitrogen isozones were interconnected. A positive correlation is observed between animal δ^13^C and δ^15^N values from inland sites (*rho* = 0.45, *p* = 0.00), but this correlation is only identified in the Chalcolithic (*rho* = 0.36, *p* = 0.05) when filtered by cultural periods (S5 Table).

Regarding statistical comparisons, the δ^13^C, δ^15^N and δ^34^S isotope values of human and animal populations from coastal sites were statistically significantly different from those in the inland locations (*p* < 0.001) (S6 Table). This trend is seen in all isotopes except in the δ^13^C (*p* = 0.61), where no statistically significant differences were recorded between coastal and inland human groups, which is likely a product of there being two distinctive groups within the coastal humans as previously mentioned. This is noticeable when comparing the δ^13^C values of humans from coastal and inland sites, which are significantly different in all cultural periods (*p* = 0.00–0.01) (S6 Table). Regarding δ^15^N values, different trends can be observed. During the Neolithic, there were no significant differences between the coastal and inland populations of humans (*p* = 0.19) and animals (*p* = 0.90), while in the Chalcolithic, there were solely identified differences in the animals (*p* < 0.001). Finally, human and animal populations from coastal sites during the Bronze Age were statistically significantly different from those in the inland locations (*p* < 0.001 and 0.03, respectively). Similarly, there are statistically significant differences for δ^34^S isotope values between coastal and inland human and animal populations across cultural periods (*p* = 0.00–0.03), except in the case of the Bronze Age humans (*p* = 0.35) due to the presence of two individuals with lower sulfur isotope values in the coastal cluster (S6 Table).

### 4.2. Sulfur isoscape model

To further understand the trends in the δ^34^S isotope values from the human and animal remains studied here, a sulfur isoscape model was generated. The dataset comprises 554 δ^34^S isotope values measured on bone collagen from humans and animals from 41 site locations across Iberia, of which 161 were excluded after screening. In total, 393 sulfur isotope values were used to build the model. The δ^34^S isotope values are not normally distributed (Shapiro Wilk Test, *p* < 0.001) and ranged from 3.8‰ to 18.1‰ (*X̅ =* 12.2‰).

**Fig 5:**
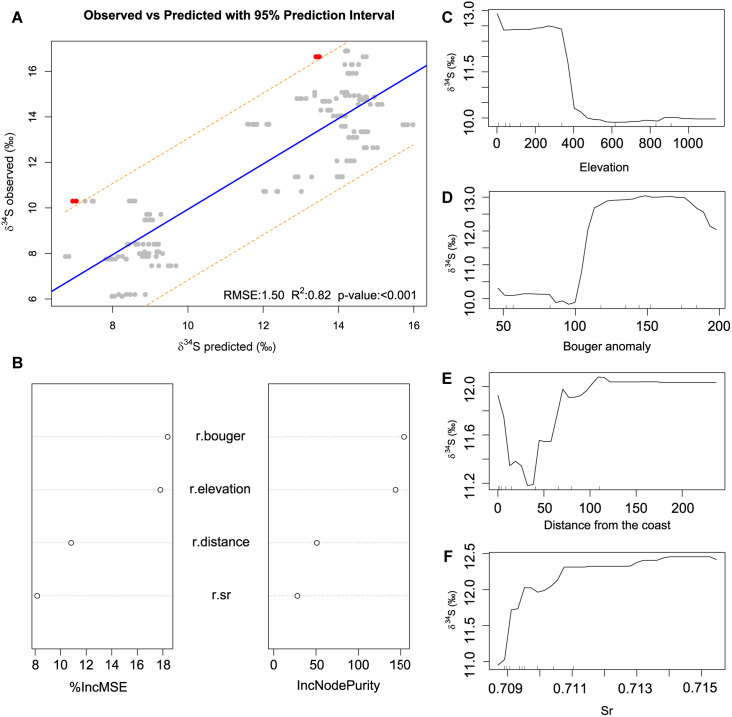
n-fold cross-validation and predictor for the δ^34^S random forest regression model: A) Observed and predicted δ^34^S isotope values. Root Mean Square Error (RMSE), correlation coefficient (R^2^) and *p*-value of the predictive model obtained with random forest. B) Mean Decrease Accuracy (%IncMSE) and Mean Decrease Gini (IncNodePurity) showing the importance of each variable. Higher importance values indicate more weight as predictors. C-F) Partial dependence plot depicting the association between each independent variable and the predicted δ^34^S isotope values.

The RF regression model produced a sulfur isoscape model, generating spatial solid patterns associated with physical and geological variables (S7 Table). After VSURF selection and considering the %IncMSE and IncNodePurity values, four variables, including elevation above the sea level (r.elevation), Bouguer anomaly (r.bouger), distance from the coast (r.distance), and strontium content of the soil (r.sr) were determined to be the dominant predictors of the δ^34^S isotope values (Fig 5B). Based on the dependence plots, elevation above the sea level exhibits a negative correlation with δ^34^S isotope values. Similarly, distance from the coast negatively correlates with δ^34^S isotope values since lower sulfur signatures are related to places further from the coast (Fig 5E-F). Conversely, the Bouguer anomaly and strontium isotope values increase is associated with higher δ^34^S isotope values. These correlations highlight the complexity of δ^34^S isotope values, which are non-linearly influenced by different variables (Fig 5C-F).

**Fig 6:**
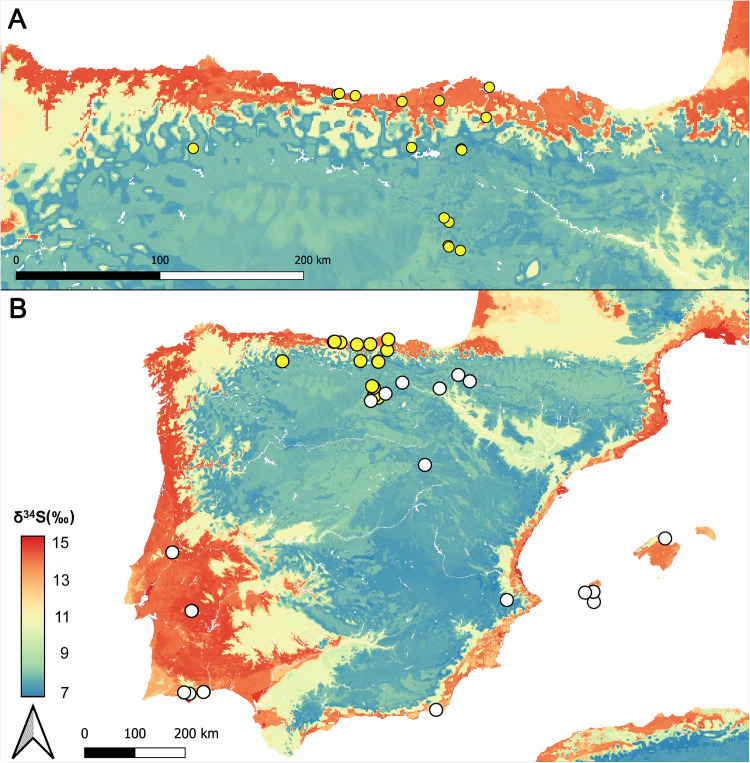
Sulfur isoscape model reconstruction across Iberia: A) Spatial distribution of the sulfur isotope composition (δ^34^S) across northern Iberia. B) Spatial distribution of the sulfur isotope composition (δ^34^S) across the whole of Iberia. Yellow dots indicate the sites explicitly analysed in this study, and white dots refer to a compilation of animal and human bone collagen from Holocene times.

**Fig 7:**
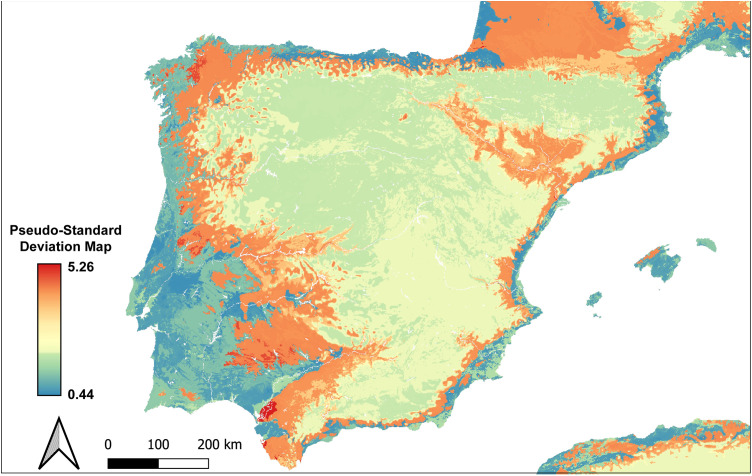
Pseudo Standard Deviation map of the predictive δ^34^S isotope values. Higher values indicate more uncertainty of the predictions.

The obtained predictive model explains two-thirds of the variance (R^2^ = 0.82), and the Root Mean Square Error (RMSE) is 1.50, which reflects a good model performance. Only two sites, Kaite (Burgos) and Ca Na Costa (Formentera), exhibit a poor model fit, as they fall slightly outside the 95% confidence interval. However, both sites reflected a homogeneous trend in sulfur isotope values of humans and animals, showed similar diets to their neighbouring sites, and only two of the five repetitions of the 10-fold repeated cross-validation approach were considered as outliers. For these reasons, both sites can be interpreted as reliable predictions. Thus, there is a positive correlation between the observed and predicted δ^34^S isotope values (Fig 5A). According to this model, the highest δ^34^S isotope values are found in the coastal areas of the Atlantic façade, including the Cantabrian Region and Southwest Iberia (Guadalquivir Valley, Spanish Extremadura and South of Portugal) (Figs 6A and 6B). In the same way, the Mediterranean coasts of Iberia have moderately high δ^34^S isotope values influenced by a softer marine sea salt deposition. In contrast, the lowest values are found in the inner areas of Iberia (Fig 6B). Although this isoscape model constitutes a first, important, step forward, caution is needed when interpreting these spatial patterns since the pseudo-standard deviation map show differences between 2 (i.e., North and South plateau) and 5 (i.e., Ebro valley) in the predictions ranges of the inner areas. In contrast, in the coastal areas and the southwest of Iberia, this difference is about 1 (Fig 7). This spatial distribution of quantile ranges suggests that the uncertainty in the predictions is substantially lower in coastal than in inner areas of Iberia (Fig 7). This approach shows limited power when predicting sulfur in regions without training data. Precisely, our sites do not cover all the variability of the rasters that exists in Iberia, and consequently, major variations could be missing in Inner Iberia, which could cause changes in the predicted minimum sulfur isotope values in case of having more sites in these areas.

## 5. Discussion

### 5.1. Insights on the diet of Late Prehistory in northern Iberia based on δ^13^C and δ^15^N values

Most of the human δ^13^C values are consistent with a terrestrial C_3_ ecosystem during the Holocene in North Iberia [61] (Fig 2). The higher δ^13^C isotope values seen in humans and animals from inland sites can be explained by baseline environmental factors (i.e., lack of canopy effect, low precipitation, soil aridity, high altitude, absence of salinity) rather than by dietary differences [62–67] since a notable difference has been observed between southern and northern European regions [68]. Regarding possible plant resources that would have been available, wheat and barley were the most common cereals during Late Prehistory in Iberia [69]. Pulses, such as peas, lentils and fava beans, were also cultivated beginning in the Neolithic [70]. The only site that breaks this pattern is the Late Bronze Age coastal site of El Espinoso (Asturias) (Fig 2 and S2 Fig), where higher δ^13^C values in combination with the identification of polyhedral starch grains within dental plaque, indicated the consumption of C_4_ plants, mainly millets, in the diet of these individuals [71]. However, this dietary behaviour does not appear to have been common in all north-Iberian regions, as other contemporary individuals such as the inland individuals of La Quebrantada and Trulla caves (Burgos), and the Cantabrian megalithic tomb of Ondarre (Guipúzcoa) [72] do not display elevated δ^13^C isotope values derived from eating millets. Thus, the δ^13^C isotope evidence does not support the consumption of C_4_ plants in Iberia before the Late Bronze Age. So, if these cereals were consumed among the preceding communities, they were not in sufficient quantities to be recorded in the bone collagen, indicating infrequent or occasional consumption of these plants. In fact, the first carpological and anthracological evidence of millet cultivation in Iberia dates to the Middle Bronze Age, although millets were not systematically exploited until the Late Bronze Age and Iron Age [73,74].

In terms of δ^15^N values, humans showed values 3–5‰ higher than the contemporary herbivores, which is the standard trophic level offset between consumers and prey [75] and suggests that the Holocene groups from both slopes of the mountain range were eating a mixed C_3_ diet that included animal protein (meats and dairy products) (Fig 2). This is also supported by the molecular and isotope analysis of lipids on pottery sherds [76]. According to the archaeozoological evidence, caprine, cattle, and pigs constitute the most probable animal protein source consumed by these farming groups [77,78]. Protein consumption remains stable through time for humans, being slightly higher in coastal than among inland humans, likely due to greater salinity in coastal locations [67]. However, this may also indicate the consumption of different quantities of animal protein within both communities, with relevant implications for the understanding of the subsistence strategies (i.e., pastoral vs agricultural economies) [79]. Moreover, the high δ^15^N values of inland animals compared to coastal animals could be indicating the use of livestock manure to enhance crop yields [80] at southern locations of the Cantabrian mountain range, as manuring significantly raises the δ^15^N values [81].

The δ^13^C and δ^15^N values of the human remains from the sites analysed here are also not indicative of marine or freshwater resource consumption, even in the coastal individuals, despite their relative proximity to the sea, which coincides with the absence of archaeological evidence of marine resource consumption among Late Neolithic/Chalcolithic/Bronze Age communities (shell or fish remains) [82,83]. The intake of marine foods into the human diet would reflect higher δ^13^C and δ^15^N isotope values, suggesting sporadic or even no consumption throughout the chronological sequence. Other palaeodietary studies in northern Iberia show similar results for their δ^13^C and δ^15^N isotope values [16,72,84–93]. In summary, the diet of these farming populations was relatively homogenous until the Late Bronze Age (Fig 3), both in the cereals grown and in the animal protein consumed, which is in line with the trend seen across Iberia [61].

### 5.2. Coastal/inland mobility patterns using δ^34^S isotope values

The elevated δ^34^S isotope values observed within the coastal populations are unlikely to have been produced by the consumption of freshwater and marine resources, which can cause elevated δ^34^S isotope values [1], as the δ^13^C and δ^13^N isotope results indicated that this was not an important dietary component. Notably, the baseline herbivorous faunal specimens from coastal sites had similarly high δ^34^S isotope values to humans (Fig 2), suggesting that their δ^34^S isotope values were more likely to be associated with environmental factors rather than dietary behaviour. The sea spray effect has a significant impact on coastal environments reaching more than 30 km inland, increasing the sulfur isotope values in consumers and prey [6], which suggests that the majority of the humans and animals found buried in coastal locations would have predominantly lived near the coast during their lifetimes. Notably, inland individuals reported similar δ^13^C and δ^15^N ranges to the coastal populations but had lower δ^34^S isotope values in humans and animals studied (Fig 2), demonstrating that they followed similar diets, but the marine spray effect did not influence them. Thus, this research confirms that sulfur can be used to identify population movements due to the possibility of distinguishing between coastal and inland sulfur isotope signals, as suggested by initial studies in the Cantabrian Region [16,36]. However, the δ^34^S isotopic signal in bone collagen reflects the average diet (and, therefore, the exploited landscape) during the last years of life [26], and this is influenced by the remodelling rate of the sampled bones [94], attenuating any possible signal of seasonal movements between coastal and/or inland isozones, and rendering invisible those to isozones with similar isotopic values.

The intra-regional analysis showed some distinctive trends, indicating that there was some movement of people during Late Prehistory between coastal and inland locations. Within the coastal sites of the Cantabrian Region, six of the 31 humans (19%) have reported lower δ^34^S isotope values than the main group of human specimens (n = 25), which showed higher δ^34^S isotope values, consistent with the baseline fauna from coastal region (n = 26) (Figs 2 and 3). The higher δ^34^S isotope values of the main cluster of humans and animals indicate that they all live in the same location. It is likely that they predominantly lived near the coast, with sulfur isotope values affected by the sea spray effect, which typically produces elevated δ^34^S isotope values [32]. In contrast, the lower δ^34^S isotope values of the remaining six individuals suggest that they spent at least a large proportion of their lifetime living in an area that is represented by lower baseline δ^34^S isotope values, which could be reflecting an isotope signal typical of inland territories, as δ^34^S isotope values decrease with distance from the coast [1]. For these reasons, individuals within the main group can be considered local inhabitants of the Cantabrian coastal zone, while the remaining six are likely migrants who arrived in this region later in their lives, where they were then buried. The likely location of these people’s origin could be further south, on the high Ebro valley or the North Castilian Plateau, a high inland region with a dry continental climate of hot summers and cold winters. The individuals identified as migrants were buried at four caves: El Hondón (Asturias), Los Avellanos I, Los Avellanos II and El Abrigo de la Castañera (Cantabria). Three individuals from Los Avellanos I (n = 2) and II (n = 1), dated in the Late Neolithic and Chalcolithic periods, were initially proposed to be non-locals by falling outside the sulfur isotope range of the main group of humans and animals buried at both sites (S11 Fig in S1 Text) [16]. Another Late Neolithic individual with low sulfur isotope values within the main cluster of individuals buried in this cave was documented in El Hondón (S4 Fig in S1 Text). Finally, two Early Bronze Age individuals with even lower δ^34^S isotope values that indicate a possible non-local origin were recovered in El Abrigo de la Castañera (S7 Fig in S1 Text). These data prove that there was movement of people from inland locations to the coast from the Neolithic to the Bronze Age.

Of the individuals buried within inland sites, only two of 43 individuals (5%) analysed appeared to have been non-local to the region, exhibiting elevated δ^34^S isotope values that are more consistent with having lived on the coast for a prolonged period of time (Figs 2 and 3). The bulk of specimens (41 humans and 42 animals) reported lower δ^34^S isotope values, which can be interpreted as being local people and animals from inland territories. One individual buried at the megalithic monument of Arroyal I (Burgos) showed clear evidence of non-local origin. This Chalcolithic individual had a higher δ^34^S value than the other people identified inside the dolmen (S14 Fig in S1 Text), suggesting the person lived in an area influenced by the sea spray effect before dying and was likely to be from the Cantabrian Region [36]. The other eight individuals buried there reported lower δ^34^S isotope values typical of an inland area, like the one surrounding the site, according to the faunal specimens sampled there. A further individual was found in a crevice after descending four levels of the Los Cinchos cave (Asturias), a cavity 1,870 meters above sea level located on the northern slope of the Cantabrian Cordillera in an inland area more than 60 km from the shore, provided more problematic evidence of mobility. This individual, dated to the Early Bronze Age, showed an elevated δ^34^S value (S21 Fig in S1 Text), indicating that this individual would have lived the last years of its life in an isozone with high baseline sulfur isotope values. Although higher sulfur isotope values can be found in diets heavily influenced by marine resources [8], the individual’s δ^13^C and δ^15^N isotope values do not support this hypothesis. It seems more plausible that this individual came from a coastal location influenced by the sea spray effect [32]. Biological sex estimation established that this individual was male, and skeletal analysis identified enthesopaties in their clavicles [95], which has been documented in prehistoric mining contexts [96], and the proximity of the contemporaneous copper mines of El Aramo [97] support the interpretation that this person may have moved to the high mountain inland area and entered the karst system’s depths in copper mining explorations. However, given the absence of a faunal isotope baseline from this site, we cannot rule out that evaporitic rocks, which also can cause high sulfur isotope values [31], are responsible for the elevated δ^34^S value. Overall, both individuals hint at sporadic human mobility from the Cantabrian Region to the Northern Plateau, highlighting that movements in the opposite direction may have been more common during Late Prehistory. Alternatively, three inland humans with sulfur isotope values even lower than those seen in typical inland sites were found in La Quebrantada (*n* = 1) and Trulla caves (*n* = 2) (S24 and S25 Figs in S1 Text) dated to the Late Neolithic and Chalcolithic, respectively. Both sites are located in the same municipality, and their sulfur isotope values are significantly different to the main group of humans and animals, suggesting that they belonged to another inland isozone lower in sulfur.

**Fig 8:**
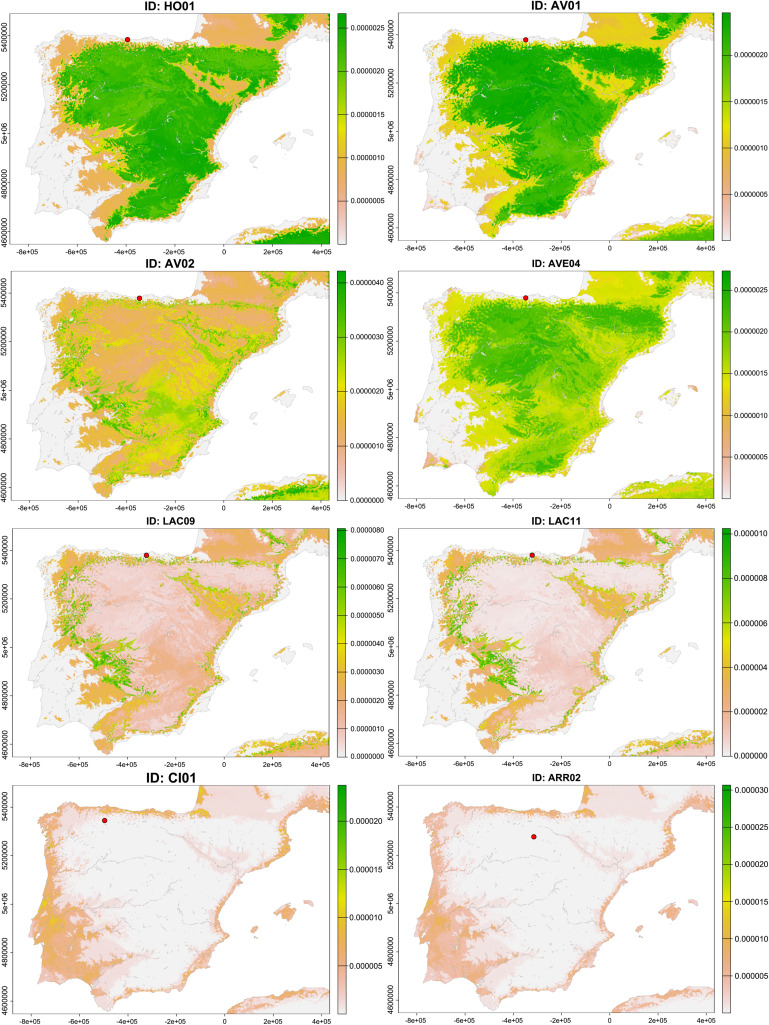
Probabilistic geographic assignments of the migrant individuals. Inland humans buried in coastal sites (HO01, AV01, AV02, LAC09, LAC11 and AVE04). Coastal humans buried in inland sites (CI01 and ARR02).

**Fig 9:**
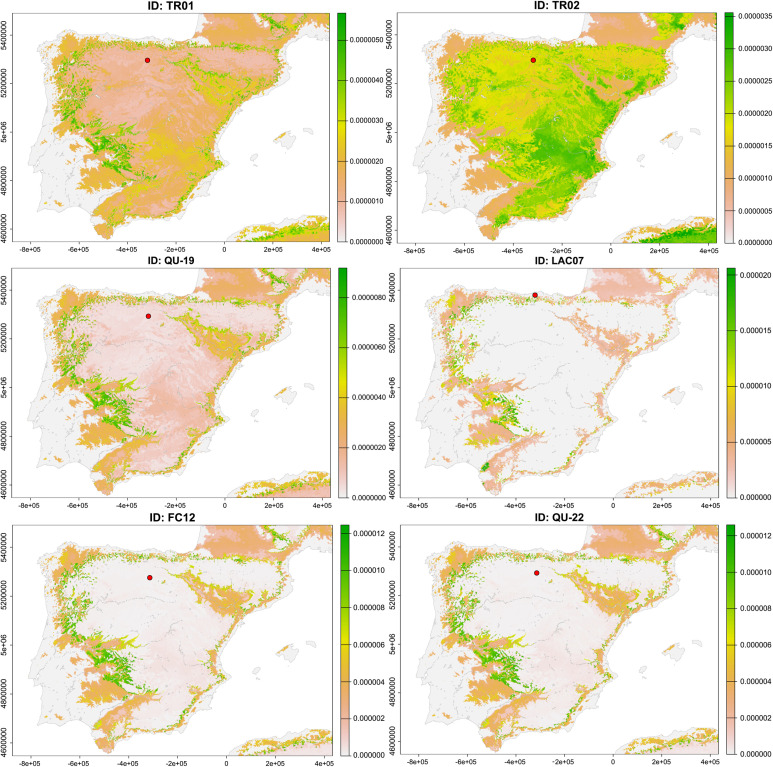
Probabilistic geographic assignments of the migrant individuals. Inland humans buried in other inland sites (TR01, TR02 and QU-19). Inland animals buried in coastal sites (LAC07). Inland animals buried in other inland sites (FC12 and QU-22).

After using continuous-surface isotope-based geographic methods for the migrants identified in this work, we attempted to calculate the most probable areas of origin of these individuals (Fig 8). In the case of the coastal individuals of El Hondón and Los Avellanos I and II, the predictions placed them in a vast inland area, confirming that they are non-local inhabitants of this coastal area. The closest and most probable regions of origin would be the North Plateau and the high Ebro valley. In contrast, the potential provenance of the coastal individuals from El Abrigo de la Castañera is much more spatially limited, revealing that they originally belonged to an inland region, but noticeably different and far away from the first (maybe Northwestern Iberia, the Ebro valley, or even the Pyrenees). In the case of the inland individuals of Los Cinchos and Arroyal I, both probabilistic geographic assignments clearly indicate that they come from a coastal area, and due to proximity, it seems clear that they come from the Cantabrian coast. On the other hand, the inland migrants from Quebrantada and Trulla were probably derived from a restricted inland area far from the burial sites (Fig 9).

Age and sex identification of the eight identified non-local individuals showed that they were predominantly adult males. One individual from Los Cinchos was estimated to be a late adolescent (16–18 years old). One adult from Los Avellanos II could not be sexed because of the highly fragmented nature of the skeletal remains, and one individual from El Abrigo de la Castañera was determined to be biologically female. Subsequently, DNA analyses of some individuals have confirmed the biological sex of four males from the sites of: El Hondón, El Abrigo de la Castañera, Arroyal I and Los Cinchos. Thus, these migrants can be interpreted as a result of the small-scale movement of people, which is compatible with segmentary-type societies.

Regarding animal mobility, the sulfur isotope values indicated that the majority of animals were consistent with having lived locally to the coastal and inland locations where they were found. One sheep/goat from El Abrigo de la Castañera coastal site had a negative δ^34^S value (S7 Fig in S1 Text), demonstrating that this sheep/goat would have spent a large part of its life in an area with even lower sulfur isotope baseline values than the inland individuals. Another two caprine specimens from Fuente Celada and La Quebrantada inland sites also showed very low sulfur isotope values compared to the main group of animals of these sites, such as the aforementioned humans from Quebrantada and Trulla, supporting a significant degree of mobility, at least, in these individuals (S18 and S24 Figs in S1 Text). This sulfur isozone with values lying close to zero, has not yet been characterised through isoscape mapping, but its anomalous signals suggest that it proceeded from a different inland geographical area than those identified so far. This sporadic animal movement could be easily explained as a result of livestock exchanges motivated by biological, social, or economic reasons [98–100]. Another hypothesis could be altitudinal sheep mobility, already identified through experimental [101] and archaeological [102] analyses of incremental δ^13^C and δ^18^O in dental bioapatite between the Ebro Valley and the Pyrenees since Neolithic times. Long-distance mobility of caprine flocks would imply the periodic displacement of livestock and people along routes that linked the highlands with lower altitudes, requiring a logistical and political infrastructure necessary to allow seasonal or annual livestock movements [102], which means that if it occurred, it must have occurred sporadically and on a small scale during Late Prehistory. However, more isotope data are needed in different highland and lowland areas of northern Iberia to verify this hypothesis.

To determine the inland zone of provenance for the caprine individuals from El Abrigo de la Castañera, Fuente Celada, and Quebrantada, probabilistic geographic assignment confirms that they originated from areas far from their burial sites and restricts this area to specific regions of Iberia (Fig 9). In particular, the most plausible areas from a geographical and archaeological perspective would be the Pyrenees, the Ebro valley and Northwestern Iberia. However, this cannot be confirmed without new sampling in these areas.

### 5.3. Sulfur isoscape of northern Iberia

The predictor variable that most explains the model was elevation. Lower δ^34^S isotope values are observed in higher altitudes, and this negative relationship is particularly noticeable for altitudes surpassing 500 meters (Fig 5). The negative correlation between the sulfur isotope values and elevation is probably due to the preferential uplift and exposure of older radiogenic units during orogenies [57] or due to greater erosion of bedrock sulfide and sulphate at elevation. Another high predictor variable that, in turn, is influenced by elevation was the Bouguer anomaly, which is defined by the difference between the observed gravitational attraction at a specific location and the theoretical gravitational attraction adjusted for the effects of geology and topography [103]. Positive Bouguer anomalies (>100mGal) are associated with higher δ^34^S isotope values, whereas low and negative Bouguer anomalies are associated with lower δ^34^S signatures [2]. Positive Bouguer anomalies across Iberia are mainly located along the coastlines, such as the Cantabrian Region, where the altitude is low in the coastal platform and valleys. Conversely, negative Bouguer anomalies are found in elevated mountain ranges of compressional orogens, such as the steep Cantabrian chain or in inner highland regions of Iberia. Similarly, as observed in other studies, the δ^34^S isotope values were also negatively correlated with distance to the coast [104], the third variable that most drives the model. Considering the sample distribution, it is very likely that a fortuitous effect of elevation, with low-lying coastal areas having high δ³⁴S and higher elevation inland areas having lower δ³⁴S, is driving the model. The same is likely true for the Bouguer anomaly, which is strongly correlated with elevation. On the other hand, the distance to the coast is likely related to the predominance of northwestern marine aerosols arriving at the Cantabrian coast. Thus, food systems near the coast receive heavier marine sulfates. However, the relationship between sea salt aerosol deposition and distance to the coast is not linear due to topographical factors [2], as seen in areas of abrupt relief like the Cantabrian Region. This would explain why the model does not select that variable in a region with a high sea spray effect. Although marine sulfur can be carried over long distances [1], the singularity of the Cantabrian Region, with a steep mountain chain of about 2,000 meters above sea level lying only 30–50 kilometres from the Holocene shore, quickly prevents the southward spread of sea spray into the high inland locations. Thus, at distances greater than 30 km from the ocean, bone collagen δ^34^S isotope values decrease because the living organisms are not influenced by sea salt deposition rates, and other geological factors take over to control δ^34^S isotope values. In this regard, the only geological variable considered predictable by the model was the strontium content of the soil. This is not surprising because sulfur isotope values are primarily derived from mineral weathering of underlying lithology and are spatially variable according to lithological and soil factors [31], at least in inland regions. Additionally, δ^34^S isotope values are positively correlated to strontium isotope values. In this sense, the interior of the Iberian Peninsula contains older igneous or metamorphic rocks that are richer in reduced sulfides, which tend to report low sulfur and strontium isotope values [2,57]. In contrast, our model has not identified negative sulfur isotope values linked to wetlands with high levels of isotopically light sulfides derived from the anaerobic conditions in which the soils were formed [13]. Nor does it predict high sulfur isotope values in places with evaporitic rocks rich in marine sulfides [29]. Thus, elevation, Bouguer and distance to the coast are probably interconnected with each other and represent only one variable that explains most of our sulfur isoscape. However, especially in inland areas, a geological influence can be suggested due to strontium as a predictor variable.

### 5.4. Archaeological evidence of the circulation of materials, ideas and people on a supraregional scale

Archaeological evidence of contact between coastal and inland inhabitants of northern Iberia during the Late Prehistory is still sparse [16]. Conversely, new multidisciplinary studies suggest supraregional circuits of exchanges between the Cantabrian Region, high Ebro Valley and North Castilian Plateau, regions in which farming communities used many of the same raw materials, artefacts, personal ornaments or prestige items [105].

Some seashells probably used as ornaments have been identified in different burial sites of the Northern Plateau [106]. In the inland pits field site of El Hornazo (Burgos), a marine bivalve shell (*Ruditapes decussatus*) was documented in Pit 141 [107], suggesting that it was collected in the Cantabrian Sea. Likewise, three *Trivia europea* necklace beads from La Velilla (Palencia) [108] and Las Arnillas (Burgos) [109] have been found, this being a species native to the Atlantic. More controversial are the ornaments on tubular, Dentalium-type shells due to their abundant presence on the Iberian Mediterranean coast during Late Prehistory, although their possible Cantabrian origin cannot be completely ruled out. La Peña de la Abuela and La Tarayuela megalithic tombs (Soria) provided the finds of 154 and 48 beads of the species *Dentalium sexangulum*, *Antalis* *cf.* *vulgaris*, *Antalis inaequicostatum* and *Antalis* sp. [110]. Similarly, these shells were also documented in El Miradero, Los Zumacales (Valladolid) [111], and Fuentepecina II (Burgos) [109]. Most likely is the Cantabrian provenance of the perforated shell of *Glycimeris glycimeris* also found in La Tarayuela [112] and El Cubillejo de Lara (Burgos) [113]. This species can live in the Atlantic, as well as in the Mediterranean. Many amber beads have also been identified in megalithic monuments, such as La Velilla or Las Arnillas [108,109]. Despite the presumed foreign origin of many ambers in Iberia, we cannot ignore the abundance of Cretaceous amber outcrops in the Cantabrian mountain range, already exploited in the Palaeolithic and also in the Late Prehistory of this region [114]. Another type of ornaments present in more than thirty Northern Plateau burial sites, such as the variscite, probably reflects a large-scale exchange across Iberia [115]. Two main areas of variscite exploitation have been discovered in the NE Mediterranean (Can Tintorer mines, Barcelona) and in the Southwest (Pico Centeno mines, Huelva). However, variscite mines have also been discovered recently in the Aliste region (Zamora) [116], suggesting a closer catchment area than previously thought and placing them as candidates for the variscite materials found in the Cantabrian Region. Similarly, other minerals such as talc or lignite, widely used to make necklace beads during Late Prehistory and documented on a large scale in numerous funerary sites of the Northern Plateau, can be found widespread in the Cantabrian Mountain range [105].

Another line of evidence is the typological similarities between some Cantabrian ceramics decorations and Northern Plateau motifs, aligning with our sulfur results. Two vessels from Los Avellanos I have stylistic parallels with ceramics from the sites of El Púlpito, El Hornazo and Fuente Celada in the plain of the Arlanzón River valley of Burgos [117]. Likewise, another vessel from Las Lapas (Cantabria) shows a zigzag decoration typical of the carinated pots of the Protocogotas horizon [118], geographically extended through the eastern area of the Northern Plateau and the Alava Plain in the trans-Cordillera Basque Country. Besides, the bowl that incorporates a mamelon from Santimamiñe (Vizcaya) contains a recurring shape in the contexts of the Middle Bronze Age in the Meseta and Upper Ebro Valley areas [118]. Other pottery can be related to Cogotas I culture, such as the sherds decorated by the boquique technique documented in El Mirón or El Linar (Cantabria) [119]. Similarly, the existence, although sparse, of Bell Beaker elements throughout the Cantabrian coast contrasts with the rich record existing in the Northern Plateau, which stands as a possible source of origin for these materials on a south-north axis [120]. Most of the clays used in the manufacture of pottery are compatible with the local sources found in the surroundings of the burial sites, suggesting that these technotypological similarities are related to possible movements of ideas and/or people [117,118]. In this sense, a female exogamy through marriage was proposed to explain the contacts between the Cantabrian Region, the upper Ebro Valley and even southwestern France during the Early Bronze Age [114]. Recent DNA-based analyses of kinship practices have confirmed this behaviour from the Neolithic onwards in Britain [121], France [122] and Iberia [123], which matches with some sulfur isotope values obtained here. On the other hand, the high diversity of mtDNA lineages of the Iberian Bronze Age, combined with the presence of almost only a single Y chromosome lineage, is certainly consistent with a patrilocality system of marriage in Iberia through the arrival of Steppe genetic ancestry [124].

There are also fewer references to a possible circulation of raw materials to make tools. Concerning lithic industries, a stemmed and winged lithic point from Los Avellanos I is made of tabular flint, a raw material expected in northern Burgos [125]. In contrast, the “Mucientes flint” obtained in the Valladolid province reached the southern foothills of the Cantabrian Mountains [126]. A promising research line is the characterisation of the copper minerals employed in manufacturing tools using Pb isotope analysis. In this sense, two of the coppers from El Casetón de la Era village (Valladolid) could have come from the prehistoric mines of La Profunda, El Aramo or El Milagro, located on both sides of the Cantabrian Cordillera [127]. The magnitude of these mines suggests a level of prehistoric exploitation that exceeded a pattern of local consumption or trade, suggesting a more extensive export system to other Iberian or Atlantic areas [128]. Particularly significant are the findings of three copper Palmella points, traditionally considered part of the suite of Bell-Beaker grave goods, in the Picos de Europa mountains [129], the highest range of the Cantabrian Mountain chain. This fact points to the use of these inhospitable mountain areas for hunting, grazing or transit of people between the Cantabrian coastal zone and the inland regions of the Northern Plateau. Finally, new bioarchaeological studies also support people’s movement during Late Prehistory, and thus, our results. Recent ^87^Sr/^86^Sr stable isotopes conducted in human individuals from Pico Ramos and Santimamiñe in Vizcaya showe evidence of mobility between the Pyrenees and the Cantabrian coast [84]. On the other hand, human genetic studies also indicate a great-scale migration of people in the Chalcolithic/Early Bronze Age transition, which introduced the genetic Steppe Ancestry into Iberia from Central Europe [124,130].

In summary, this body of evidence suggests a supraregional network of material exchanges, ideas circulation led by people movement among the farming populations of the Cantabrian Region, northern Iberian Plateau, and the Upper Ebro Valley, which is now been corroborated with sulfur isotopic data. However, this network would have a very limited scope and would be restricted to family relationships.

## 6. Conclusions

This research has generated a substantial sulfur isotope dataset of 142 specimens from 17 funerary sites of northern Spain, representing the first sulfur isotope approach during the Holocene in Iberia. These new results add to the pre-existing sulfur isotope results of 24 Iberian locations, allowing the creation of an isoscape model of Iberia using quantitative probabilistic approaches such as multivariate machine-learning regression and a random forest model. Our model identifies elevation, Bouguer anomalies, distance to the coast and strontium isotope values as primary drivers of sulfur isotope variation, confirming previous sulfur research. Today, the model constitutes an appropriate and credible tool for distinguishing human/animal mobility between coastal locations and inland territories of northern Iberia during Late Prehistory. The new screened dataset is conclusive for northern Iberia. However, due to the lack of sulfur isotope analysis, some geographical regions of Iberia, such as the Southern Plateau, the Ebro Valley, the Pyrenees, and the Northern Castilian Plateau, are still underrepresented. This data limitation among regions still provokes significant differences in the spatial distribution of prediction uncertainty, but confirms that the model predictions are consistent for the Atlantic coastal areas, moderately reliable for the Mediterranean ones and show uncertainty for some inland regions. Thus, this isoscape establishes an important milestone, but future sulfur isotope studies in animal and human bone collagen from late prehistoric contexts in those regions with limited data will provide a wider picture of the Holocene sulfur isoscape for the whole of Iberia. Although new analyses in other Iberian regions are needed, our study has proven to be a good tool for detecting movements of people between coastal and inland areas, laying the framework for using sulfur isotope systems as a tracer of human and animal mobility during Late Prehistory. We highlight sporadic migrations between the Cantabrian Region and the high Ebro valley and North Castilian Plateau, which is in line with the limited archaeological evidence documented so far.

## Supporting information

S1 Text Supplementary information. (DOCX)

S1-7 Tables S1 Table. Dataset with carbon, nitrogen and sulfur isotopic data of the archaeological sites included in this study. S2 Table. Descriptive statistics of stable isotope values of humans and animals by region. S3 Table. Descriptive statistics of stable isotope values of humans by period. S4 Table. Descriptive statistics of stable isotope values of animals by period. S5 Table. Statistical correlations between stable isotope values by period. S6 Table. Statistical comparisons between coastal/inland groups by stable isotopes and period. S7 Table. Regression matrix with site data of each variable used for sulfur isoscaping. (XLSX)
