# Supplementary material for: Sulfur as a proxy for identifying coast-inland human mobility in Northern Iberia during Late Prehistory
Source: PLoS One. 2025 Aug 28;20(8):e0330249. doi: 10.1371/journal.pone.0330249 (PMC12393764; doi:10.1371/journal.pone.0330249)
Supplement: S1 Text — (DOCX) [file pone.0330249.s001.docx]

**SUPPLEMENTARY INFORMATION**

# ****1. The sites****

## Coastal sites

### 1.1.1. El Espinoso (Ribadedeva, Asturias)

El Espinoso is a burial cave located in the village of La Franca (Ribadedeva), in the easternmost sector of Asturias province. The cave opens out from a 20-m-high limestone cliff, which dominates a closed valley, at only 200 m from the present Cantabrian shoreline. The cave entrance is oriented towards SW, and to access it is necessary to climb a four-metre-high part of the cliff. In a ~40m^2^ chamber at the end of the cavity, a collective burial site was discovered, where the human bones were scattered around the cave floor, highly fragmented and with neither anatomical connections nor associated grave goods [1]. Investigations at El Espinoso started in the early 1980s when an anthropic Upper Palaeolithic deposit was discovered in the cave vestibule [2]. Radiocarbon dating and the lithic industry recovered in a reduced test pit placed this occupation in the Archaic Magdalenian period [3]. The excavation of the funerary deposit was carried out in two different phases. In 1993, most of the human remains were recovered. In 2019, a second archaeological intervention was conducted to recover the last archaeological materials that remained at the site.

The anthropological study of more than 1,500 human remains has demonstrated that the cave was used as a collective burial place to deposit at least 25 individuals of different ages and sexes [4,5]. Taphonomic analysis of the assemblage showed poor anatomical representation in the collection and its high degree of fragmentation. The skeletal profile representation suggests, in addition to a great process of attrition, an anthropic origin for the differential preservation within the deposit related to the extraction of the most diagnostic bones from the cave once the bodies were decomposed to carry out a secondary deposition somewhere else, leaving in the cave many of the smaller bones. Taphonomic analysis also showed significant post-depositional diagenetic activity related to water circulation and the humidity typical of surface deposits in karstic environments [6]. Fifteen individuals from this funerary deposit were dated to the second half of the second millennium cal. BC (1235-1099 cal. BC) during a practically synchronic event corresponding to the Late Bronze Age [7]. The high δ^13^C results and the identification of polyhedral starch grains within their dental plaque underline the relevance of C_4_ plants in the diet of this human group, complemented by meat, dairy products, and other C_3_ cereals [7]. Provisional genetic results from El Espinoso’s individuals revealed a Steppe ancestry of almost 30%, constituting one of the Bronze Age sites with the most significant proportion of this ancestry in Iberia. 11 of 12 individuals share the same mitochondrial genome (H1j10). At the same time, the El Espinoso males belonged to two different Y-chromosome lineages (six males belong to R1b-M269, and two males belonged to I2-M223-PF692) [8].

### 1.1.2. El Hondón (Llanes, Asturias)

El Hondón cave is in San Roque de Acebal (Llanes), less than 3 km from the current coastline. The access to the cave is a small hole that opens into a limestone cliff from which a narrow and inclined duct of 4 meters long, followed by a 3-meter-deep vertical chasm, connects with the cave. The cavity consists of a large main room extending along several galleries and shorter blind passages. The cave was explored in 1988 by three young villagers who discovered human skeletal remains of five individuals (including several skulls) grouped in the central room of the cavity and an adjacent passage. The discoverers reported no other archaeological materials or grave goods. Even though they were handed over to the competent authorities, these remains are missing [5].

In 2019, under the direction of B. González-Rabanal and M. R. González Morales, a survey was carried out to confirm the existence or absence of human remains. The excavation revealed the presence of faunal bones and 50 human skeletal remains, including fragments of skulls that belonged to three individuals. Most of the bones were found in the main chamber on the surface and buried under a slight layer of stones. Another small set of remains was identified at the adjacent passage, corroborating the observations made by the initial discoverers. In addition, it was possible to appreciate the existence of a pile of stones in front of the excavation area, indicating that it was a burial and not a simple deposition of the bodies on the cave's surface. Three radiocarbon dates from the identified individuals indicate the use of the cave for funerary activities during the Late Neolithic (3346-2904 cal. BC). The diet of these individuals was consistent with a C_3_ terrestrial diet [5].

### 1.1.3. El Mirón (Ramales de la Victoria, Cantabria)

El Mirón cave is located in the eastern sector of the Cantabria province, in the municipality of Ramales de la Victoria. The site is located in the upper Asón Valley and c. 20 km from the present shoreline of the Bay of Biscay [9]. Since 1996, an international team led by L. G. Straus and M. R. González Morales has excavated the deposits of this cave. The site was divided into three excavation areas carried out in different sectors of the large vestibule of the cave and called the Cabin (near the mouth of the cave), the Mid-Vestibule Trench and the Corral (at the rear of the vestibule) [10]. Archaeological excavations in El Mirón Cave have revealed many cultural layers ranging from the late Middle Palaeolithic to the Bronze Age [11]. The Late Prehistory occupations (along with those of the Magdalenian) are particularly rich, and provided one of the most complete Holocene stratigraphical sequences of the Cantabrian Region [12].

Several human remains from two individuals (an adult and a child) were found at the back of the vestibule (Corral area) in 2013. The bones were located in different disturbed, superficial levels (Surface, 500, 501 and 502) without anatomical connection and located between the cave wall and the large, engraved block located in the Corral area. The revision of materials from the 1996 and 1997 archaeological campaigns also provided more human remains that joined the bones excavated in 2013. In the Corral, there were no intact levels of Late Prehistory as there are in the Cabin, possibly because this area underwent levelling of the sedimentary fill for use as a livestock pen during historical periods, which led to the removal of materials from different periods and presumably the modification of the original position of the human remains [12]. The individuals have been dated to the Chalcolithic (2459-2207 cal. BC) and the Early Bronze Age (1883-1548 cal. BC) [13], respectively, while the stable isotope analysis suggests a C_3_ terrestrial diet [5].

### 1.1.4. El Abrigo de la Castañera (Villaescusa, Cantabria)

El Abrigo de La Castañera is south of Santander Bay, in the town of Obregón, municipality of Villaescusa. The site is an 85-m^2^ deep rockshelter, part of a seven-cave karst complex, oriented towards the NE. The site was surveyed in the late 1960s [14] and partially excavated during the 1970s [15]. Later, it was subsequently studied by local archaeologists [16]. Since 2011, a multidisciplinary research team led by C. Vega-Maeso has been excavating the site, providing 63 stratigraphic units that have allowed the discovery of a stratigraphical sequence of more than 4 m deep that covers all the Late Prehistory [17].

The rockshelter has evidence of domestic use during the Neolithic, including living areas [18], and a possible cattle stable during the Chalcolithic, due to several fumière-type deposits rich in charcoals and organic matter identified during the excavation [19]. Besides, a well-preserved Bronze Age stratigraphy linked to funerary events with 379 human remains and almost 2000 sherds. Pottery showed a decoration pattern based on small incisions or impressions combined with incised lines, typical of this time and region [17]. Human remains from at least six individuals (three adults and three infants) were recovered during the older and recent excavations associated with faunal remains and the pottery mentioned above [5,20]. Three individuals were dated in the Early Bronze Age during the transition between the 3rd and 2nd millennium cal. BC (2287-1766 cal. BC) [17]. The δ^13^C and δ^15^N values of these humans analysed were consistent with individuals consuming a diet dominated by terrestrial C_3_ resources and animal protein [21].

### 1.1.5. La Fragua (Santoña, Cantabria)

La Fragua site is a relatively small cave located 125 meters above sea level on the south-eastern slope of Mount Buciero, on the shore in Santoña (Cantabria). Excavations from 1991 to 1996 provided a well-defined stratigraphic sequence. Archaeological levels of the site consist of the following: Level 4 (Magdalenian), Level 3 (Azilian), Level 2 (sterile archaeologically) and Level 1 (Mesolithic) [22]. During the excavations, a deposit of mixed fill, limestone blocks, pottery, and animal and human bones was identified inside a trench that had cut entirely through the stratigraphic sequence of the cave and that was filled in part with material from Level 1. The trench was sealed by hearths made by shepherds who used the cave in the last century. The trench was dug subsequently to the deposition of Level 1, but its absolute chronology is unknown, although it is likely that it was made at a time in Late Prehistory [23]. A monk seal bone recovered inside the trench was dated to the Neolithic [24].

Seven human remains were identified in the fill of the trench: a fragment of the right humerus, a left coxal bone, three hand phalanges, a right third cuneiform and a decidual tooth. They belong to an MNI of two (one adult and one infant). However, a second individual with only a decidual tooth is unverifiable. No diagnostic bones like cranium, mandible or long bones have been documented. Therefore, the human record is composed of small and low-density remains, including bones that are generally not preserved in secondary burials but are often found in primary burials [5]. One hundred forty sherds of vessels and 517 domesticated and wild faunal bone remains were identified inside the trench [25]. At the bottom of the trench appeared a slab within a small ditch. This slab could have constituted a marker for the burial. This set of evidence could indicate an opening of the trench to remove the prominent human bones of the skeleton for secondary burial elsewhere. A radiocarbon date from a human humerus provided a chronology referring to the Early Bronze Age (2199-1978 cal. BC) [23]. This individual reported δ^13^C and δ^15^N values typical of a predominantly terrestrial diet based on C_3_ plants and animal protein [7]. Genetic analysis of this individual revealed that he is a male with 25% Steppe ancestry [8].

### 1.1.6. La Llana (Llanes, Asturias)

Another human individual studied here came from La Llana cave, located in the coastal village of Andrín (Llanes). The archaeological site was discovered in 1981 during an archaeological survey [26] and was later excavated by M. R. González Morales between 1982 and 1985. The cave has two entrances: a giant mouth in a deep doline at its northern end and a small rockshelter to the south (now almost fully obstructed by anthropic sediments). The archaeological excavation inside the cave was divided into two different areas: Sector A, where a human burial was found, and Sector B, where a Mesolithic shell midden was identified [2]. During the excavation, on the surface of the Mesolithic shell midden, objects identified as a waste of different kinds of technological processes (potsherds, a small metal piece, a polished wild boar tooth and three bevel‐cut bones) were found [17]. The pottery constituted a homogeneous assemblage of 199 fragments: 188 sherds and 11 rims. A minimum of four vessels was identified. Two (vessels 1 and 2) presented profuse incised decoration and could be classified within the “Trespando” pottery group. In addition, there were two more vessels: one small, non‐decorated and almost complete container (vessel 3) and another fragmented (vessel 4) that was not typologically identified [27].

The skeleton belonged to an adult male individual lying on the surface, with some bones covered by a stalagmitic flowstone firmly cementing them to the cave floor. This individual presented a perimortem otitis media, which was probably the cause of death [28]. A taphonomic study of the human remains showed that some bones were still articulated. In contrast, other bones had been displaced during the decomposition stage, suggesting that the body was moved from its primary position (probably in Sector B) due to an anthropic decision or by natural agents [5]. Radiocarbon dates were obtained from a coxal fragment of the human skeleton and a fragment of bevel‐cut bone. Both remains were dated to the first half of the second millennium cal. BC, corresponding to the Middle Bronze Age (1618-1510 cal. BC) [17]. The stable isotope analysis of their bone collagen provided δ^13^C and δ^15^N values of a diet influenced by C_3_ plants and animal protein [7].

### 1.1.7. Los Avellanos I and II (Alfoz de Lloredo, Cantabria)

Los Avellanos I and II sites are located in the central-western part of Cantabria province (northern Iberia) in the town of La Busta, municipality of Alfoz de Lloredo. Both caves are situated on a small calcareous hill, six kilometres from the present shoreline, which dominates a plain delimited to the north and west by Monte Barbecha and the south and east by the Saja River. The entrance of Los Avellanos I is oriented towards SE, which is four meters wide and two meters high, leading to a vestibule where the archaeological assemblage was encountered at surface level [29]. The site was surveyed in the 1960s by the Sanz de Sautuola Archaeological Group, which undertook exploratory excavations, although the results were not published in detail [30]. During those works, the excavators documented the presence of human remains, lithic industry (highlighting a stemmed and winged lithic point) and a pottery collection consisting of 156 fragments. Of these, 14 are wheel-thrown ceramics and indicate the use of the cave in historical times, possibly the medieval period. The rest of the pottery collection, attributed to the Chalcolithic and Bronze Age, comprises handmade vessels, which have been placed into 20 groups according to their typological, technological and decorative characteristics. Due to its surface deposition in a karstic context, this collection had a high fragmentation level, and >20% of the potsherds were covered with calcareous concretions. However, the shape of the three vessels could be determined. Vessel 1 comprises 31 fragments, part of an ovoid pot decorated using incised lines and small impressions. A single, greyish, polished fragment enabled the reconstruction of part of Vessel 2, a vessel with a closed profile and a high-carinated shoulder. Finally, 27 pieces comprise the upper profile of a big storage pot, Vessel 3, with a convex moulding below the rim [17]. The human remains were found disarticulated and scattered across the cave floor, partially buried, and mixed with other archaeological materials [30]. Los Avellanos II is tunnel-shaped, with two narrow mouths oriented to the south and east. The archaeological remains were found in a small room east of the entrance (three meters wide and two meters high). The site was discovered and excavated during the 1970s by local amateurs without stratigraphic precision, and the cave was subsequently used as a cattle stable in modern times. Among the materials included in the Regional Archaeological Inventory of Cantabria were numerous fragments of human remains and a polished axe that are missing from the archive today. No data about the disposition of these human bones were documented during these amateur excavations [31], and no subsequent archaeological campaigns have taken place in the cave.

A bioarchaeological study was conducted alongside radiocarbon dating and stable isotope analyses (δ^13^C, δ^15^N, δ^34^S) of human (*n=* 7) and animal (*n=* 3) bone collagen [5]. Both caves were used as burial locations between the 4th and 3rd millennia cal. BC (3518-2575 cal. BC). Taphonomic analysis of the human remains showed post-depositional activity within both sites after the burial. The δ^13^C and δ^15^N results showed that the prehistoric individuals ate a predominantly terrestrial diet, with animal protein from meat and likely dairy products as vital resources. The δ^34^S results showed heterogeneity among the people buried in both caves. Most individuals had higher δ^34^S values typical of living in coastal areas. However, three individuals had lower δ^34^S values, indicative of living further inland for at least some of their lives, suggesting human mobility between inland and coastal areas [32].

## 1.2. Inland sites

### 1.2.1. El Agua (Santiurde de Reinosa, Cantabria)

El Agua cave is located in the village of Lantueno (Santiurde de Reinosa). A cavity in the middle of the mountain acts as a fossil upwelling. The cave collected water from the municipality due to the active circulation outside waterfalls in modern times. Today, the entrance to the cave is constantly flooded. In 1982, inhabitants discovered several human remains on the Surface, including a complete mandible and tibia, which were deposited in the Department of Prehistory of the University of Cantabria. Recently, the cave was explored in 2015 by the Cantabrian Speleo Club, which located new human remains that most likely belong to the same context as those discovered in 1982. The human remains that stay on the site were located at the base of a large room that is accessed after 35 meters of rectilinear and partially flooded main gallery. The bones rest on the cave's surface, and their extremities are partially in anatomical connection, while the rest of the skeleton is removed and eroded very close to the first. All the human remains belong to an adult male individual, dated in the Middle Bronze Age (1731-1505 cal. BC). Their carbon and nitrogen stable isotopes showed a C_3_ terrestrial diet [5].

### 1.2.2. Arroyal I (Alfoz de Quintadueñas, Burgos)

Arroyal I is in the San Antón paramo, within the Alfoz de Quintanaduenas, Burgos. It is oriented to the East, over the valley of the Ubierna river. The site was excavated between 2011 and 2012 by a research team from the University of Burgos [33]. The site is a megalithic tomb comprising a rectangular chamber (3 × 3.5 m), a long corridor (6 m) and a stone mound. The tomb was used as a collective burial location for over 400 years during the Late Neolithic (3325-2907 cal. BC) before it was abandoned [34]. During the Chalcolithic, the tomb came back into use when it was extensively remodelled. At that time, Neolithic layers were almost entirely removed; the corridor was filled with rocks and sediment (as a closure event), and a stone wall and a floor of limestone blocks were built inside the chamber. Following this, several consecutive and isolated burials were interred. One contained a young individual, dated to 2465–2211 cal. BC was buried with four vessels (two bell beaker bowls and two carinated ones), surrounded by long human bones and skulls from previous contemporaneous burials. In the next phase of activity, the dolmen was closed, re-using materials from the site in a secondary position (2461-2206 cal. BC) and the external mound height was increased. Finally, an isolated pit grave containing a female human body was created inside the mound, which dates to 2348–2200 cal. BC [34].

At least nine individuals have been identified in the different burial uses of the tomb. The human stable isotope values from Arroyal I demonstrated a main cluster that was eating a C_3_ diet that included animal protein (meat and potentially milk), except one individual, who, based on its sulfur isotopes, derives from another area such as the Cantabrian coast [21]. Faunal remains showed the great importance of cattle, followed by ovicaprines and pigs. The age profiles suggested the use of meat and dairy products in the first two species and meat exploitation in the latter [35]. The genetic analysis of most Arroyal I individuals showed that most Beaker-complex-associated individuals lacked steppe affinities and were genetically most similar to preceding Iberian populations. However, steppe-related ancestry was already present in two individuals, demonstrating that gene flow into Iberia was not uncommon and arrived on the peninsula associated with this archaeological culture [36].

### 1.2.3. El Hornazo (Villímar, Burgos)

El Hornazo is a pits field located near the Villimar neighbourhood (Burgos), discovered during the archaeological monitoring of the works of the Burgos railway. It is situated in a flat space in the contact zone between the Vena River terraces and the paramo's slopes. In 2004, a research team excavated an area of almost 1 ha where 179 different structures were discovered, including pits, ditch pits, pit graves and post holes [33]. This site represents a Chalcolithic settlement divided into three activity areas: habitation, storage and resource processing. In the storage area, two pit graves with associated individuals were documented. AMS dates of the human remains belong to the Pre-Beaker Chalcolithic (2860-2473 cal. BC) [37]. The first grave (Pit 103) included a human subadult buried with a bowl infilled with sediment and few domestic remains. In the second pit (Pit 140), another subadult was found buried crouched. Deposits within Pit 140 contained domestic remains, including pottery and animal bones. A single biconical bead was registered as a grave-good inside Pit 140, in conjunction with the radiocarbon dates, the stylistic and typological attributes of archaeological remains determined that the entire aggregate of negative structures was formed during a single phase of occupation [33]. Cattle dominated the faunal assemblages at El Hornazo, followed by ovicaprids and the presence of dogs and rabbits. Some immature cattle were also identified. The Chalcolithic humans from El Hornazo had δ^13^C and δ^15^N values, indicative of a terrestrial diet of animal protein and plant products such as wheat species [21].

### 1.2.4. Fuente Celada (Alfoz de Quintadueñas, Burgos)

Fuente Celada is also located in Quintanadueñas. It was excavated in 2008 by the same research team that excavated El Hornazo. It is a settlement with many rock-cut pits (‘silos’) that was occupied initially during the Neolithic, containing a burial that was dated at that time. The main focus of occupation occurred in the Early Chalcolithic [33]. In addition to a domestic area (containing ‘silos’, post holes, mud fragments used in the huts, stone mortars and other domestic remains), human remains were discovered inside several pits, dating to the Chalcolithic (2860-2474 cal. BC). Pit 19 contained a young woman buried in an unusual inverted position (almost vertically placed, with the head at the bottom and the feet at the top). This pit also contained the most considerable accumulation of potsherds found at the site (1018 fragments, 19.5% of the total). In Pit 5, the disarticulated human remains of three individuals (a subadult and two male adults), also belonging to the Chalcolithic, were recorded [38]. Cattle dominated the faunal assemblages at El Hornazo, followed by ovicaprines and dogs, and carpological data have reported the presence of some wheat microremains [33]. The Chalcolithic humans from Fuente Celada had δ^13^C and δ^15^N values typical of a terrestrial diet of animal and plant resources [21]. The Neolithic individual was genetically analysed, and it showed a higher European First Farmers ancestry coming from Anatolia and scarcely mixed with the Western Hunter-Gatherer ancestry [39].

### 1.2.5. Kaite (Merindad de Sotoscueva, Burgos)

Kaite cave is a subhorizontal and cortical passage that constitutes the uppermost and isolated level of the Ojo Guareña karst system. It is located at 840 m a.s.l. at the NE end of the blind valley of San Bernabé, situated at the bottom of the Sotoscueva Valley. Its main mouth opens 140 meters above the town of Cueva de Sotoscueva. It is one of the highest a paleosinkhole of the Guareña River, from which a broad panoramic view of the valley is commanded [40]. Neolithic and Bronze Age habitation areas are preserved in its two entrances: 1) The Portalón, a valley facing entrance demarcated in prehistoric times by a wall; and 2) the current access (placed at El Portillo cave entrance), which is the result of a ceiling collapse. The original entry to this sector provided a debris cone which infilled the access to the deepest zone, its symbolic sector, with a first funerary space, followed by a large room with semi-naturalistic rock art [41,42].

The Edelweiss Speleological Group (GEE) discovered the Kaite cave in 1958. In 1969, some children from the village of Quisicedo unblocked access to the symbolic area, discovering several human remains on the surface, carrying skulls to the village, where the priest buried them in the cemetery [42]. Between late 2017-2018, a team led by A. I. Ortega surveyed the site, where a 1970s tourism project had dug a trench 60 cm deep. The study showed the presence of human remains out of context, without anatomical connection, and located on both margins of the trench, displaced at the time of their discovery. Radiocarbon dating of the four individuals identified placed the use of the cavity during the Late Neolithic/Chalcolithic (3091-2295 cal. BC). Additionally, the analysis of their bone collagen provided δ^13^C and δ^15^N values typical of a C_3_ terrestrial diet [5].

### 1.2.6. Los Cinchos (Quirós, Asturias)

The human skeleton from Los Cinchos cave was discovered in a cavity at an altitude of 1870 meters above sea level in the Natural Park of Las Ubiñas (Asturias). The remains were found in 2010 at the bottom of a complex karstic system composed of four chasms [43]. The skeleton was located in a narrow crack in the bedrock and deposited on an irregular platform of debris clogging the crevice. It had been placed in a sitting position; however, it did not maintain its anatomical disposition due to post-depositional movements caused by the decomposition of the corpse and postmortem manipulations of the bones. No structures, ornamental elements, or grave goods were documented. The anthropological study determined that it was an 18-19-year-old male juvenile with a height of 166 ± 6.9 cm. Even though dental eruption had ended (all four third molars had erupted), the individual exhibited a considerable delay in epiphyseal fusion, with some epiphyses fused or in the process of fusion while others remained completely unfused, which has been linked to an undetermined congenital disease. On the other hand, the paleopathological study ruled out any perimortem injury resulting from an accident [44]. A plausible interpretation of the find suggested that the individual descended into the pit voluntarily or was forced down with the assistance of others, ultimately becoming trapped with no means of escape. A bone sample from this individual was radiocarbon dated to 2014-1771 cal. BC and then attributed to the regional Early Bronze Age [43].

### 1.2.7. Palomera (Merindad de Sotoscueva, Burgos)

Cueva Palomera represents the most important cavity of the Ojo Guareña karstic system. This system, placed on the southern slope of the Cantabrian Mountain range and the upper Ebro basin [42], consists of 14 connected caves and a network of galleries that reach a total length of 110 km, constituting one of the most extended underground systems in Europe [45]. Ojo Guareña also preserves an unusual wealth of cave fauna (54 species) (46), and an exceptional record of human occupation and frequency in the past (with more than 80 archaeological sites) (42,47-53), which led him to declare Cultural and Natural Heritage of Spain. The entrance to Cueva Palomera is located at the bottom of an old sinkhole of the Villamartín stream [47], providing access via the slope of *Rampa de Palomera* to the cave's fourth level of karst and its main gallery, during almost one kilometre, where most of the archaeological record is concentrated, with around 40 evidences of different activities (40,42). Regarding the habitat area, in 1972, M. Soledad Corchón carried out a test pit in the vestibule, documenting a stratigraphic sequence of about 5m deep with prehistoric ceramics. The results of this excavation were not published, and the materials are stored at the Archaeological Museum of Burgos [40]. Besides, in *Rampa de Palomera,* there is a natural ditch of runoff erosion channel that shows a powerful cultural sequence with several fumière-type stratigraphic units dated from the Neolithic to the Bronze Age. These findings suggested an important habitation area from Late Prehistory in the cave entrance [47]. Further inside, there are seven areas with rock art created by hunter-gatherers and farming communities from the Azilian to the Bronze Age [50-53]. Also noteworthy are the exceptional human traces of the *Galería de las Huellas* [49], and an Individual of the Iron Age which was lost in a labyrinthine area of the *Vía Seca* (40,42,46-53). In this research, we include two single funerary sites discovered in singular and difficult-to-access spaces around the main gallery in 1981 and 2002 and recovered by A. I. Ortega in the surveys of the Inventory of Archaeological Cavities in Burgos (2016-2019) [48].

The first burial place is *Terraza del Enterramiento de la Galería Principal,* located 500 meters from Palomera entrance on a ledge seven meters high and one meter wide above the *Galería Principal*. The human skeleton was deposited over the floor surface of the ledge, which was expressly prepared for its deposition in partial anatomical connection and without any grave goods. During the postmortem interval, the place was disturbed because of faunal activity, which modified the primary position of the burial. The second funerary site is *Galería Sepulcral de la Sima Dolencias,* located on the upper level of *Sima Dolencias,* a stringing abyss through which the Villamartín stream waters rushes after saving a drop of 54 meters high in thaw seasons and torrential rains. The individual was on the floor surface, partially covered by a very thin layer of silts, in the lateral corridor of a small northern chasm. This little passage is 30 meters above the *Galería Principal* and 1000 meters from the Palomera entrance. The skeleton appeared in anatomical connection and was flexed on his right side, although he did not have grave goods either. At a later time, this deposit was affected by digging a trench, resulting in the loss of the skull and other long bones. Both individuals were anthropologically identified as adult males, and they were radiocarbon dated in the Early Bronze Age (2014-1751 cal. BC) [48]. Additionally, the δ^13^C and δ^15^N values of these humans analysed were consistent with individuals consuming a diet dominated by terrestrial C_3_ resources and animal protein [5].

### 1.2.8. La Quebrantada (Montorio, Burgos)

La Quebrantada cave is located SE of Montorio village, about 28 km north of Burgos on the southern edge of the Cantabrian Mountains, on the cliffs above the Saint Cecilia stream. It consists of a small cavity, barely 6 m long, with a maximum width of 4 m, almost infilled with sediments. In 2016, furtive activities caused damage to an important Late Prehistory funerary site. During this digging, more than a meter of sediments, with abundant human remains, some faunal bones, pottery and two polished axes, were extracted from the site [54]. A. I. Ortega carried out a rescue intervention through two excavation campaigns (2018 and 2019), whose record provided a stratigraphic sequence of 1.5 m thick, an ensamble of material culture elements typical of Late Prehistory, and an important set of human bones with more than 1,100 human remains belonging to an MNI of 19 individuals of all sexes and ages. The radiocarbon dataset revealed the diachronic funerary use of this small cave during all of the Late Prehistory [55]. Four individuals were dated to the Late Neolithic (3760-2930 cal. BC), one individual was dated to the Chalcolithic (2480-2200 cal. BC), and 14 individuals were dated to three different periods of the Bronze Age, spanning from almost a millennium (2020-1010 cal. BC).

### 1.2.9. Trulla (Montorio, Burgos)

Trulla cave is also located in Montorio village, close to La Quebrantada cave. During the archaeological intervention in La Quebrantada, neighbours noticed the presence of human remains and sherds in this cavity. Trulla is a rockshelter with a narrow lateral crevice infilled with sediments. This deposit was partially emptied in modern times, resulting in the discovery of human and animal bones and two pottery vessels. A rescue intervention was carried out by A. I. Ortega in 2021 and 2022 to recognise the potential archaeological site and recover the archaeo-anthropological remains from this burial site, some grouped at the cave entrance. Two human individuals have been identified, and they were radiocarbon dated to the Chalcolithic (2930-2700 cal. BC) and the Late Bronze Age (990-820 cal. BC) [55].

# 2. Stable isotope results

## 2.1. Cantabrian sites

### 2.1.1. El Espinoso

From El Espinoso cave, 16 specimens dated in the Late Bronze Age were isotopically analysed. The humans (*n*= 14) had δ^13^C values ranging between ‑17.8 and ‑14‰ (*X̅=* ‑15.6‰) and δ^15^N values between 9.1 and 10.4‰ (*X̅=* 9.5‰). Two faunal specimens were analysed as a local baseline: a cow specimen had a δ^13^C value of ‑22.1‰ and a δ^15^N value of 4.1‰, and a sheep had a δ^13^C value of ‑20.6‰ and a δ^15^N signature of 3.7‰. The animals had δ^13^C values typical for a terrestrial European C_3_ ecosystem. At the same time, the carbon isotope results from humans showed that they were significantly enriched in ^13^C, suggesting an intake of C_4_ plants or marine foods. Higher δ^15^N isotope values are seen in individuals that consumed marine foods, as the aquatic food chain is longer than the terrestrial one, and the lower δ^15^N isotope values observed at El Espinoso suggest that these individuals were not consuming marine foods. The El Espinoso animal samples had δ^15^N values 3–4‰ lower than the human ones, constituting the expected relationship between consumers and prey in a terrestrial ecosystem. Consequently, the most likely hypothesis to explain the higher δ^13^C in El Espinoso individuals could be a significant consumption of C_4_ plants, likely millets. Isotope measurements of modern millets have provided δ^13^C values from ‑10 to ‑12‰, and δ^15^N values from 3 to 4‰, and isotope measurements of Iron Age millets have reported δ^13^C and δ^15^N values around -10‰ and 4‰. Millet has a protein content of approximately 10%; thus, a significant intake of these crops is required to detect a substantial change in collagen isotope ratios [7].


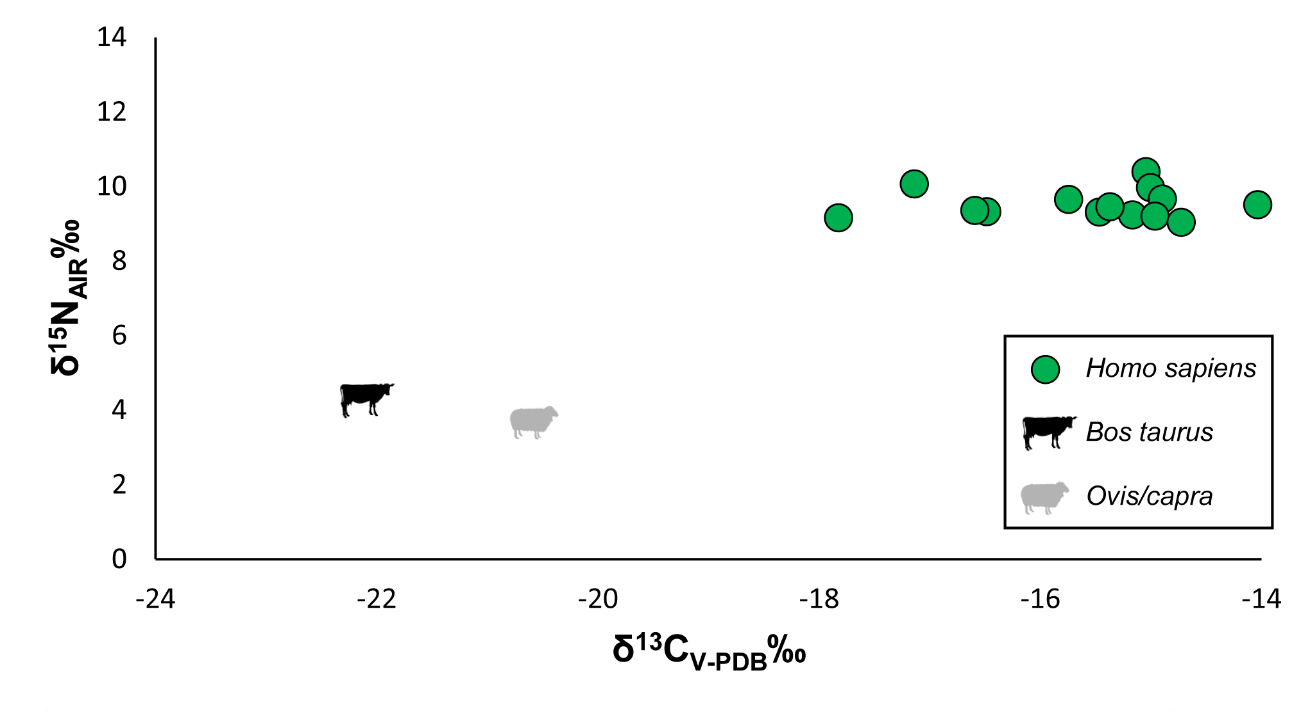


Fig S1. Scatterplot of δ^13^C and δ^15^N values on human and animal bone collagen analysed at El Espinoso.

Regarding the sulfur isotope values, all the specimens reported very homogenous results. The humans yielded δ^34^S values between 13.5 and 16.9‰ (*X̅=* 14.9‰), while the cow had a δ^34^S signature of 16.2‰, and the sheep a δ^34^S value of 13.5‰. Although the consumption of marine or freshwater resources can produce elevated δ^34^S signals, the fact that both humans and herbivorous showed a similar range of δ^34^S values, between 13.5‰ and 16.9‰, rules out the consumption of aquatic resources as the cause of these high sulfur isotope values. These results may be more likely associated with the isozone where they were living than with the type of diet. The high δ^34^S isotope values of humans and animals from El Espinoso would reflect the effect of marine spray, which can reach up to 30km inland, affecting sulfur isotope signals. This evidence suggests that the individuals buried in the El Espinoso cave would be locals and have lived near the coast around the site.


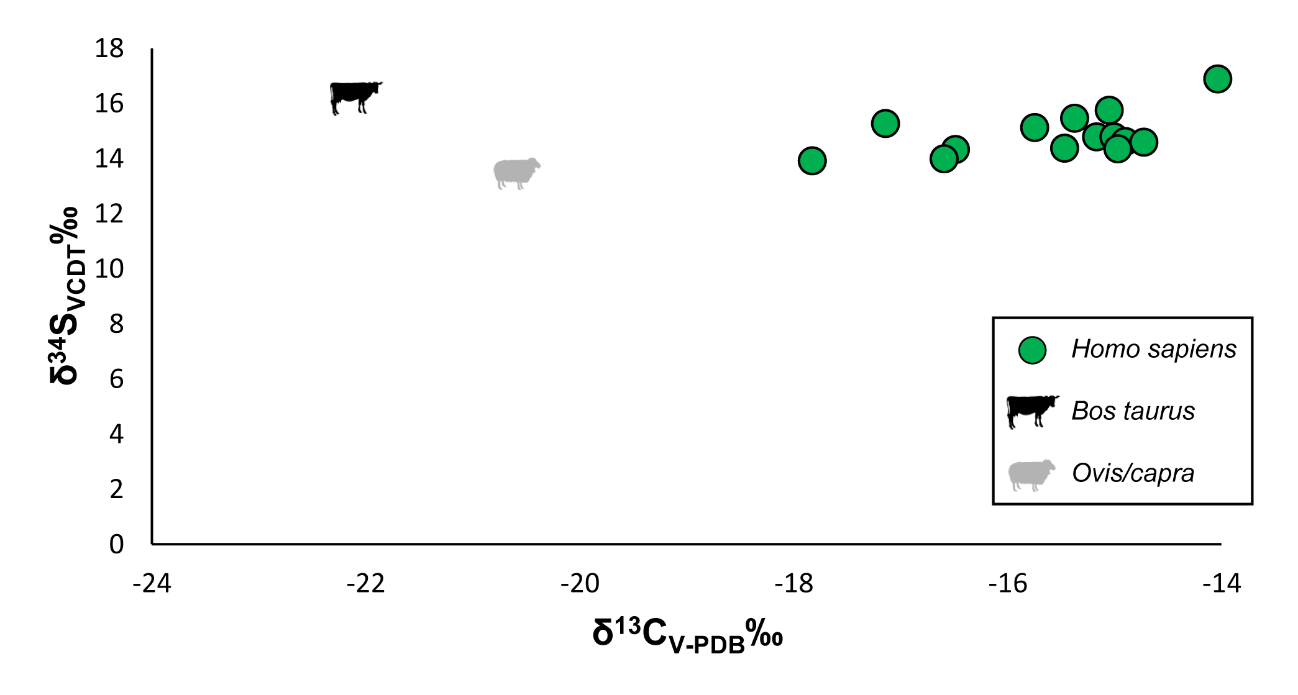


Fig S2. Scatterplot of δ^13^C and δ^34^S values on human and animal bone collagen analysed at El Espinoso.

### 2.1.2. El Hondón

The humans analysed from El Hondón (*n=* 3), dated to the Late Neolithic, showed δ^13^C values ranging between ‑21.1‰ and ‑20.4‰, and δ^15^N values between 9.4‰ and 9.8‰. Regarding the fauna samples, the dog provided a δ^13^C value of ‑18.6‰ and a δ^15^N value of 9.4‰; the cow had a δ^13^C of -22.5‰ and a δ^15^N of 5.5‰; the ovicaprine a δ^13^C value of ‑21.3‰ and a δ^15^N of 5.3‰; and finally, the pig had a δ^13^C value of ‑17.4‰ and a δ^15^N value of 6.2‰. The isotope results of the humans stand out for their homogeneity. The scarcity of faunal remains recovered at the site has limited further inferences at the local level. However, the δ^13^C values of humans and animals are consistent with those of typical terrestrial C_3_ plant ecosystems. The only exception is a subadult pig (SUC717), which presents a higher δ^13^C value typical of the consumption of marine resources or C_4_ plants, indicating that this specimen may be influenced by its age, the location where it was hunted, or it could constitute a modern intrusion. On the other hand, the δ^15^N values of humans are 3-4‰ higher than the herbivores analysed, constituting the usual relationship established between carnivores and herbivores in a terrestrial ecosystem. The dog, like humans, had δ^13^C and δ^15^N isotope values consistent with a carnivorous diet, like other canids from the Late Prehistory of Northern Iberia, suggesting a diet derived from human food waste. The δ^13^C and δ^15^N isotope values of humans from El Hondón reflect a terrestrial diet based on the intake of C_3_ resources and animal protein.


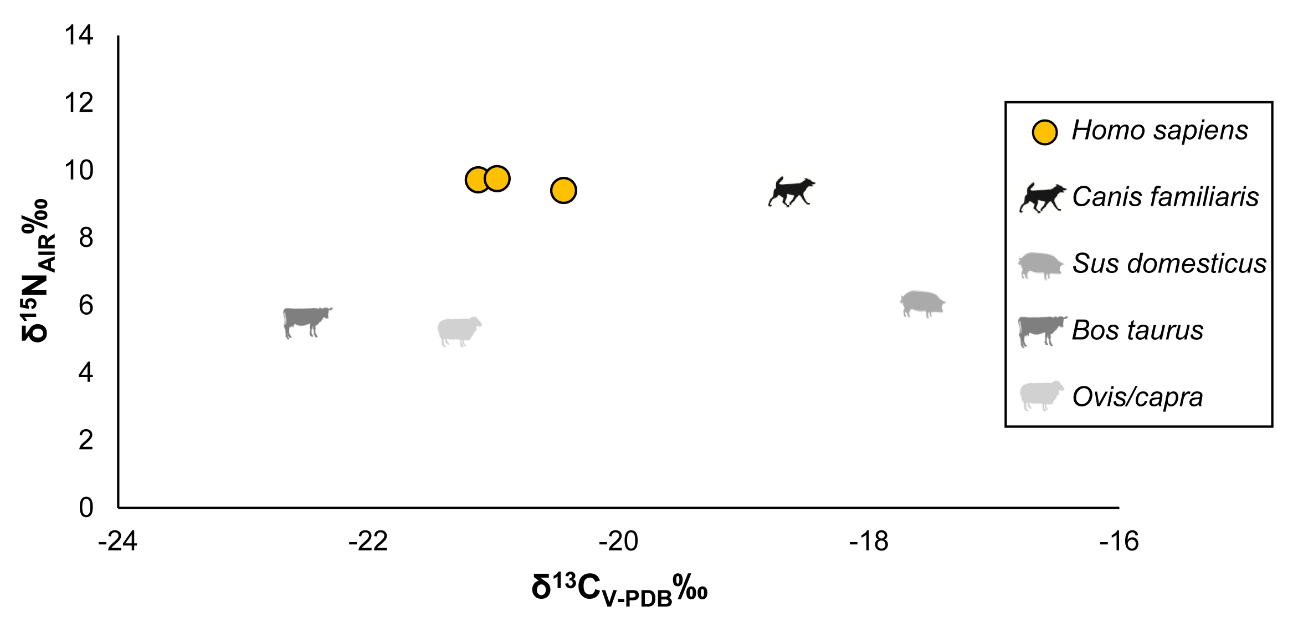


Fig. S3. Scatterplot of δ^13^C and δ^15^N values on human and animal bone collagen analysed at El Hondón.

The sulfur isotope values obtained in these humans were more diverse. Humans showed a δ^34^S values of 7.4‰, 12.6‰ and 14.1‰, indicating greater heterogeneity than that observed with carbon and nitrogen isotopes. In contrast, the animals analysed provided more similar values. The dog had a δ^34^S value of 15.9‰, the cow 17.1‰, the ovicaprine 18.1‰ and the pig 17.6‰. These results indicate that the animals lived in a terrestrial ecosystem rich in sulfur, while humans exhibited lower isotope signals, particularly in one individual (SUC713). These results reflect three trends with different sulfur isotopic compositions, one with higher δ^34^S values ranging between 15.9‰ and 18.1‰, represented by the faunal specimens; a second group represented by two humans, with δ^34^S values slightly lower than animals that fluctuate between 12.6‰ and 14.1‰; and another human with an even lower δ^34^S isotope value of 7.4‰. These contrasts can be ruled out as a consequence of the diet practised by humans and animals since humans have revealed a homogeneous diet based on their δ^13^C and δ^15^N values, and the animals have a heterogeneity in these isotopes that is not observed with sulfur. Therefore, the δ^34^S values suggest different places of origin. The animals' δ^34^S values indicate their occupation of coastal areas influenced by marine aerosols, which aligns with the site's location. Two humans present slightly different values to the animals. Still, they can be associated with an isozone not far from the coastal platform. At the same time, the isotope signal of the last human suggests that it is a non-local individual and comes from another place with a much lower δ^34^S baseline, characteristic of a more arid and inland environment.


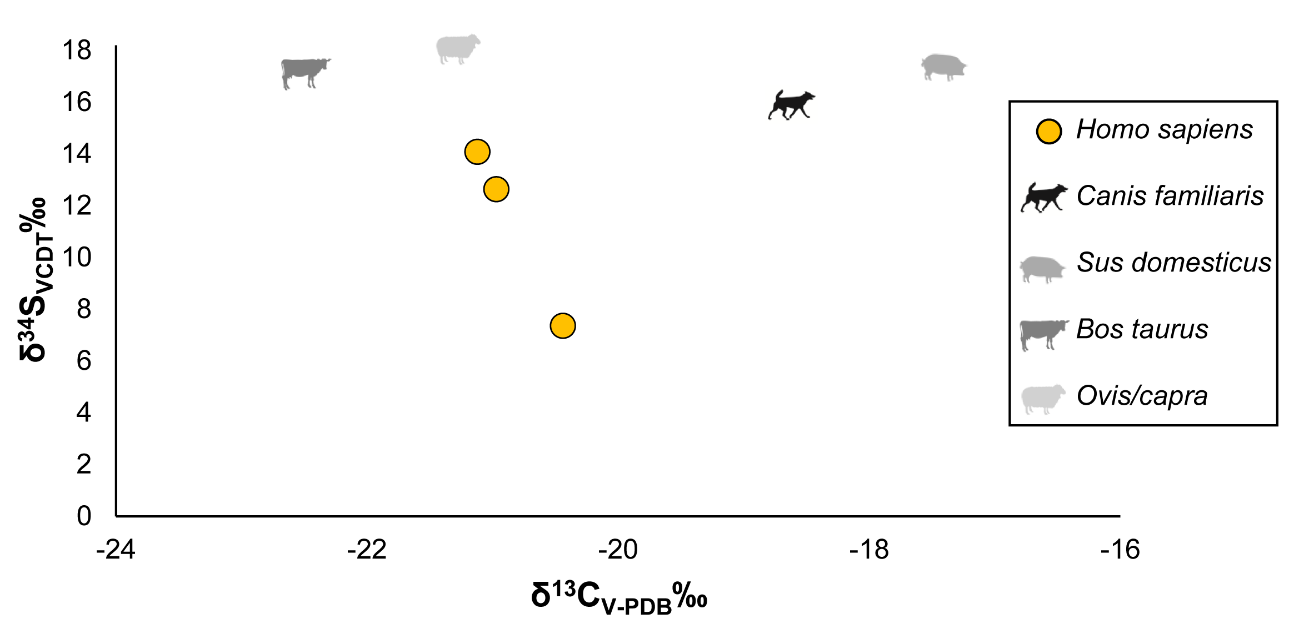


Fig. S4. Scatterplot of δ^13^C and δ^34^S values on human and animal bone collagen analysed at El Hondón.

### 2.1.3. El Mirón

The humans analysed provided δ^13^C values of ‑21.5‰ and ‑21.3‰, and δ^15^N values of 9.1‰ and 9.4‰. The red deer specimens showed δ^13^C values ranging between -22‰ and -20.9‰ (*X̅=* -21.6‰), while the δ^15^N values ranging between 3‰ and 4.3‰
(*X̅=* 3.6‰). The human δ^13^C values are characteristic of a terrestrial C_3_ plant ecosystem. In contrast, the δ^15^N values are typical for consumers within the terrestrial food chain compared to the red deer individuals sampled from the same site and periods. These results suggest a mixed terrestrial diet based mainly on C_3_ plants and meat intake. These results rule out the consumption of marine resources or C_4_ plants, at least from an isotope perspective. This pattern has been observed in all Bronze Age Cantabrian sites. The fact that the infant individual presents the same dispersion of values as the adult indicates that it had already been weaned and incorporated into the group's diet.


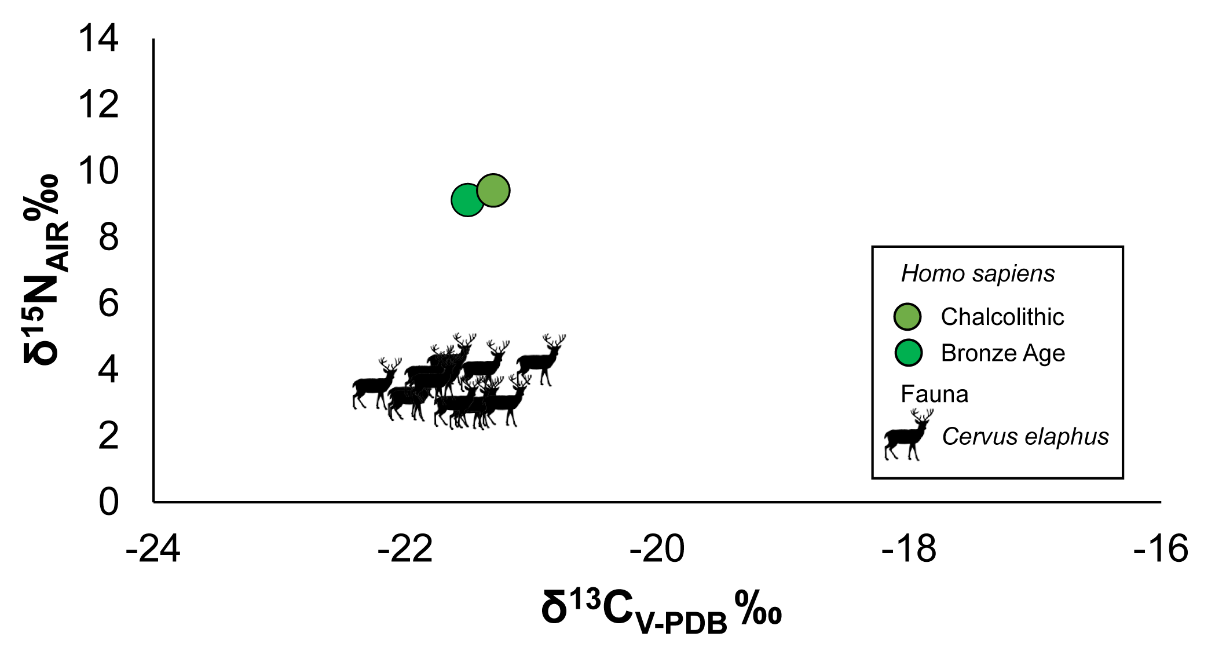


Fig. S5. Scatterplot of δ^13^C and δ^15^N values on human and animal bone collagen analysed at El Mirón.

The humans had δ^34^S values of 15.1‰ and 15.6%. The red deer showed δ^34^S values ranging between 9.6‰ and 16.7‰ (*X̅=* 13.4‰). Their δ^34^S values are very high, suggesting that these individuals likely spent the last years of their lives in an isozone with a high δ^34^S baseline. As their carbon and nitrogen values do not support the hypothesis that a dietary factor causes the observed δ^34^S, it seems plausible that these individuals came from a coastal territory affected by marine aerosols or they would have lived in an area with evaporitic rocks, which can yield high levels of sulfur formed by marine sulfates millions of years ago, similar to those found in deposits affected by marine spray. The red deer from the Chalcolithic and Bronze Age periods have similar sulfur isotope values to the humans, supporting the idea that humans were local inhabitants of this sector of Asón valley. However, the Neolithic red deer showed slightly lower sulfur values, indicating that they were hunted in an area with a lower sulfur δ^34^S baseline, possibly further inland.


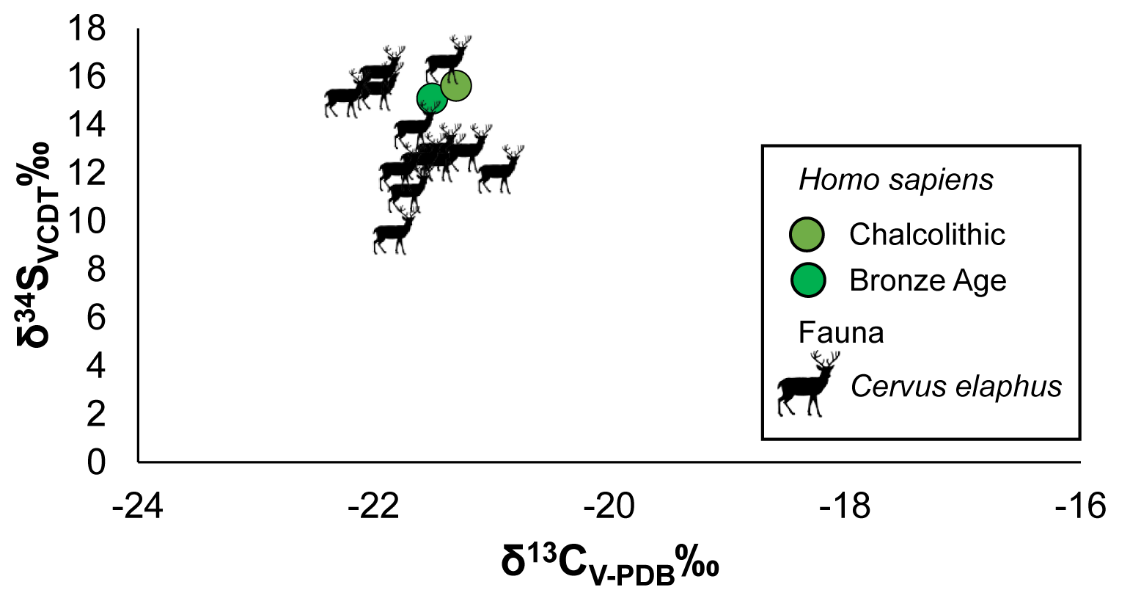


Fig. S6. Scatterplot of δ^13^C and δ^34^S values on human and animal bone collagen analysed at El Mirón.

### 2.1.4. El Abrigo de la Castañera

The humans analysed from El Abrigo de la Castañera (*n=* 3) belong to the Early Bronze Age and have δ^13^C values ranging between ‑21.3‰ and ‑20.5‰ and δ^15^N values ranging between 9.3 and 10‰. Of the faunal remains analysed, two of the three pigs have stable isotope values typical of consuming in an open landscape with δ^13^C values of ‑22.0‰ and ‑21.2‰. The other pig specimen (SUC08) has a much lower δ^13^C value, typical of an animal feeding in a woodland environment under the influence of the canopy effect. The δ^15^N values also reflect slight differences in the trophic level of the three pigs. The first two have higher δ^15^N values, indicating a possibly more omnivorous diet, while the third pig has lower δ^15^N, indicating a more herbivorous diet. These contrasts between suids suggest different animal management practices, suggesting the stabling of some pigs in woodland environments to avoid crop damage, or one of them could potentially be a wild boar. Finally, an ovicaprine specimen had δ^13^C and δ^15^N values of ‑21.6‰ and 3.7‰, respectively. They indicate a terrestrial ecosystem dominated by C_3_ plants and the typical trophic step between consumers and their prey. The human δ^13^C and δ^15^N isotope values at El Abrigo de la Castañera reflect a homogeneous diet based on consuming C_3_ terrestrial plants and animal protein, probably meat and dairy products [21].


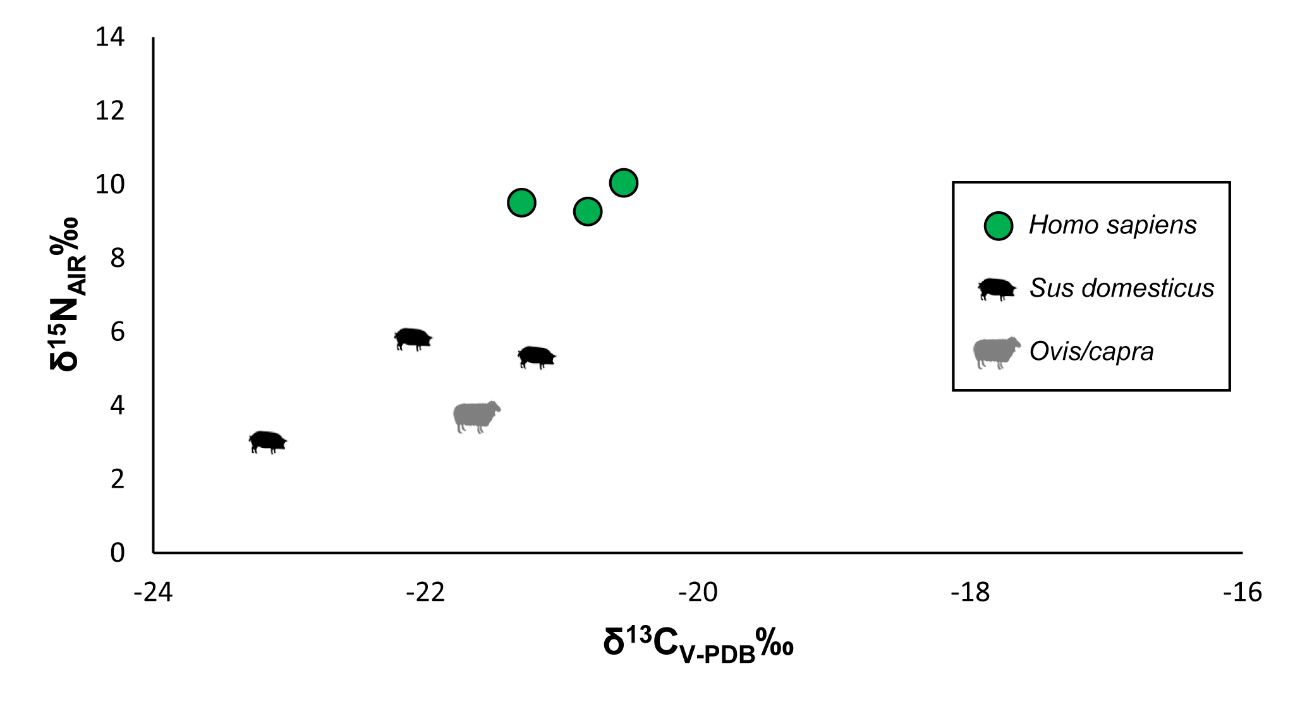


Fig. S7. Scatterplot of δ^13^C and δ^15^N values on human and animal bone collagen analysed at El Abrigo de la Castañera.

Regarding sulfur, the results obtained for δ^34^S values indicate greater heterogeneity in human and faunal samples. Humans had sulfur isotope values of 2.9‰, 15.3‰ and 2.1‰, while pigs had isotope signals of 12.3‰, 14.1‰ and 14.6‰ and the ovicaprine a δ^34^S value of ‑3.7‰. These results reflect two groups with different sulfur isotope compositions: one with higher δ^34^S values ranging between 12‰ and 15‰, represented by a human (SUC10) and three pigs, and another group with lower δ^34^S values close to 0, represented by two humans (SUC09 and SUC11) and the ovicaprine, which showed an even lower and negative sulfur isotope signal. This heterogeneity, both in humans and animals, can be ruled out as a consequence of their diet since humans have revealed a homogeneous diet based on the δ^13^C and δ^15^N values and pigs show heterogeneity in these isotopes that is not observed with sulfur. Therefore, the δ^34^S values suggest that both groups would have been in locations with different δ^34^S baselines in the last years of their lives. The first group would reflect the typical δ^34^S values of a coastal region influenced by the sea spray effect, which fits with the site's location. By contrast, the lower δ^34^S values of the second group can be associated with an inland location with a continental climate. The lower δ^34^S signals obtained in these two humans and the ovicaprine suggest that these individuals are non-locals from another geographical area.


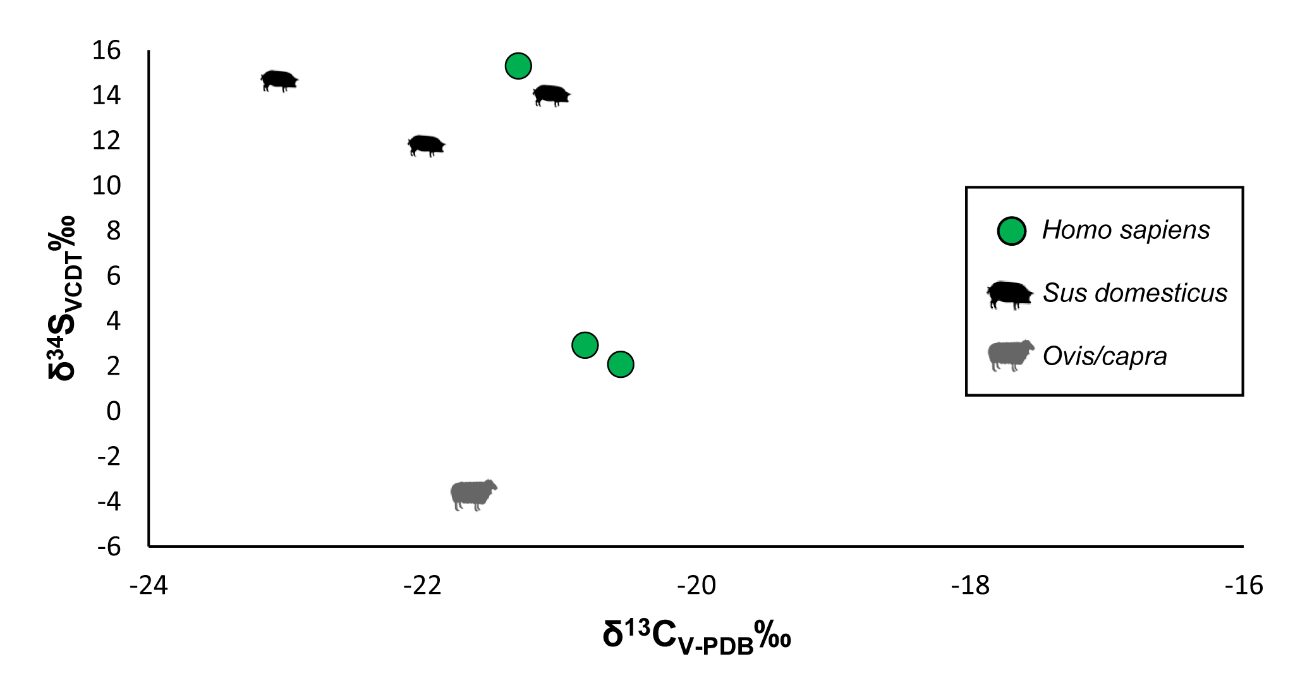


Fig. S8. Scatterplot of δ^13^C and δ^34^S values on human and animal bone collagen analysed at El Abrigo de la Castañera.

### 2.1.5. La Fragua

The only human (SUC443) studied from this site showed a δ^13^C value of ‑20.4‰, a δ^15^N value of 9.2‰ and a δ^34^S of 14.8‰. The δ^13^C value of this individual is consistent with the typical values of a terrestrial ecosystem of C_3_ plants. At the same time, the δ^15^N value indicates the expected relationship between the trophic level of carnivores and the terrestrial food web. These results suggest a mixed terrestrial diet based mainly on C_3_ plants and meat protein, without isotope evidence of the consumption of marine resources or C_4_ plants, which is in line with the rest of the Bronze Age individuals analysed so far. The δ^34^S value reveals that this individual would have lived the last years of his life in an isozone with high δ^34^S baseline values, probably on the coastal platform where the cave is located.


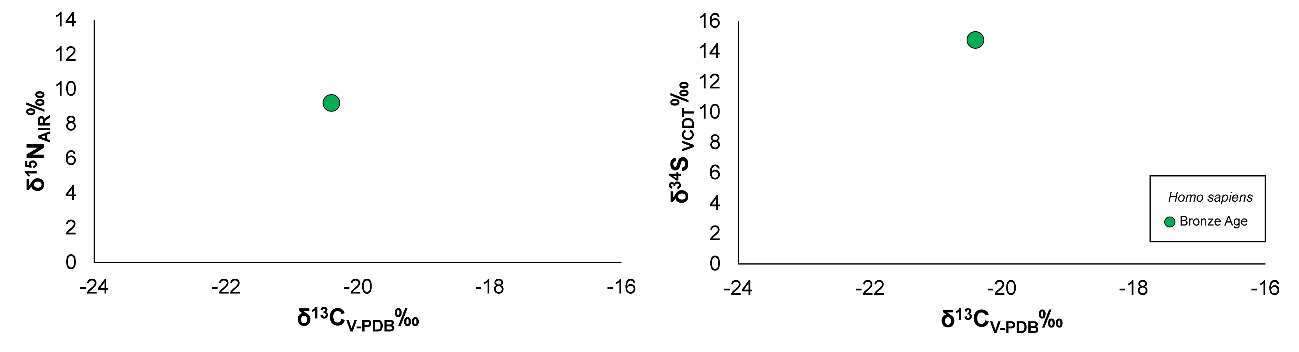


Fig. S9. Scatterplot of δ^13^C and δ^15^N, and δ^13^C and δ^34^S values on human bone collagen analysed at La Fragua.

### 2.1.6. La Llana

The human (SUC357) showed a δ^13^C value of ‑21.1‰, a δ^15^N value of 9.2‰ and a δ^34^S value of 14.5‰. The δ^13^C value of this individual is consistent with the typical values of a terrestrial ecosystem of C_3_ plants. In contrast, the δ^15^N value reflects the higher trophic level of this individual compared to the herbivores. These results suggest a mixed terrestrial diet based mainly on C_3_ plants and meat protein, with no isotope evidence of consumption of marine resources or C_4_ plants. The δ^34^S value indicates that this individual is local and was living in an isozone with a high δ^34^S baseline value, probably on the coastal platform where the site is located.


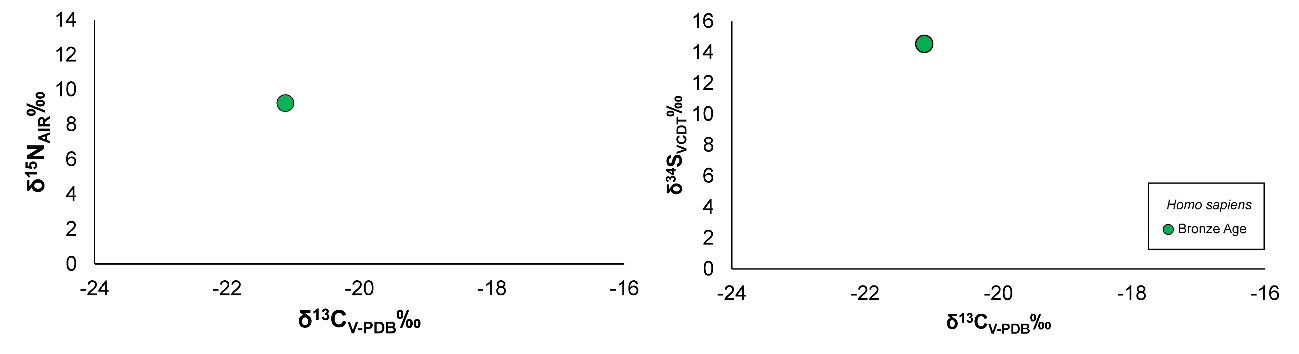


Fig. S10. Scatterplot of δ^13^C and δ^15^N, and δ^13^C and δ^34^S values on human bone collagen analysed at La Llana.

### 2.1.7. Los Avellanos I and II

The humans analysed from Los Avellanos I (*n=* 3) belong to the Late Neolithic and Chalcolithic periods and had δ^13^C values ranging between ‑21.3‰ and ‑20.9‰ and δ^15^N values between 8.7‰ and 9.3‰. The individuals from Los Avellanos II (*n=* 4), dating to the Chalcolithic, had δ^13^C values ranging between ‑21‰ and ‑20.5‰ and δ^15^N values ranging between 8.8‰ and 9.3‰. Three faunal specimens were available to sample from these sites. The ovicaprid from Los Avellanos I had a δ^13^C value of ‑21.4‰ and a δ^15^N value of 4.7‰. The cow from Los Avellanos II had a δ^13^C value of ‑21.9‰ and a δ^15^N value of 4.4‰. The juvenile pig from Los Avellanos II had a δ^13^C value of ‑17.6‰ and a δ^15^N value of 7.1‰. This δ^13^C value can be associated with the consumption of C_4_ plants, such as millets, but could also be due to the consumption of marine foods or even could derive from a much later chronological period. The δ^13^C values of the humans buried at Los Avellanos I and II show homogeneity in their diet, consistent with typical values for a terrestrial C_3_ North Iberian ecosystem during the Holocene. These individuals have δ^15^N values 3–4‰ higher than the herbivores, especially regarding the cow and ovicaprine, the standard relationship between consumers and prey. Both suggest that the prehistoric humans from Los Avellanos I and II were eating a mixed C_3_ diet that included animal protein [32].


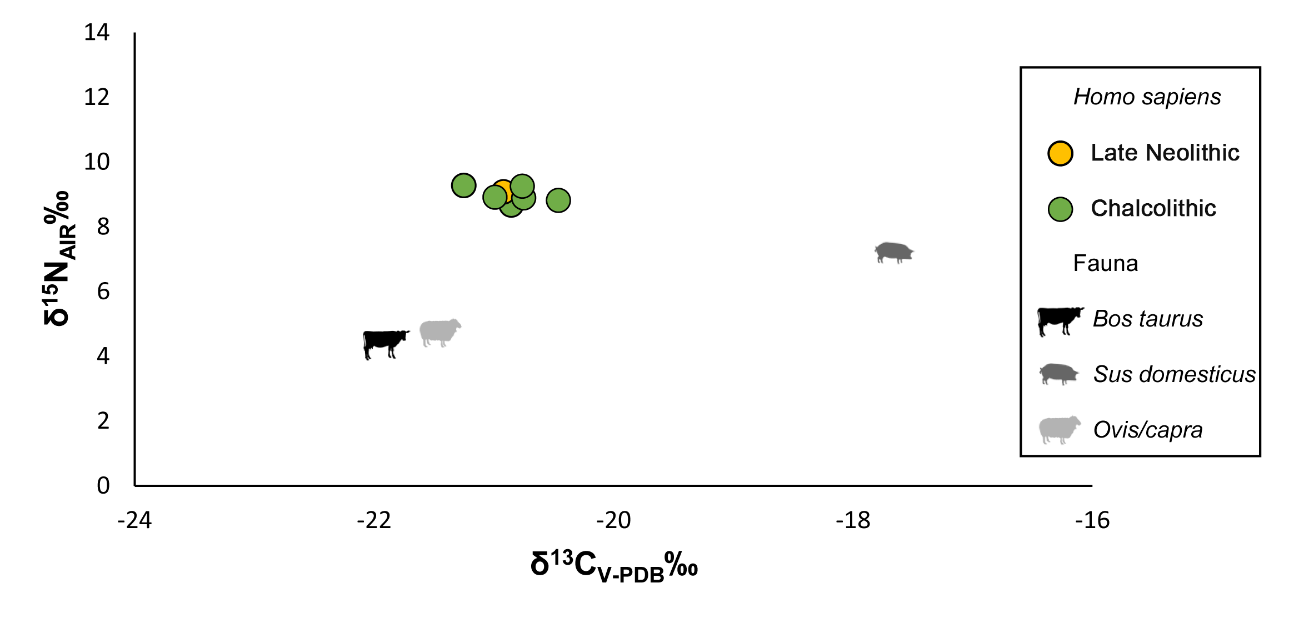


Fig. S11. Scatterplot of δ^13^C and δ^15^N values on human and animal bone collagen analysed at Los Avellanos I and II.

The human individuals from Los Avellanos I yielded δ^34^S values of 4.6‰, 9.6‰ and 13.5‰. These results reflect very diverse δ^34^S signatures within the burials. For Los Avellanos II, three humans had δ^34^S values ranging between 13.4‰ and 14.8‰, which are more homogeneous than at Los Avellanos I. A further Chalcolithic individual from Los Avellanos II (SUC365) had a lower δ^34^S value of 10.5‰. The three faunal samples also had δ^34^S values of 13.7‰ (ovicaprine), 14.8‰ (*Bos taurus*) and 14.6‰ (*Sus domesticus*). Therefore, four humans and three faunal specimens had values within the same range (13.4–14.8‰), indicative of all of them living in the same geographical location. However, three humans fell outside this range (SUC359, SUC360 and SUC365), suggesting that they lived in an area with a lower δ^34^S baseline, causing them to have lower δ^34^S values than the previous group. Likely, the main group of humans and animals with higher δ^34^S values predominantly lived near the coast, with sulfur signatures affected by the sea spray effect, typically producing elevated δ^34^S values. The three humans with lower δ^34^S signatures could reflect an isotope signal typical of inland territories, as δ^34^S values decrease with distance from the coast. A likely location of origin of these people could be further south, towards the high Ebro valley or the North Castillian Plateau, inland regions with a cool and dry continental climate [32].


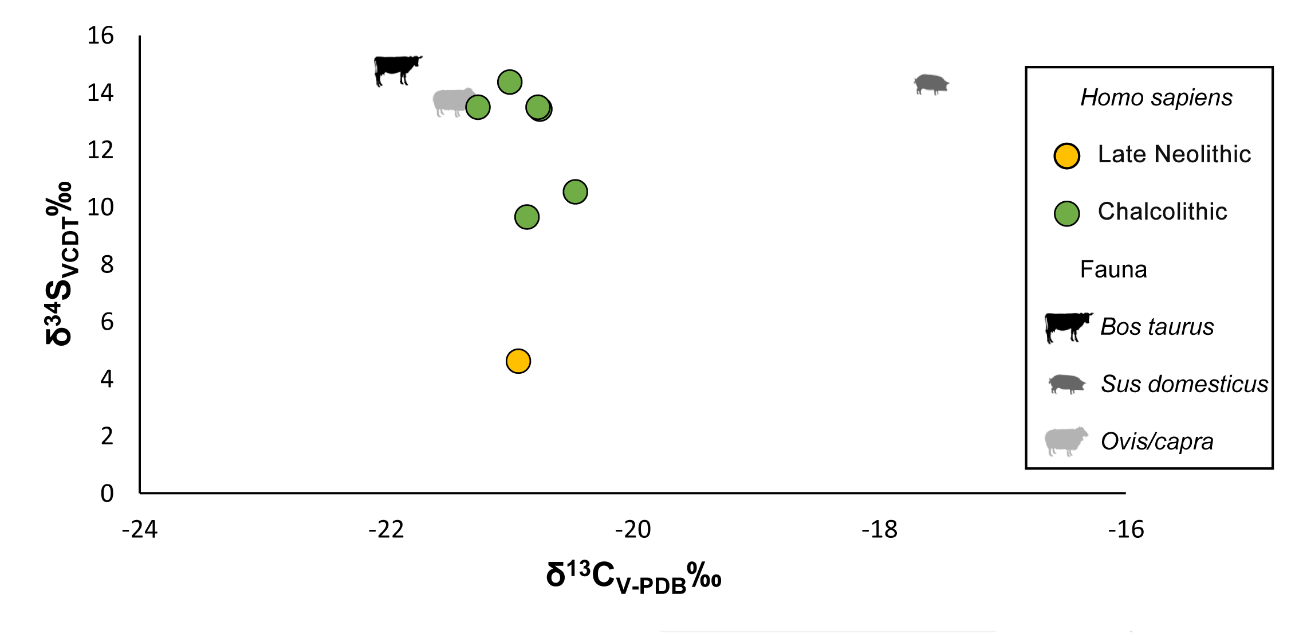


Fig. S12. Scatterplot of δ^13^C and δ^34^S values on human and animal bone collagen analysed at Los Avellanos I and II.

## 2.2. North Plateau sites

### 2.2.1. El Agua

Stable isotope analysis of this individual (SUC771) showed a δ^13^C value of ‑20.4‰, a δ^15^N value of 8.9‰ and a δ^34^S value of 6.2‰. The δ^13^C value of this individual is consistent with the values of a terrestrial ecosystem of C_3_ plants. Similarly, the δ^15^N value is typical for carnivores within the terrestrial food chain for northern Iberia. These results suggest a terrestrial mixed diet based mainly on C_3_ plants and meat protein, with no isotope evidence of consumption of marine resources or C_4_ plants. On the other hand, the δ^34^S value is low, typical of an isozone with a lower δ^34^S baseline, such as the south of the Cantabrian Region.


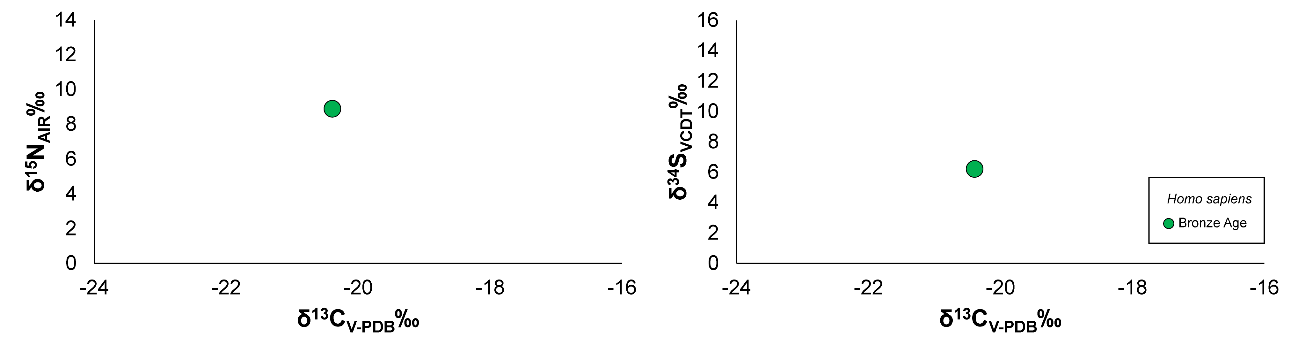


Fig. S13. Scatterplot of δ^13^C and δ^15^N, and δ^13^C and δ^34^S values on human bone collagen analysed at El Agua.

### 2.2.2. Arroyal I

The human stable isotope values from Arroyal I (*n=* 9) form a cluster of individuals dated to the Late Neolithic, Chalcolithic and Beaker cultures, with δ^13^C values ranging between ‑19.3‰ and ‑19.7‰, and δ^15^N values ranging between 9.2‰ and 10.9‰. One individual (SUC28) plotted away from this main cluster with a lower δ^13^C value of ‑20.8‰, while the δ^15^N value of 10.3‰ is similar to the δ^15^N values observed in the main cluster. The available faunal specimens studied from this site were three sheep, with δ^13^C values ranging between ‑21‰ and ‑20.3‰, and δ^15^N values ranging between 6.8‰ and 8.5‰; two cows, which reported δ^13^C values of ‑21.1‰ and ‑19.4‰ and δ^15^N values of 6.1‰ and 9.3‰; a horse with a δ^13^C signature of ‑21.1‰ and a δ^15^N signature of 9.4‰ and a dog with δ^13^C and δ^15^N values of ‑19.7‰ and 9.2‰. The humans have δ^15^N values 3–4‰ higher than the herbivores sampled from the site. This is a typical relationship between consumers and prey during the Holocene and suggests that the humans were eating a C_3_ diet that included animal protein (meat and milk) [21].


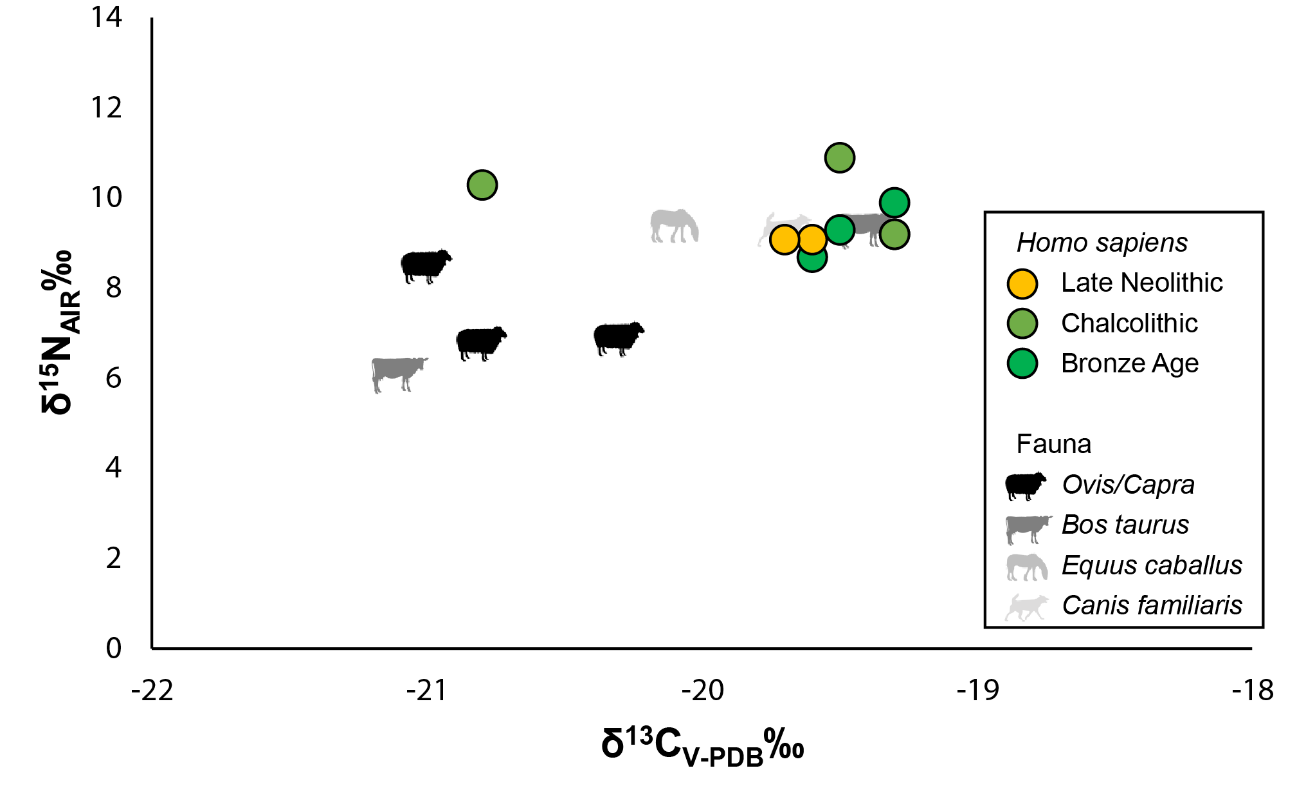


Fig. S14. Scatterplot of δ^13^C and δ^15^N values on human and animal bone collagen analysed at Arroyal I.

Regarding sulfur, the results obtained for δ^34^S values indicate greater homogeneity between human and faunal specimens. The δ^34^S values of humans ranged between 8.5‰ and 14.7‰. However, the main group reported sulfur isotope values between 8.5‰ and 11.7‰ and one individual (SUC 28) was completely separated from this cluster with a δ^34^S value of 14.7‰, as previously seen with the carbon isotope values. The three sheep had δ^34^S values of 11‰ and 12.4‰, while the two cows reported a sulfur value of 7.1‰ and 10‰. The horse had a δ^34^S value of 7.1‰ and the dog a δ^34^S value of 10.4‰. This homogeneity reflects that the δ^34^S values suggest that humans and animals would have been in locations with a lower bioavailable δ^34^S baseline in the last years of their lives. These values are consistent with the typical values of an inland area, which fits with the site's location. The lower sulfur isotope signals obtained for the main group of humans and animals suggest that these individuals are locals. By contrast, the higher sulfur isotope value of the individual mentioned above can be associated with a coastal location and an Atlantic climate, suggesting that this individual was a non-local.


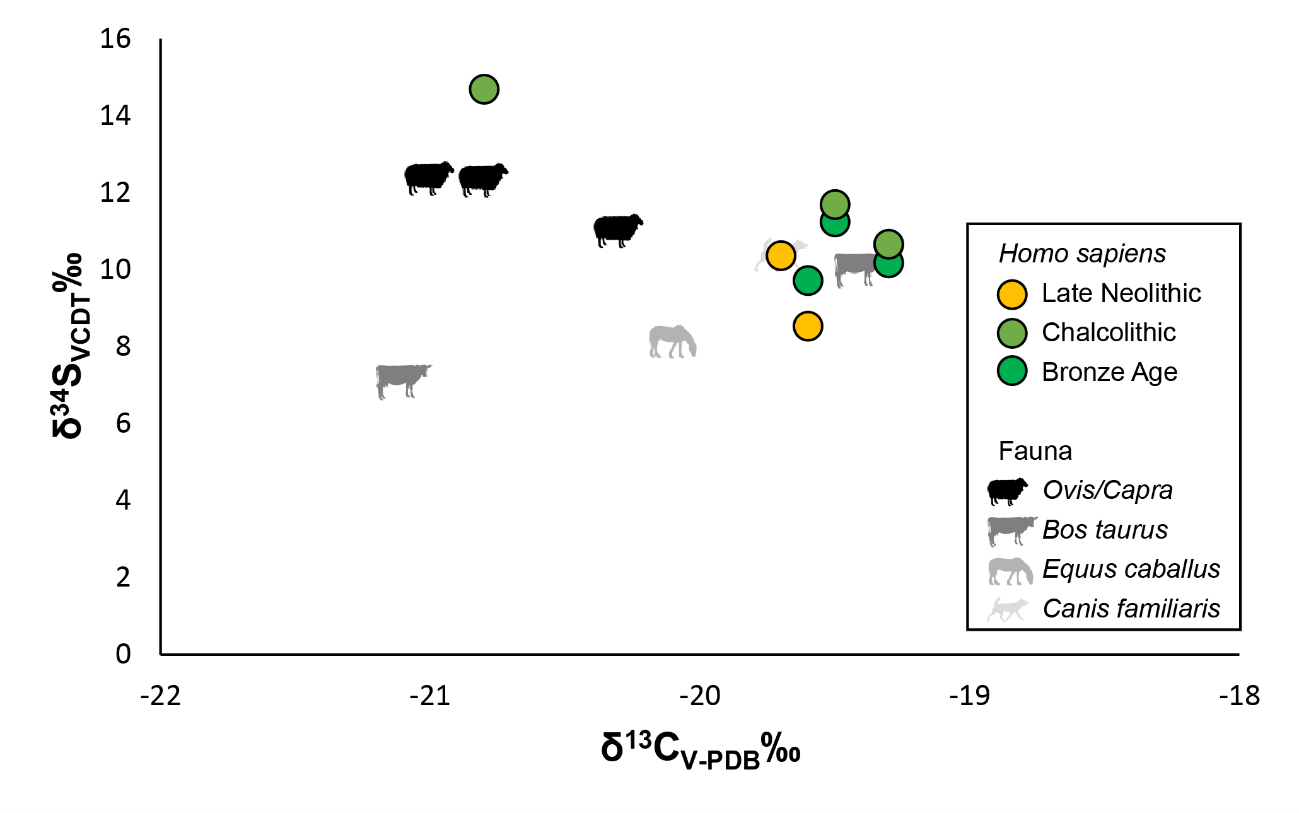


Fig. S15. Scatterplot of δ^13^C and δ^34^S values on human and animal bone collagen analysed at Arroyal I.

### 2.2.3. El Hornazo

The Chalcolithic humans from El Hornazo (*n=* 2) have δ^13^C values of ‑19.2‰ and ‑19.7‰ and δ^15^N values of 8.6‰ and 9.5‰, indicative of a terrestrial diet of animal protein in addition to plant products. Of the dog specimens sampled (*n=* 4), SUC265 has values indistinguishable from the humans analysed (δ^13^C ‑19.1‰, δ^15^N 8.2‰). The other dog specimens plotted have δ^13^C values between ‑20.2‰ and ‑20.7‰ and δ^15^N values ranging between 6.3‰ and 6.4‰ and demonstrating differing dietary behaviour of dogs at the site. The cattle (*n=* 6) have δ^13^C values lying between ‑19.3‰ and ‑20.8‰, with δ^15^N values ranging between 5.3‰ and 8.3‰, although SUC264 showed a lower δ^13^C value of ‑19.3‰. The ovicaprines had δ^13^C values ranging between ‑20.6‰ and ‑18.8‰ and δ^15^N values ranging between 6.2‰ and 9‰. Exceptionally, two ovicaprines and one cow are plotted with the human specimens exhibiting higher δ^13^C and δ^15^N values. Finally, a red deer specimen was analysed, indicating a δ^13^C value of ‑20‰ and δ^15^N value of 5.8‰ [21].


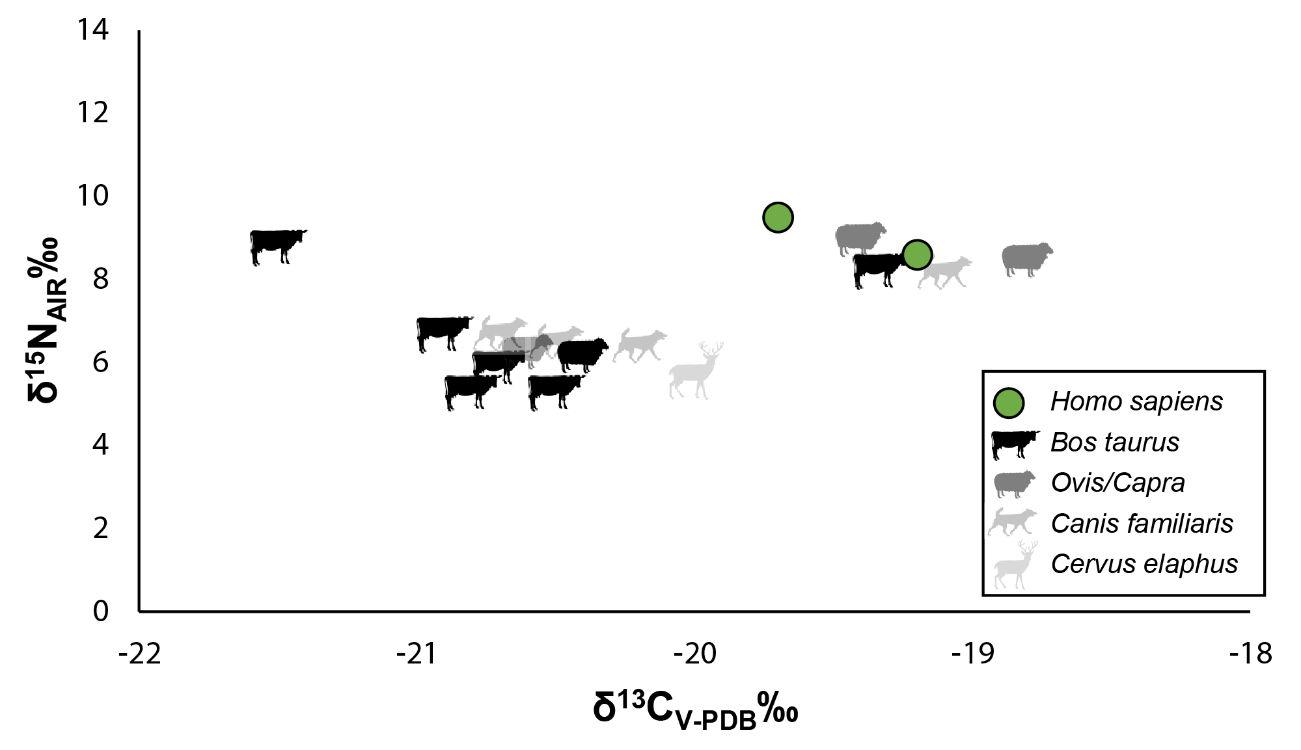


Fig. S16. Scatterplot of δ^13^C and δ^15^N values on human and animal bone collagen analysed at El Hornazo.

Concerning sulfur, the humans from El Hornazo reported a δ^34^S value of 8.2‰ and 10.2‰, constituting low sulfur isotope signatures. All the faunal species used as baseline provided a very coherent sulfur isotope pattern similar to humans. Cows had δ^34^S values ranging between 7.4‰ and 10‰, while sheep reported δ^34^S values ranging between 8.3‰ and 10.6‰. On the other hand, dogs had δ^34^S values ranging between 9.7‰ and 10.3‰. Finally, the only red deer individual had a δ^34^S signature of 8.1‰. Therefore, the sulfur isotopic compositions of humans and animals reflected a very homogeneous pattern, characterised by lower sulfur isotope values typical of inland areas and dry climates. This pattern suggests that the El Hornazo individuals are locals, living the last years of their lives in the surroundings of the place where they were buried.


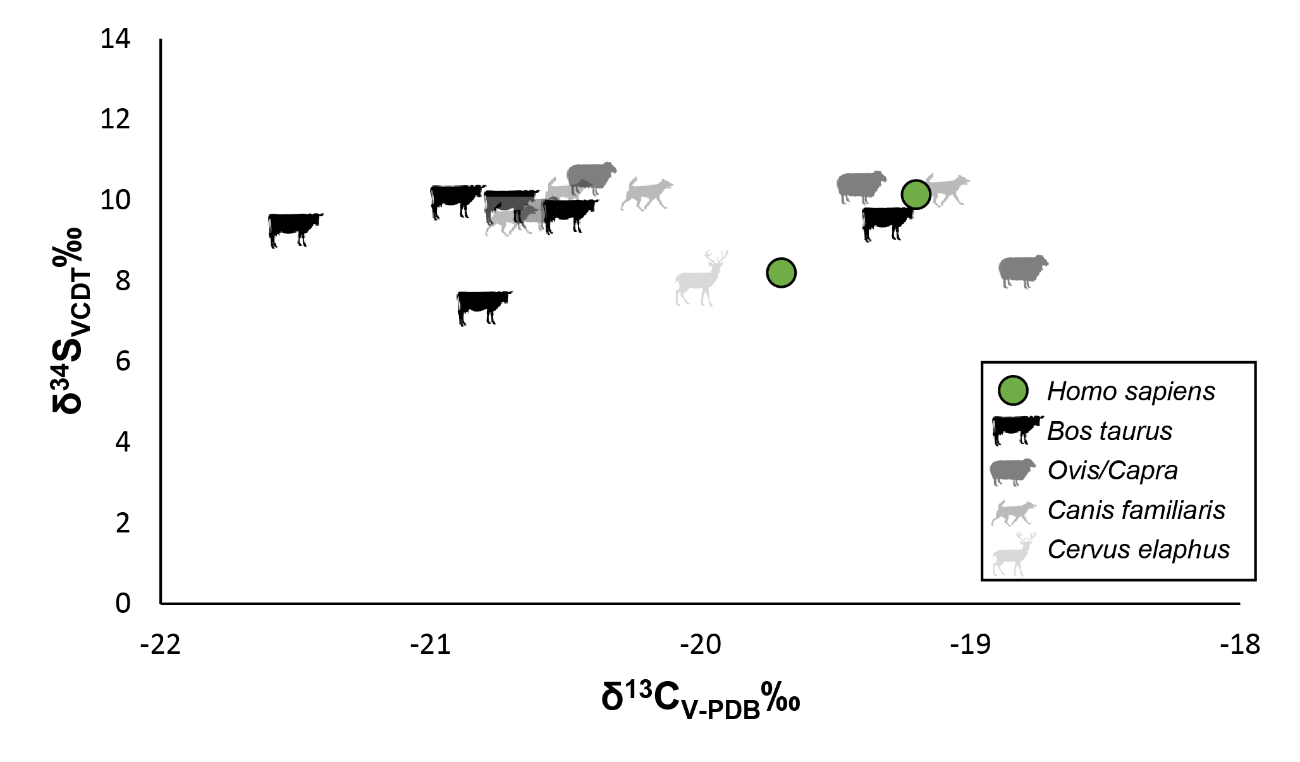


Fig. S17. Scatterplot of δ^13^C and δ^34^S values on human and animal bone collagen analysed at El Hornazo.

### 2.2.4. Fuente Celada

The Fuente Celada humans (*n=* 3), dating to the Chalcolithic, had δ^13^C values ranging between ‑19.3‰ and ‑19.5‰ and δ^15^N values ranging between 8.3‰ and 9.2‰. The tight range in values observed suggests that these individuals consumed a similar diet, including terrestrial animal protein. The faunal data show a wide range of stable isotope values. Whilst the sheep and ovicaprines (*n=* 4) had a relatively small range in δ^13^C values of between ‑20.1‰ and ‑20.7‰, the δ^15^N values were much more remarkable, between 4.3‰ and 6.8‰. The more extensive range in δ^15^N values could indicate greater diversity in the diet of these animals, potentially representing different pasturing locations or differences in winter foddering within the population analysed. The cattle from the site (*n=* 4) have a wider range in δ^13^C values between ‑20.4 and ‑21.3‰ and a smaller δ^15^N range than the ovicaprids, between 4.8‰ and 5.4‰. The wider range of δ^13^C values could also be indicative of individuals' differing management practices, such as using a variety of pastures or foddering animals on differing plants [21].


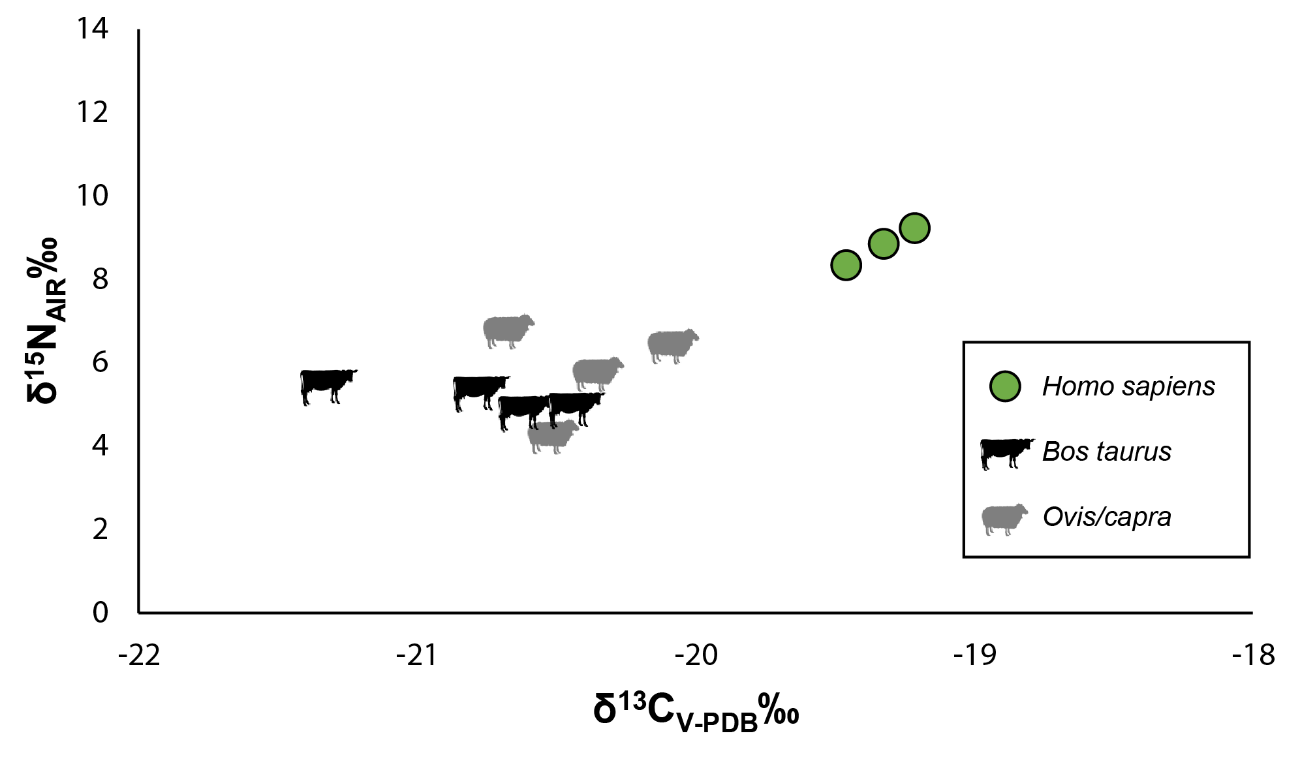


Fig. S18. Scatterplot of δ^13^C and δ^15^N values on human and animal bone collagen analysed at Fuente Celada.

The δ^34^S values of Fuente Celada humans were 9.1‰ and 10.5‰, reporting a similar sulfur isotope signature. However, the faunal specimens used for comparisons had a more heterogeneous sulfur isotope values. The cow had δ^34^S values ranging between 5.2‰ and 11‰, while ovicaprines reported sulfur isotope signals ranging between 1‰ and 11.5‰. Therefore, humans and animals provided different sulfur isotopic compositions of their bone collagen, suggesting different isozones where they were feeding. The central cluster, containing humans, two cows and two sheep, indicates these individuals lived in an area with a high δ^34^S baseline, with values ranging between 9-11‰. In comparison, the other two cows and two sheep reported lower δ^34^S values ranging between 1-7‰. Both groups reflect the probable provenance of these individuals, suggesting that the first group would be local inhabitants and the second faunal group were living in an isozone with a low δ^34^S baseline, far from the site where they were buried.


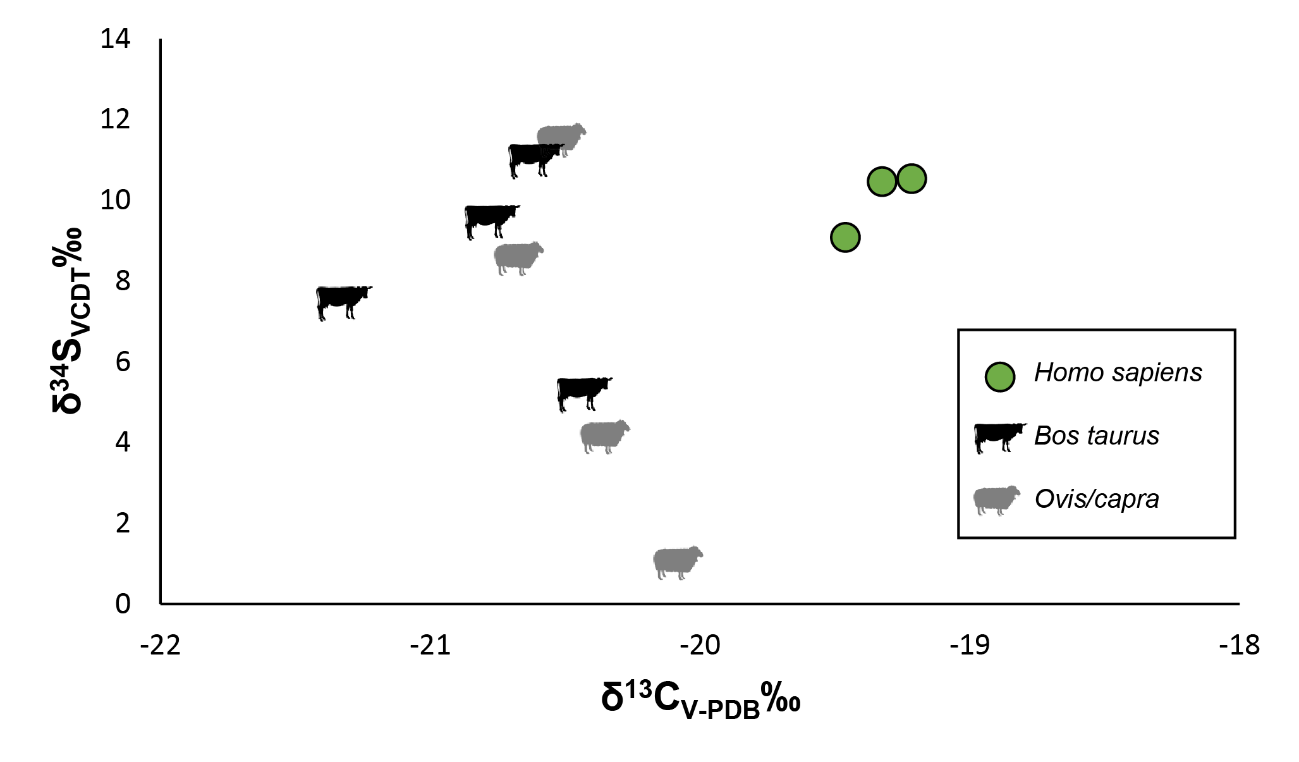


Fig. S19. Scatterplot of δ^13^C and δ^34^S values on human and animal bone collagen analysed at Fuente Celada.

### 2.2.5. Kaite

The humans analysed from Kaite (*n=* 4) were dated to the Late Neolithic (*n=* 3) and Chalcolithic (*n=* 1) cultures, and they showed δ^13^C values ranging between ‑20.5‰ and ‑19.9‰, and δ^15^N values between 6.6‰ and 9.3‰. Regarding the fauna samples, cattle showed δ^13^C values of ‑21.4‰ and ‑21‰, and δ^15^N values of 4.2‰; ovicaprines gave δ^13^C values of ‑21.5‰ and ‑20.3‰, and δ^15^N values of 3‰ and 4.3‰; the horse had a δ^13^C value of ‑21.6‰ and a δ^15^N value of 3‰; the wild cat had a δ^13^C of ‑23‰ and a δ^15^N value of 4.5‰; and the fox a δ^13^C value of ‑20.2‰ and a δ^15^N value of 5‰. The δ^13^C and δ^15^N isotope values of humans reflect a homogeneous diet based on consuming C_3_ terrestrial resources and animal protein. No isotope evidence of aquatic resources or C_4_ plant consumption was noted. The only exception is the juvenile individual, which has lower δ^13^C and δ^15^N values than the rest of the group. This may be because after weaning, the children are gradually incorporated into the group's diet, suggesting a slightly different diet for this individual. The human diet is supported by the stable isotope results of the fauna sampled at this site, which shows the usual relationship between carnivores and prey. At the species level, there are differences in the animals' diet analysed. Firstly, the fox has slightly higher δ^13^C and δ^15^N values, typical of an omnivore in a terrestrial ecosystem. However, the wild cat shows the lowest carbon isotope value, indicating a diet with less meat protein than the fox. The herbivores analysed showed greater homogeneity and were characteristic of an open landscape. The only exception was one of the ovicaprines, which could be feeding in a different geographical location than the rest of the herbivores, as it has a higher carbon isotope value.


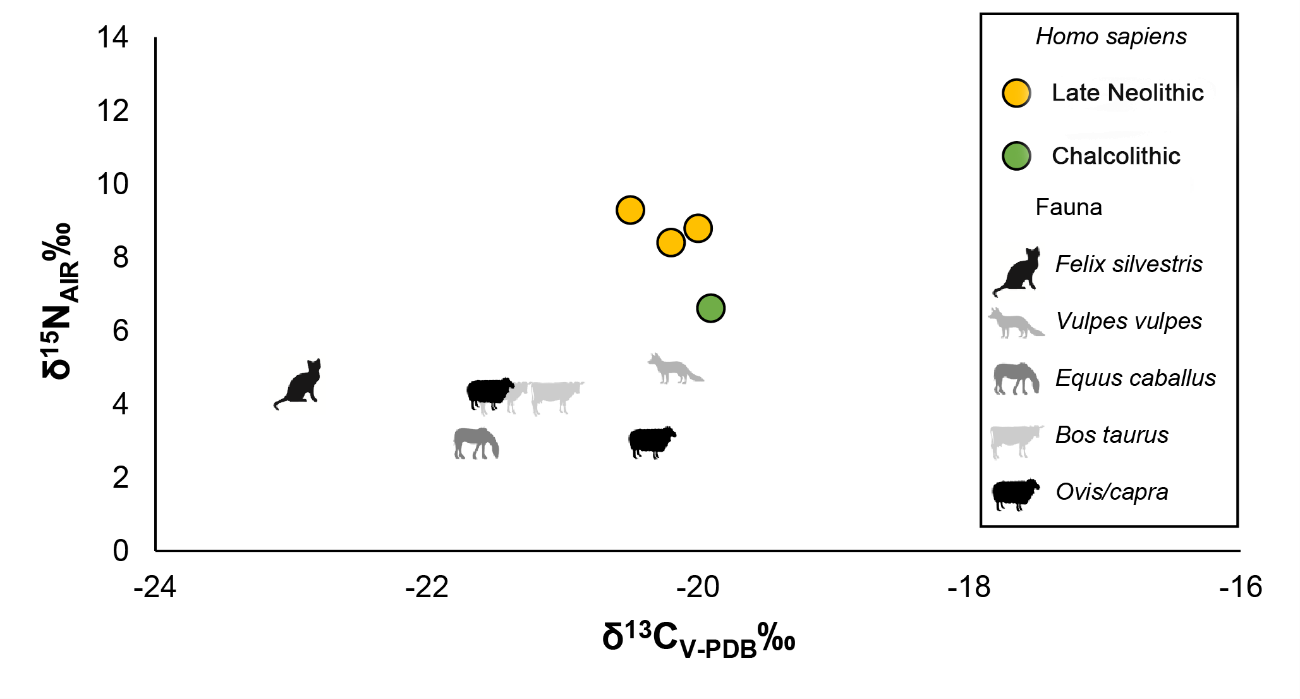


Fig. S20. Scatterplot of δ^13^C and δ^15^N values on human and animal bone collagen analysed at Kaite.

Regarding sulfur, the humans δ^34^S values ranged between 8.6‰ and 11.1‰. The cows showed δ^34^S values of 9.4‰ and 11.6‰, the ovicaprines provided δ^34^S values of 9.8‰ and 13‰, the horse had a δ^34^S of 8.8‰, the wild cat a δ^34^S of 10.2‰, and the fox a δ^34^S of 10.9‰. Therefore, all the specimens showed a limited variability in animal and human δ^34^S values, ranging between 8.6‰ and 13‰. This pattern reflects significantly lower δ^34^S values than the sites closer to the coast, indicating that the sea spray effect had limited influence on the isotope signals of these individuals, which is in line with the distance between the Cantabrian coast and this site. In contrast, these values are characteristic of inland territories, confirming that these individuals would have lived in an isozone with low bioavailable δ^34^S baseline values and suggesting the eminently local character of this human group.


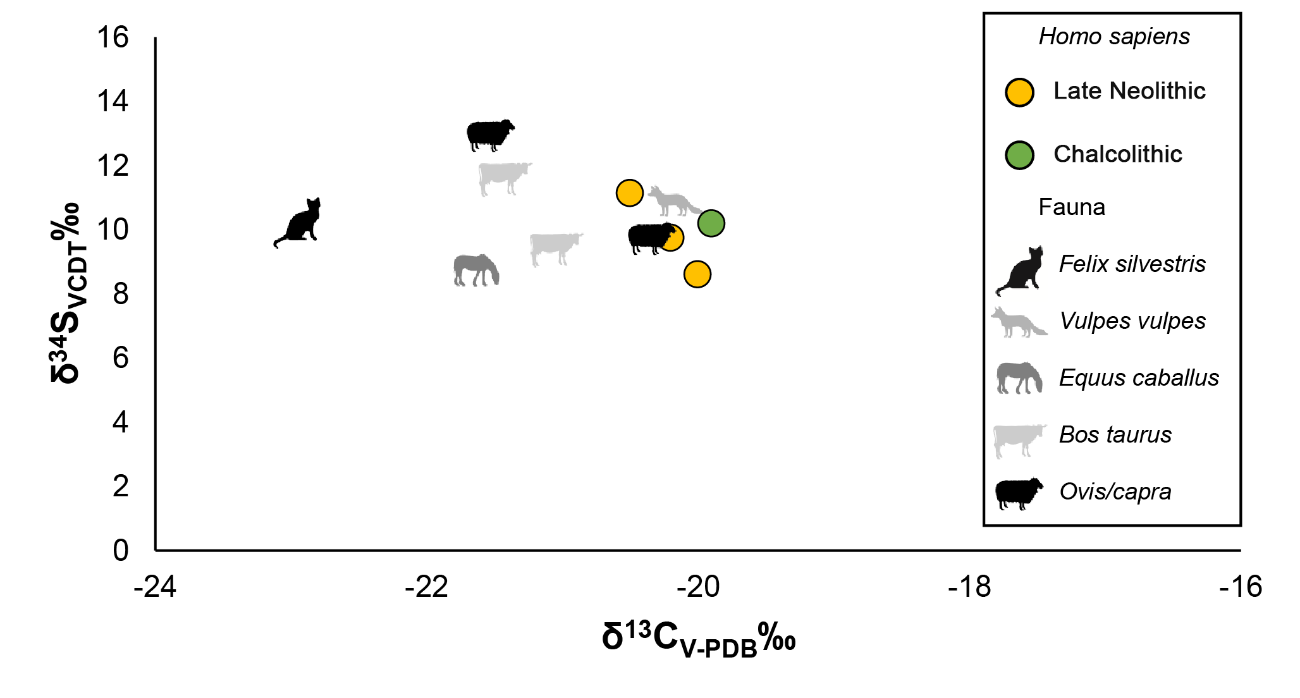


Fig. S21. Scatterplot of δ^13^C and δ^34^S values on human and animal bone collagen analysed at Kaite.

### 2.2.6. Los Cinchos

The human of this site reported a δ^13^C value of ‑20.9‰, a δ^15^N value of 8.6‰ and a δ^34^S value of 14.9‰. The δ^13^C isotope signals are characteristic of a terrestrial C_3_ plant ecosystem, while the δ^15^N value is typical of a carnivore in the terrestrial food chain. These results suggest a mixed terrestrial diet based mainly on the intake of C_3_ plants and meat protein, without isotope evidence of marine resource or C_4_ plants consumptions, which fits the rest of the Bronze individuals analysed so far. On the other hand, the δ^34^S value is very high, indicating that this individual lived the last years of his life in an isozone with high δ^34^S baseline values. These values usually appear in diets that are greatly influenced by marine resources in coastal regions due to the effect of marine spray or in certain types of evaporitic rocks. This individual's carbon and nitrogen isotope values do not support that the individual's diet resulted in its high δ^34^S value. This site is located on the southern limit of the Cantabrian region, more than 60 km from the current coastline, so it seems plausible that this individual comes from the coastal area. However, this hypothesis cannot be confirmed given the absence of comparative fauna from the same site [7].


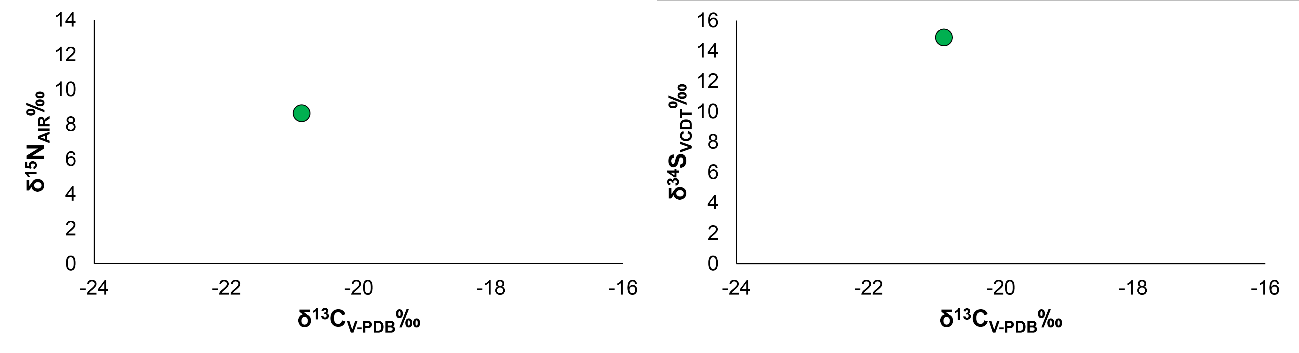


Fig. S22. Scatterplot of δ^13^C and δ^15^N, and δ^13^C and δ^34^S values on human bone collagen analysed at Los Cinchos.

### 2.2.7. Palomera

The two adult males buried at Cueva Palomera (SUC773 and SUC774) reported δ^13^C values of ‑19.8‰ and ‑19.6‰, δ^15^N values of 10.2‰ and 9.1‰, and δ^34^S values of 7.6‰ and 8.1‰. The δ^13^C isotope values indicate that these humans fed in a terrestrial ecosystem of C_3_ plants, while the δ^15^N values are typical of carnivores in the terrestrial food web. Regarding sulfur, both individuals have reported significantly low δ^34^S values. These values are expected for an area located inland, far from the coastline and with a drier climate and environment, such as the one surrounding the site, suggesting that these individuals are locals and would have lived the last years of their lives in an inland area.


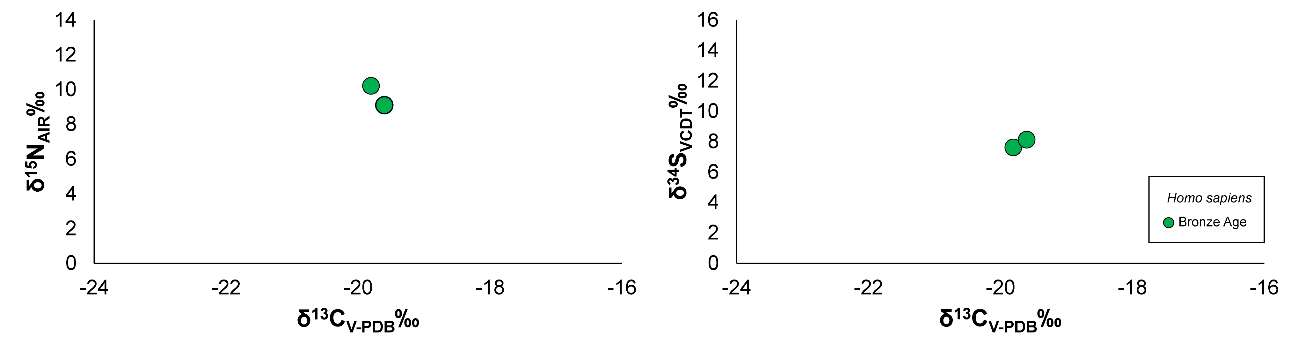


Fig. S23. Scatterplot of δ^13^C and δ^15^N, and δ^13^C and δ^34^S values on human bone collagen analysed at Cueva Palomera.

### 2.2.8. La Quebrantada

Humans from La Quebrantada cave (*n=* 19) belong to three cultural periods: Late Neolithic (*n=* 4), Chalcolithic (*n=* 1) and Bronze Age (*n=* 14). They reported δ^13^C values ranging between ‑19.9‰ and ‑19.1‰ and δ^15^N values between 7.7‰ and 10.8‰. Regarding the fauna samples, cattle showed δ^13^C values of ‑21.0‰ and ‑21.4‰, and δ^15^N values of 5.8‰ and 5.5‰; and ovicaprines had δ^13^C values of ‑21.0‰ and ‑20.1‰, and δ^15^N values of 5.9‰ and 4.3‰. Finally, a red deer specimen reported a δ^13^C value of ‑20.7‰ and a δ^15^N value of 4.4‰. The δ^13^C values of La Quebrantada reflect a homogeneous diet based on consuming C_3_ terrestrial plants. However, the δ^15^N values indicate slight differences in protein consumption, especially the Late Neolithic individuals, whose nitrogen isotope values are higher than the rest of the Bronze Age group. The animals had δ^13^C values typical for a terrestrial European C_3_ ecosystem, although the cattle and one sheep had lower carbon isotope signatures than the other sheep and red deer. This pattern suggests that the first group possibly lived in a closer environment influenced by the canopy effect. In contrast, the second group showed higher carbon isotope values characteristic of an open environment. Concerning nitrogen, their δ^15^N values were homogenous and typical of the herbivore's trophic level.


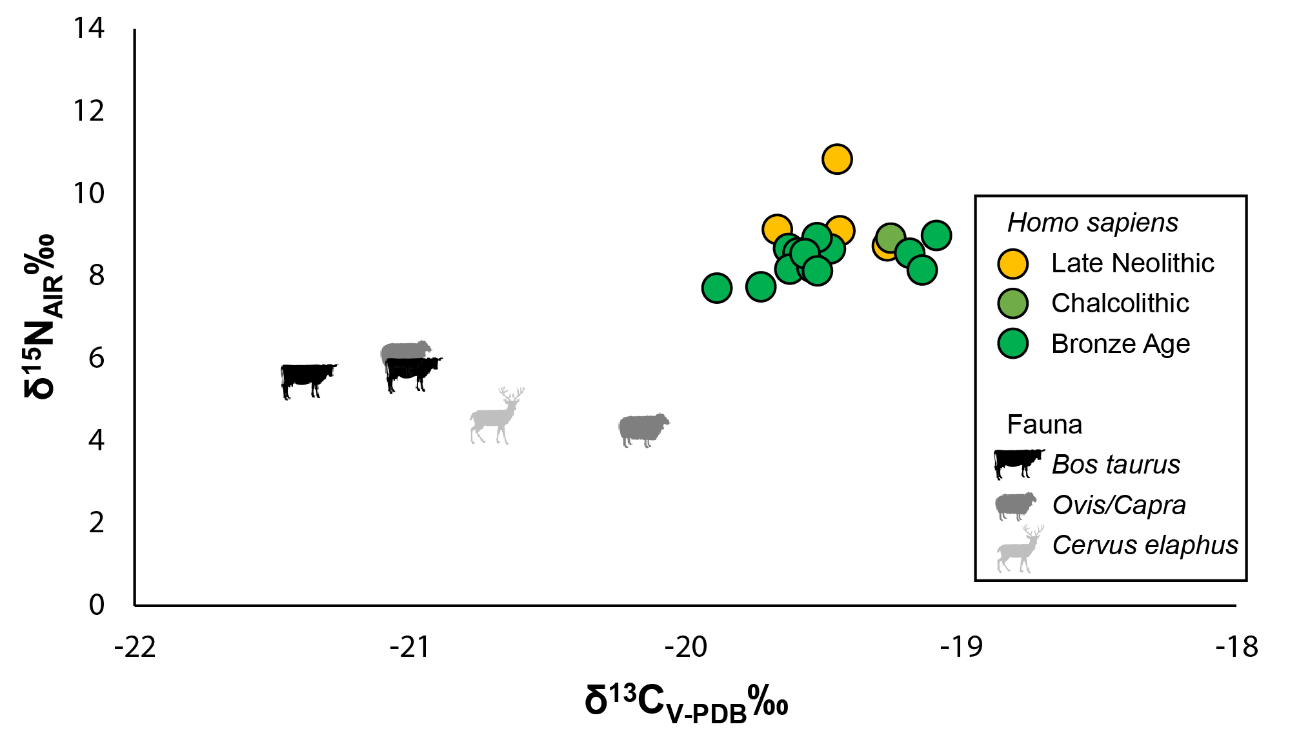


Fig. S24. Scatterplot of δ^13^C and δ^15^N values on human and animal bone collagen analysed at La Quebrantada.

Regarding sulfur, humans reported δ^34^S values ranging between 2.5‰ and 11.0‰. From the faunal samples, cattle provided δ^34^S values of 9.4‰ and 11.1‰, ovicaprines 5.5‰ and 0.9‰, and red deer a δ^34^S signature of 10‰. Most humans and animals had a similar low range of 5‰ to 11‰, indicating that they were living in an isozone with a low δ^34^S baseline. These values are coherent with the sulfur isotope values observed in inland areas. The exceptions are a human (SUC1058) and a sheep (SUC1128), whose δ^34^S values are even lower than those of the main group (2.5‰ and 0.9‰, respectively), indicating that they were likely to be non-locals and potentially derived from a more inland location.


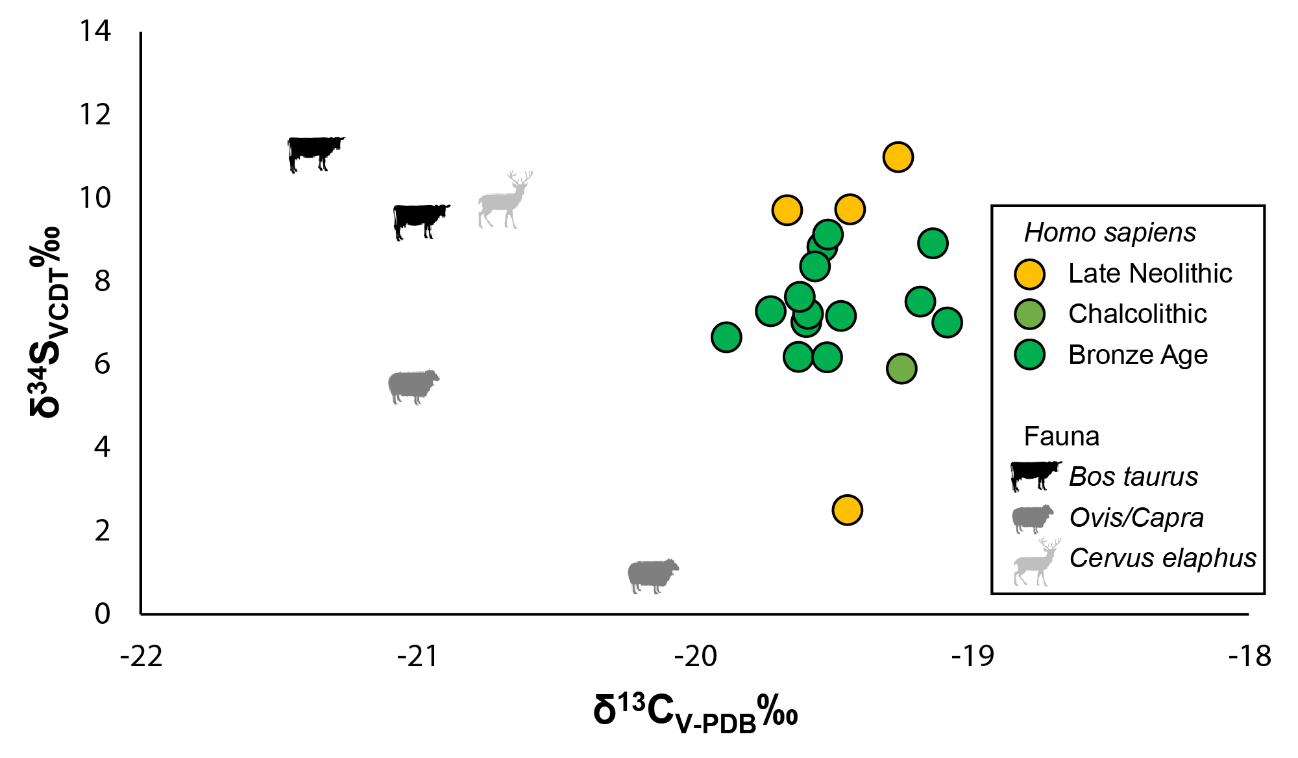


Fig. S25. Scatterplot of δ^13^C and δ^34^S values on human and animal bone collagen analysed at La Quebrantada.

### 2.2.9. Trulla

From Trulla cave, two individuals dated to the Chalcolithic (*n=* 1) and Bronze Age (*n=* 1) were isotopically analysed. They reported δ^13^C values of ‑20.4‰ and ‑19.7‰, δ^15^N values of 10.2‰ and 8.3‰, and δ^34^S values of 3.8‰ and 5.8‰. The δ^13^C isotope results indicate that these humans fed in a terrestrial ecosystem, while the δ^15^N values are typical of carnivores in a terrestrial food web. The isotope signatures would reflect a diet based on consuming C_3_ plants and animal protein. Regarding sulfur, both individuals have reported significantly low δ^34^S values, which are expected for individuals inhabiting an isozone with a low δ^34^S baseline associated with inland locations. Therefore, these individuals are potentially locals, and they might have lived in the surroundings of the site where they were buried.


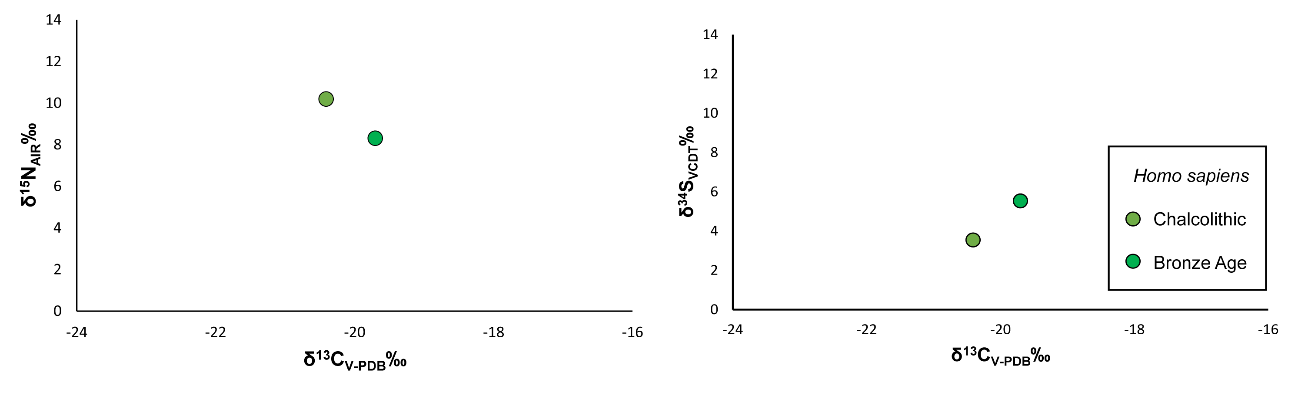


Fig. S26. Scatterplot of δ^13^C and δ^15^N, and δ^13^C and δ^34^S values on human bone collagen analysed at Trulla.

**References**

1. González-Rabanal B. Estudio arqueológico y tafonómico de los restos humanos de la cueva sepulcral de El Espinoso (Ribadedeva, Asturias). Trabajo Fin de Máster Inédito, Universidad de Cantabria. 2014.

2. González Morales MR. Memoria de los trabajos de limpieza y toma de muestras en los yacimientos de las cuevas de Mazaculos y El Espinoso (La Franca, Ribadedeva) y La Llana (Andrín, Llanes) en 1993. Excavaciones Arqueológicas en Asturias, 1991-94. Oviedo: Gobierno del Principado de Asturias; 1995. pp. 65–78.

3. Cuenca-Solana D. Utilización de instrumentos de concha para la realización de actividades productivas en las formaciones económico sociales de los cazadores-recolectores-pescadores y primeras sociedades tribales de la fachada atlántica europea. Tesis Doctoral Inédita, Universidad de Cantabria. 2012.

4. González-Rabanal B, González Morales MR, Marín-Arroyo AB. Anthropological and taphonomical study of human remains from the burial cave of El Espinoso (Ribadedeva, Asturias, Spain). In: Tomé T, Díaz-Zorita Bonilla M, Silva AM, Cunha C, Boaventura R, editors. Current Approaches to Collective Burials in the Late European Prehistory. Archaeopress; 2017. pp. 55–65.

5. González-Rabanal B. Dinámicas de población, dieta y prácticas funerarias de los últimos cazadores-recolectores y primeras sociedades campesinas de la Región Cantábrica. Tesis Doctoral Inédita, Universidad de Cantabria. 2022.

6. González-Rabanal B, González-Morales MR, Marín-Arroyo AB. La tafonomía como marco metodológico para interpretar depósitos funerarios superficiales: estudio de la cueva sepulcral de El Espinoso (Ribadedeva, Asturias). Trab Prehist. 2017;74: 278–295. <https://doi.org/10.3989/tp.2017.12195>

7. González-Rabanal B, Marín-Arroyo AB, Cristiani E, Zupancich A, González Morales MR. The arrival of millets to the Atlantic coast of northern Iberia. Scientific reports. 2022;12: 18589. <https://doi.org/10.1038/s41598-022-23227-4>

8. Patterson N, Isakov M, Booth T, Büster L, Fischer C-E, Olalde I, et al. Large-scale migration into Britain during the Middle to Late Bronze Age. Nature. 2021;601: 588–594. <https://doi.org/10.1038/s41586-021-04287-4>

9. Straus LG, González Morales MR. El Mirón Cave and the 14C Chronology of Cantabrian Spain. Radiocarbon. 2003;45: 41–58. <https://doi.org/10.1017/S0033822200032380>

10. González Morales MR, Straus LG. La Cueva del Mirón (Ramales de la Victoria, Cantabria): excavaciones 1996-1999. Trabajos de Prehistoria. 2000;57: 121–133. <https://doi.org/10.3989/tp.2000.v57.i1.264>

11. Hopkins RJA, Straus LG, González Morales MR. Assesing the chronostratigraphy of El Mirón cave, Cantabrian Spain. Radiocarbon. 2021;63: 821–852. <https://doi.org/10.1017/rdc.2020.121>

12. Straus LG, González Morales MR. El Mirón Cave, Cantabrian Spain. The site and its holocene archaeological record. Albuquerque: University of New Mexico Press; 2012.

13. González Morales MR, González-Rabanal B, Gutiérrez-Zugasti I, Cuenca-Solana D, Straus LG. Chalcolithic/Early Bronze Age and additional Magdalenian radiocarbon dates for El Mirón cave (Ramales de la Victoria, Cantabria, Spain). Date List VII. Radiocarbon. 2024; 1–14. <https://doi.org/10.1017/RDC.2023.123>

14. Gomarin Guirado F. Dos nuevos yacimientos de la Edad del Bronce en Santander. Zephyrvs. 1972;XXIII–XXIV: 193–195.

15. Rincón Vila R. Las culturas del metal. In: García Guinea MÁ, editor. Historia de Cantabria: prehistoria, edades antigua y media. Santander: Estudios; 1985. pp. 113–210.

16. Ruiz Cobo J. Implantación y desarrollo de las economías de producción en Cantabria. Tesis Doctoral Inédita, Universidad de Cantabria. 1991.

17. Vega Maeso C. La cerámica inciso-impresa en el tránsito del III al II milenio cal B.C. en la Región Cantábrica. Tesis Doctoral Inédita, Universidad de Cantabria. 2015.

18. Vega Maeso C, Carmona Ballestero E, Sierra Sainz-Aja A, García Rojo A. The domestic use of the Abrigo de la Castañera (Cantabria, Spain) during the Neolithic (4th-5th millenia cal BC). VII Congreso Internacional del Neolítico en la Península Ibérica. Universidad de Sevilla; 2020.

19. Vega-Maeso C, Carmona-Ballestero E, Sierra Sainz-Aja A, Marín-Arroyo AB. El Abrigo de la Castañera (Cantabria, Spain): A Chalcolithic cattle stable? Quat Int. 2016;414: 226–235. <https://doi.org/10.1016/j.quaint.2015.09.047>

20. Sierra Sainz-Aja A. Subsistencia y modos de vida durante la Prehistoria Reciente en Cantabria: los restos arqueofaunísticos y antropológicos del Abrigo de la Castañera. Trabajo Fin de Máster Inédito, Universidad de Cantabria. 2014.

21. Jones JR, Maeso CV, Ballestero EC, Martín LV, Arceo MED, Marín-Arroyo AB. Investigating prehistoric diet and lifeways of early farmers in central northern Spain (3000–1500 CAL BC) using stable isotope techniques. Archaeol Anthropol Sci. 2019;11: 3979–3994. <https://doi.org/10.1007/s12520-019-00841-4>

22. González Morales MR. La prehistoria de las marismas: Excavaciones en la cueva de La Fragua (Santoña). Campañas de 1990, 1991, 1993, 1994 y 1996). Actuaciones Arqueológicas en Cantabria, 1984-1999. Santander: Gobierno de Cantabria; 2000. pp. 177–180.

23. González Morales MR. La Prehistoria del Valle del Asón: un resumen de 30 años de investigaciones. Sautuola. 2015;XX: 53–72.

24. Marín Arroyo AB, González Morales MR, Estévez J. Paleoclimatic inference of the mid-Holocene record of monk seal (Monachus monachus) in the Cantabrian Coast. Proc Geol Assoc. 2011;122: 113–124. <https://doi.org/10.1016/j.pgeola.2010.11.001>

25. Marín-Arroyo AB. Análisis arqueozoológico, tafonómico y de distribución espacial de la fauna de mamíferos de la Cueva de la Fragua (Santoña, Cantabria). Munibe Antropología-Arkeología. 2004;56: 19–44.

26. Pérez Suárez C. Carta Arqueológica de los concejos de Llanes y Ribadedeva (1992). Excavaciones Arqueológicas en Asturias, 1991-94. Oviedo: Gobierno del Principado de Asturias; 1995. pp. 243–245.

27. Vega Maeso C, Gallello G, Palmero S, Ferrari B. Ceramic productions and human interactions during the Early Bronze Age in northern Iberia. Archaeometry. 2021;63: 68–87. <https://doi.org/10.1111/arcm.12605>

28. Estalrrich A, González-Rabanal B, Marín-Arroyo AB, Maeso CV, González Morales MR. Osteolytic lesions on the os petrosum of a Bronze Age individual from La Llana cave (Northern Spain) compatible with a possible case of otitis media. A multifaceted methodological approach. International Journal of Paleopathology. 2020;31: 97–102. <https://doi.org/10.1016/j.ijpp.2020.10.006>

29. Muñoz E, Gómez J, San Miguel C. Catálogo topográfico de las cavidades con interés arqueológico: Ruiloba-Besaya (Zona III). Boletín Cántabro de Espeleología. 1993; 57–73.

30. Begines A, García Cáraves JM. Hallazgos del Bronce I en dos cuevas de Santander. Actas del IX Congreso Nacional de Arqueología. 1966. pp. 122–126.

31. Muñoz E, San Miguel C. Carta arqueológica de Cantabria. Tantín, Santander. 1988.

32. González-Rabanal B, Marín-Arroyo AB, Jones JR, Agudo Pérez L, Vega-Maeso C, González Morales MR. Diet, mobility and death of Late Neolithic and Chalcolithic groups of the Cantabrian Region (northern Spain). A multidisciplinary approach towards studying the Los Avellanos I and II burial caves. Journal of Archaeological Science: Reports. 2020;34: 1–13. <https://doi.org/10.1016/j.jasrep.2020.102644>

33. Carmona Ballestero E. Calcolítico en la cuenca media del Arlanzón (Burgos, España): comunidades campesinas, procesos históricos y transformaciones. Oxford: Archaeopress; 2013.

34. Carmona Ballestero E, Arnáiz Alonso MÁ, Cuenca-Romero M del CA. El dolmen de Arroyal I: usos y modificaciones durante el III milenio cal AC. In: Honrado Castro JH, Brezmes Escribano MA, Tejeiro Pizarro A, Rodríguez Monterrubio O, editors. Investigaciones Arqueológicas en el valle del Duero. 2014. pp. 41–54.

35. Mateo Pellitero P. El Dolmen del Arroyal I (Burgos): estudio arqueozoológico y tafonómico de los restos de macromamíferos neolíticos y calcolíticos. Trabajo Fin de Máster. Universidad de Cantabria.

36. Olalde I, Brace S, Allentoft ME, Armit I, Kristiansen K, Booth T, et al. The Beaker phenomenon and the genomic transformation of northwest Europe. Nature. 2018;555: 190–196. <https://doi.org/10.1038/nature25738>

37. Carmona Ballestero E, Valdivielso Gutiérrez E, Pascual Blanco S, Vega y Miguel J. Restos humanos, contextos funerarios y diversidad formal: Los yacimientos calcolíticos de El Hornazo y El Túmulo IL.C1 de Cótar (Burgos). Rev Atlántica-Mediterránea Prehist Arqueol Soc. 2013.

38. Alameda Cuenca-Romero M del C, Carmona Ballestero E, Pascual Blanco S, Martínez Díez G, Díez Pastor C. El “campo de hoyos” calcolítico de Fuente Celada (Burgos): datos preliminares y perspectivas. Complutum. 2011;22: 47–69. <https://doi.org/10.5209/rev_CMPL.2011.v22.n1.3>

39. Olalde I, Mallick S, Patterson N, Rohland N, Villalba-Mouco V, Silva M, et al. The genomic history of the Iberian Peninsula over the past 8000 years. Science. 2019;363: 1230–1234. <https://doi.org/10.1126/science.aav4040>

40. Ortega AI, Martín MÁ. La Arqueología del Karst de Ojo Guareña. In: Grupo Espeleológico Edelweiss, editor. Monografía sobre Ojo Guareña. Kaite, Estudios de Espeleología Burgalesa; 1986. pp. 331–389.

41. Uribarri JL, Liz C. El arte rupestre de" Ojo Guareña". La cueva de Kaite. Trabajos de Prehistoria. 1973;30: 69–120.

42. Ortega AI, Martín MÁ, Grupo Espeleológico Edelweiss. Cuevas de Ojo Guareña. Una visión de la mano del Grupo Espeleológico Edelweiss. Diputación Provincial de Burgos; 2013.

43. García de Castro C, Busto Hevia G. Hallazgo y extracción de un esqueleto humano de la Edad del Bronce en la cueva de la Paré los Cinchos (Puertu Güeria, Quirós, Asturias). Excavaciones Arqueológicas en Asturias, 2013-2016. Oviedo: Gobierno del Principado de Asturias; 2018. pp. 183–192.

44. Alonso-Llamazares C, López Martínez B. Estudio antropológico de los restos óseos humanos recuperados en el macizo de Ubiña. Excavaciones Arqueológicas en Asturias, 2013-2016. Oviedo: Gobierno del Principado de Asturias; 2018. pp. 193–204.

45. Karampaglidis T, Benito-Calvo A, Ortega-Martínez AI, Martín-Merino MÁ, Sánchez-Romero L. Landscape evolution and the karst development in the Ojo Guareña multilevel cave system (Merindad de Sotoscueva, Burgos, Spain). J Maps. 2023;19: 1–14. <https://doi.org/10.1080/17445647.2022.2128907>

46. Camacho AI, Puch C. Ojo Guareña: A Hotspot of Subterranean Biodiversity in Spain. Diversity. 2021;13: 199. <https://doi.org/10.3390/d13050199>

47. Ortega AI, Martín MÁ. Investigaciones de Adolfo Eraso en Ojo Guareña y Atapuerca y síntesis del conocimiento actual del karst en ambas zonas. In: SEDECK, editor. Homenaje a Adolfo Eraso Romero, perfil humano y científico. Sociedad Española de Espeleología y Ciencias del Karst (SEDECK); 2023. pp. 35–88.

48. González-Rabanal B, Marín-Arroyo AB, Vidal-Cordasco M, Martín MÁ, Ortega AI. Early Bronze Age violence in Ojo Guareña (Merindad de Sotoscueva, Burgos, Spain). Perimortem modifications in two male individuals. Quaternary Science Advances. 2023;12: 100120. <https://doi.org/10.1016/j.qsa.2023.100120>

49. Ortega AI, Ruiz F, Martín MÁ, Benito-Calvo A, Vidal M, Bermejo L, et al. Prehistoric Human Tracks in Ojo Guareña Cave System (Burgos, Spain): The Sala and Galerías de las Huellas. In: Pastoors A, Lenssen-Erz T, editors. Reading Prehistoric Human Tracks: Methods & Material. Cham: Springer International Publishing; 2021. pp. 317–342.

50. Ortega AI, Martín MÁ. El arte rupestre de Ojo Guareña: singularidad y pervivencia en el tiempo. Cubía. 2015;19: 10–23.

51. Ortega AI, Martín MÁ, García-Diez M. Palaeolithic creation and later visits of symbolic spaces: radiocarbon AMS dating and cave art in the Sala de las Pinturas in Ojo Guareña (Burgos, Spain). Archaeol Anthropol Sci. 2020;12: 240. <https://doi.org/10.1007/s12520-020-01208-w>

52. Gómez-Barrera JA, Ortega AI, Martín MÁ, García M, Fernández JJ, Val J del. Las manifestaciones gráficas de la Sala de la Fuente (Ojo Guareña, Burgos): dataciones absolutas para la contextualización del arte rupestre. Boletín del Seminario de Estudios de Arte y Arqueología 2003;LXVI(2000): 65-79.

53. Ortega AI, Ruiz F, Martín MÁ, Benito A, Vidal M, García M. (2021): Nuevas investigaciones y dataciones arqueológicas en Ojo Guareña (Merindad de Sotoscueva, Burgos). SEDECK. 2021;16: 51-84.

54. Ortega AI, Molina A, Martín MÁ. La Cueva de La Quebrantada y el entorno de Santa Cecilia (Montorio). Cubía. 2020;24: 28–35.

55. Ortega A I. Simbolismo y contexto cronológico de las cavidades sepulcrales de Montorio (Burgos): Pigmentos rojos y Cerámicas de prestigio. Memoria Técnica; Ayuntamiento de Montorio, Diputación Provincial. Inédito, 2023; 63 pp.
